# Organic Electrochemical Transistors for Neuromorphic Bioelectronics: Materials and Manufacturing Technologies

**DOI:** 10.1002/advs.77655

**Published:** 2026-09-16

**Authors:** Hyunjin Lee, Jihyang Park, Xinyi Wang, Yueqi Deng, Jiashu Ren, Tao Zhou

**Affiliations:** ^1^ Department of Biomedical Engineering The Pennsylvania State University University Park Pennsylvania USA; ^2^ Department of Engineering Science and Mechanics The Pennsylvania State University University Park Pennsylvania USA; ^3^ Department of Mechanical Engineering The Pennsylvania State University University Park Pennsylvania USA; ^4^ Center for Neural Engineering The Pennsylvania State University University Park Pennsylvania USA; ^5^ Huck Institutes of the Life Sciences The Pennsylvania State University University Park Pennsylvania USA; ^6^ Materials Research Institute The Pennsylvania State University University Park Pennsylvania USA

**Keywords:** additive manufacturing, neuromorphic bioelectronics, organic electrochemical transistors, organic mixed ionic–electronic conductors, synaptic plasticity, volumetric electrochemical doping

## Abstract

Neuromorphic bioelectronics operate in soft, hydrated environments where ionic flux, electronic transport, and molecular reconfiguration jointly encode history‐dependent computation. Organic mixed ionic–electronic conductors provide a materials platform that supports concurrent ionic and electronic transport within the same bulk medium, enabling conductance to evolve continuously under electrochemical stimuli. This review examines organic electrochemical transistors as a unifying device architecture in which electrochemical gating induces volumetric (de)doping, coupling channel geometry and electrolyte accessibility to ionic time constants, nonlinearity, retention or volatility, and energy dissipation. Materials coverage includes conducting polymers such as poly(3,4‐ethylenedioxythiophene): polystyrene sulfonate (PEDOT:PSS), glycolated conjugated semiconductors, donor–acceptor mixed conductors enabling n‐type and complementary operation, and semiconducting hydrogels and composites engineered for aqueous stability and tissue‐like mechanical compliance. Manufacturing strategies ranging from microfabrication and lithographic patterning to printing‐based additive manufacturing (AM) and hybrid process flows are discussed, with emphasis on their roles in defining planar and vertical device architectures and electrolyte integration. Neuromorphic functionalities are analyzed across hierarchical levels, spanning synaptic primitives including excitatory postsynaptic current, short‐term plasticity, long‐term plasticity, and spike‐timing‐dependent plasticity, as well as artificial neurons, reservoir computing, and closed‐loop biohybrid sensing–processing interfaces.

## Graphical abstract

Organic electrochemical transistors bridge biological ionic signaling and neuromorphic computation through volumetric electrochemical gating. This review connects Organic mixed ionic–electronic conductor materials, device architectures, and manufacturing technologies to show how geometry, ion transport, and fabrication strategies program plasticity, memory, and adaptive functions in soft biointegrated electronics.

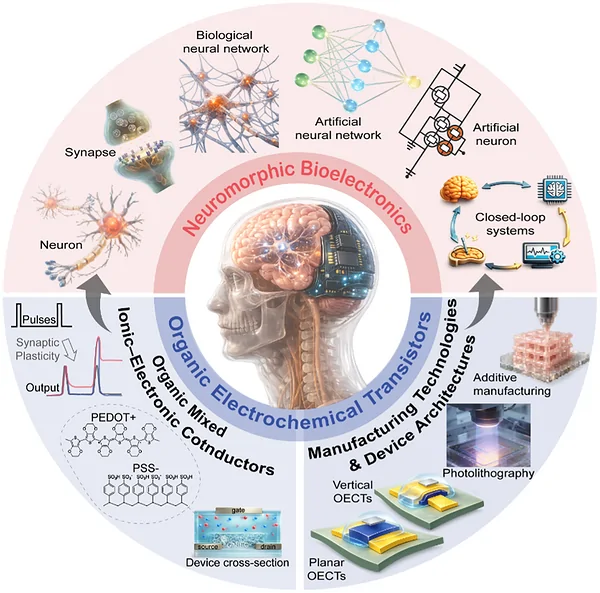

**Abbreviations**
AJP: aerosol jet printing
AM: additive manufacturing
CMOS: complementary metal–oxide–semiconductor
CVD: chemical vapor deposition
DIW: direct ink writing
ECG: electrocardiography
EMG: electromyography
ENODe: electrochemical neuromorphic organic device
EPSC: excitatory postsynaptic current
FET: field‐effect transistor
LTD: long‐term depression
LTM: long‐term memory
LTP: long‐term plasticity
OAN: organic artificial neuron
OECT: organic electrochemical transistor
OECN: organic electrochemical neuron
OMIEC: organic mixed ionic–electronic conductor
PPD: paired‐pulse depression
PPF: paired‐pulse facilitation
PPR: paired‐pulse ratio
PSC: postsynaptic current
PVD: physical vapor deposition
SNDP: spike‐number‐dependent plasticity
STDP: spike‐timing‐dependent plasticity
STM: short‐term memory
STP: short‐term plasticity

## 1 Introduction

### 1.1 Neuromorphic Hardware Landscape and Bioinspired Motivation

Neuromorphic engineering aims to physically implement the computational principles of biological neural systems within electronic hardware. Unlike conventional digital electronics, where information processing is governed by rigid semiconductor platforms and binary switching dictated by electronic band structure, biological computation emerges from distributed ionic fluxes, electrochemical gradients, and dynamically reconfigurable molecular environments operating across multiple timescales [1, 2]. Neural signaling and synaptic plasticity are intrinsically analog, history‐dependent, and highly energy‐efficient processes that arise from the coupled transport of ions and electrons within soft, hydrated biological media.

Over the past decade, significant progress has been achieved using complementary metal‐oxide‐semiconductor (CMOS)‐based analog circuits, inorganic memristors, phase‐change materials, and filamentary resistive switching devices to emulate key neuromorphic functions, including synaptic plasticity and spike‐based information processing [3, 4]. These technologies have enabled hardware implementations of in‐memory computing and event‐driven architectures. However, their operation remains fundamentally rooted in purely electronic charge transport within rigid solid‐state matrices. Consequently, although such systems reproduce certain computational behaviors of neural circuits, they do not replicate the ion‐mediated electrochemical physics that underlies biological information processing [5, 6, 7, 8].

This physical mismatch becomes particularly significant as neuromorphic technologies increasingly move toward bionic and bioelectronic applications. Devices designed to interface with neural tissue need to operate within aqueous, ionic, and mechanically compliant environments in which signal transduction is inherently electrochemical. Bridging biological ionic signaling and electronic computation therefore requires material platforms capable of intrinsically coupling ionic and electronic transport within soft matter systems.

### 1.2 Organic Electrochemical Transistors as a Distinct Neuromorphic Platform

Organic mixed ionic–electronic conductors (OMIECs) are soft electronic materials that enable simultaneous transport of ionic and electronic charge carriers within the same bulk volume. Unlike conventional semiconductors, whose conductivity is governed solely by electronic carriers, OMIECs allow ions from an electrolyte to modulate electronic carrier density throughout the material, establishing a direct interface between electrochemical processes and electronic signal transduction. Organic electrochemical transistors (OECTs) exploit this property in a three‐terminal architecture in which an electrolyte gate drives ions into or out of an OMIEC channel. Ionic penetration modulates channel conductivity through reversible electrochemical doping and dedoping, enabling low‐voltage operation in aqueous environments [9, 10].

A defining feature of OECT operation is volumetric electrochemical gating. In contrast to field‐effect transistors (FETs), where charge accumulation is confined to an interfacial layer, ions in OECTs penetrate the bulk of the mixed‐conducting polymer channel. This volumetric interaction produces distributed electrostatic compensation and redox reactions throughout the material, leading to gradual and history‐dependent modulation of conductance governed by ionic transport and electrochemical relaxation dynamics [11, 12, 13].

These electrochemical dynamics naturally enable neuromorphic functionality. Rather than abrupt binary switching, OECT conductance evolves continuously in response to the temporal history of ionic and electrical stimuli, providing a physical basis for synaptic plasticity, temporal integration, nonlinear adaptation, and analog signal processing.

Recent advances in OMIEC molecular design, including side‐chain engineering, charge‐density optimization, and complementary n‐type mixed conductors, have expanded the tunability of ionic–electronic coupling and device dynamics [14, 15]. Hydrogel‐based semiconductors and ultrasoft mixed conductors have further extended OECT operation into tissue‐like mechanical regimes, enabling stable bioelectronic interfaces [16]. Improvements in device architecture, in operando characterization, and scalable fabrication have also enhanced reproducibility, stability, and system‐level integration [17].

**FIGURE 1:**
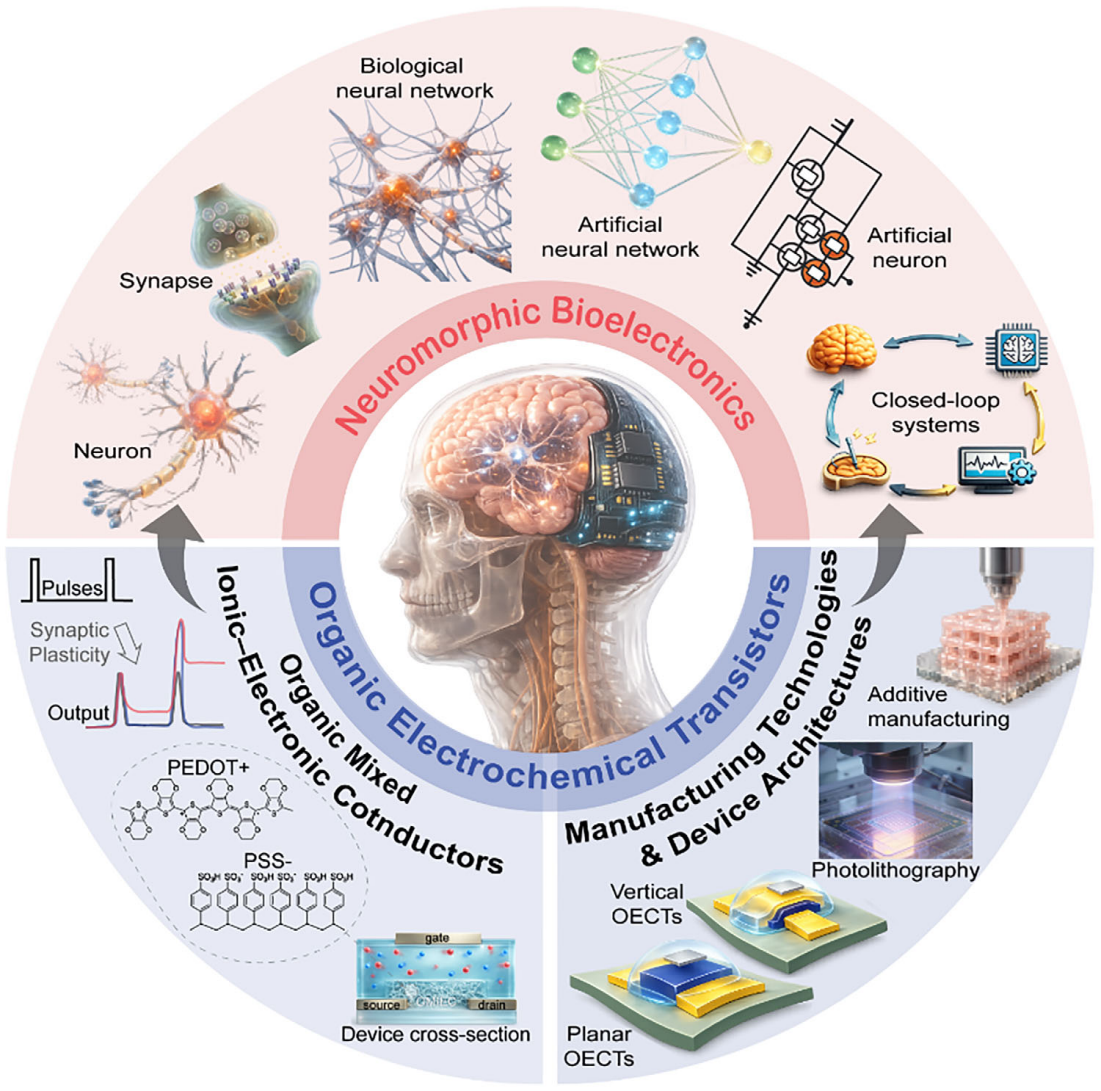
Conceptual framework of OECT‐based neuromorphic bioelectronics. Schematic overview illustrates the hierarchical integration of biological principles, organic mixed ionic–electronic materials, and manufacturing‐enabled device architectures in OECT‐based neuromorphic systems. The upper hemisphere depicts biological neural networks, synapses, and neurons alongside their artificial counterparts, including artificial neural networks, artificial neurons, and closed‐loop systems, emphasizing the translation of biological information processing into hardware implementations. The lower‐left quadrant highlights organic mixed ionic–electronic conductors (OMIECs), exemplified by PEDOT:PSS, and the underlying ionic–electronic coupling that enables volumetric electrochemical gating, synaptic plasticity, and history‐dependent conductance modulation. The lower‐right quadrant presents manufacturing technologies and device architectures, including photolithography, AM, and planar and vertical OECT geometries, demonstrating how fabrication strategies define channel geometry, electrolyte organization, and ion transport pathways. Together, these interconnected domains establish a co‐design framework spanning materials, architecture, and manufacturing that underpins synaptic plasticity, artificial neuron operation, network‐level computation, and closed‐loop bioelectronic control in OECT‐based neuromorphic bioelectronics.

As illustrated in Figure 1 (lower hemisphere), OECT‐based neuromorphic bioelectronics emerge from the convergence of bioinspired neural principles with organic mixed ionic–electronic materials, shaped by device architectures and manufacturing technologies. These domains collectively define hierarchical OECT implementations spanning artificial synapses, artificial neurons, neuromorphic networks, and closed‐loop bioelectronic systems.

### 1.3 Materials and Manufacturing as Determinants of Neuromorphic Function

A defining characteristic of OECT‐based neuromorphic systems is that device functionality does not arise solely from material composition. Instead, neuromorphic behavior is determined by the coupled interplay between ionic–electronic material properties, device geometry, electrolyte configuration, and fabrication strategy [18].

For example, the thickness and morphology of the OMIEC channel govern volumetric capacitance and ionic penetration depth, thereby influencing device gain, response time, and retention characteristics. Device architectures, such as planar and vertical configurations, further determine the relative contributions of interfacial gating and bulk electrochemical modulation [19]. The organization and composition of the electrolyte regulate ion supply, transport kinetics, and electrochemical stability [20].

As highlighted in Figure 1 (lower‐right quadrant), manufacturing technologies including lithographic patterning, additive manufacturing (AM), multilayer printing, and hybrid integration actively define these structural parameters. Consequently, fabrication methods directly influence ionic transport pathways, electrochemical dynamics, and the resulting neuromorphic behaviors, including hysteresis, plasticity, and nonlinear signal processing [21, 22, 23, 24].

In this context, manufacturing transcends its traditional role as a processing step and instead becomes a programmable design variable that governs device physics and functional behavior. Emerging AM strategies further expand this design space by enabling multi‐material integration, three‐dimensional architectures, and mechanically compliant substrates. These capabilities are accelerating the development of OECT platforms for wearable, implantable, and closed‐loop bioelectronic systems. The convergence of materials chemistry, electrochemical device physics, and scalable manufacturing therefore establishes a unique co‐design paradigm for OECT‐based neuromorphic bioelectronics.

### 1.4 Scope and Content Structure

Despite rapid progress, research on OECT‐based neuromorphic bioelectronics remains fragmented across several disciplines, including materials chemistry, device physics, manufacturing technologies, and application‐driven system demonstrations. Developing a cohesive framework that connects ionic–electronic material fundamentals with geometry‐dependent electrochemical dynamics and fabrication‐enabled architectural control is therefore essential.

Figure 1 summarizes this perspective by linking biological neural components and their artificial counterparts (upper hemisphere) with the material and technological foundations of OECT systems (lower hemisphere). Specifically, OMIEC‐based ionic–electronic coupling provides the fundamental mechanism for neuromorphic functionality, while manufacturing‐enabled device architectures regulate the resulting electrochemical dynamics and system‐level behavior.

In this review, we present a materials‐centered and manufacturing‐aware framework for understanding OECT‐based neuromorphic bioelectronics. Section 2 examines the physical foundations of organic mixed ionic–electronic conductors, including ionic–electronic coupling mechanisms, volumetric electrochemical gating, and temporally adaptive responses. Section 3 discusses manufacturing technologies and device architectures, emphasizing how fabrication strategies regulate device physics and neuromorphic functionality. Section 4 surveys hierarchical system‐level implementations, including artificial synapses, artificial neurons, neuromorphic networks, in‐sensor computing systems, and closed‐loop bioelectronic platforms. Finally, Section 5 outlines current challenges and future opportunities in scalable manufacturing, operational stability, complementary integration, and long‐term biointerface deployment.

By integrating advances in materials design, electrochemical transport physics, and manufacturing‐enabled architecture engineering, this review highlights how OECT platforms uniquely bridge the gap between biological ionic computation and scalable electronic implementation, establishing a foundation for adaptive and biointegrated neuromorphic systems.

**FIGURE 2:**
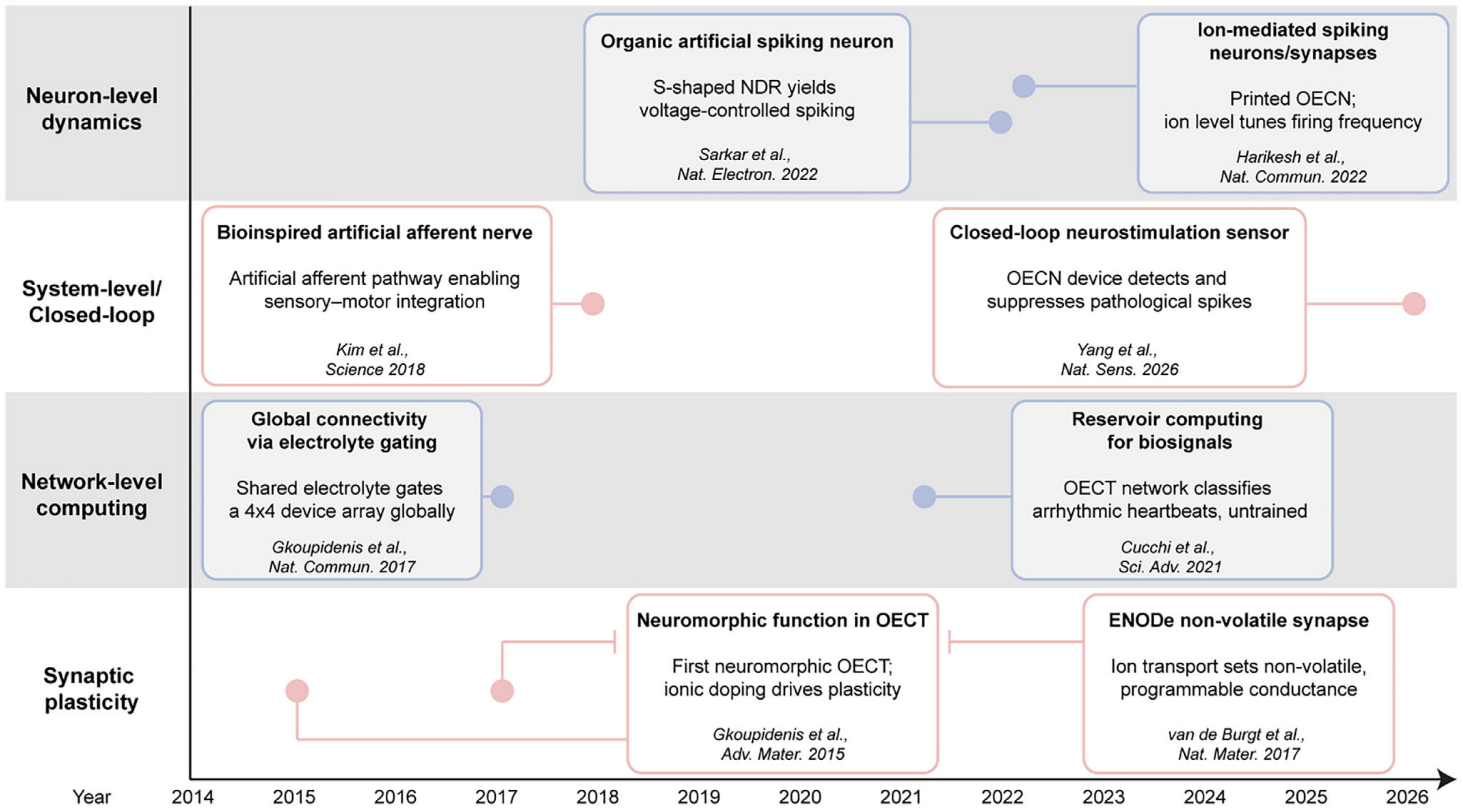
Chronological milestones in OECT‐based neuromorphic applications, organized by functional categories and year of publication. Four categories are shown: synaptic plasticity (learning and memory functions) [31, 32]; network‐level computing (distributed information processing) [155, 186]; system‐level/closed‐loop integration (adaptive sensing and actuation) [188, 189]; and neuron‐level dynamics (spiking and temporal integration) [180, 182].

While Figure 1 establishes the conceptual and hierarchical framework linking biological principles to OECT‐based implementation, Figure 2 complements this by tracing the chronological development of representative milestones across the same four functional categories, from early demonstrations of synaptic plasticity to recent closed‐loop biointegrated systems. This timeline highlights that progress has not followed a strictly sequential hierarchy; rather, distinct functional categories have advanced asynchronously, with network‐level and system‐level demonstrations in some cases preceding device‐level neuron‐scale spiking dynamics.

## 2 Organic Mixed Ionic–Electronic Conductors for Neuromorphic Bioelectronics

**FIGURE 3:**
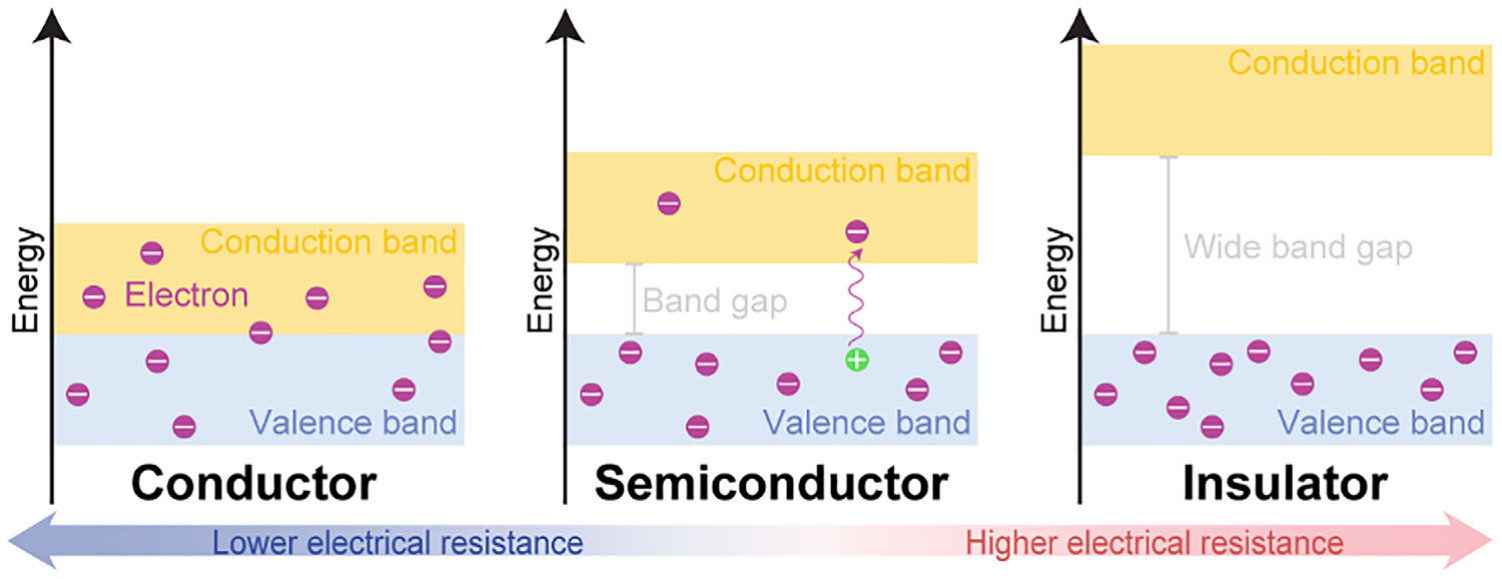
Schematic energy band diagrams of conductors, semiconductors, and insulators.

Neuromorphic bioelectronics require material platforms and operating principles that fundamentally diverge from those of conventional solid‐state electronics [25, 26]. In traditional electronic materials, charge transport is primarily governed by the electronic band structure, where conductors exhibit overlapping valence and conduction bands, semiconductors possess a finite bandgap enabling controllable carrier excitation, and insulators are characterized by wide bandgaps that suppress charge transport altogether (Figure 3). This framework underpins fast, binary, and largely static electronic switching that forms the basis of modern digital electronics [27].

In contrast, biological information processing occurs within soft, hydrated environments where electronic conduction is inseparably coupled with ionic transport and molecular reconfiguration. Neuronal signaling and synaptic plasticity emerge from history‐dependent ionic fluxes, volumetric charge redistribution, and gradual modulation of conductance, rather than from abrupt transitions between discrete electronic states [2, 28]. As a result, conventional materials optimized for purely electronic, interfacial, and binary switching are intrinsically mismatched to the distributed, analog, and time‐dependent nature of biological computation [29].

To bridge this gap, neuromorphic bioelectronic systems require materials that transcend the classical conductor–semiconductor–insulator paradigm by supporting simultaneous ionic and electronic transport throughout the bulk of the active medium. Through this coupled transport, device conductance evolves continuously in response to external stimuli, enabling memory and adaptive behavior to emerge directly from the underlying ionic–electronic dynamics [5, 10, 12]. Throughout this review, we track a recurring set of physical variables, such as conductance state, nonlinearity, retention, volatility, and ionic time constants (e.g., *τ*(ion)/*τ*(RC)), and demonstrate how these parameters are programmed by material design and subsequently manifested as neuromorphic functions in OECT‐based systems [30, 31, 32].

In this section, we therefore examine (i) ionic–electronic coupling materials as the physical basis of neuromorphic function, (ii) temporal and adaptive responses arising from ion dynamics, (iii) OMIECs as representative and tunable material systems, (iv) hybrid and composite strategies that expand the accessible ionic, electronic, and mechanical design space, and (v) material design criteria required for stable, adaptive, and biologically compatible neuromorphic bioelectronics.

### 2.1 Ionic–Electronic Coupling Materials for Neuromorphic Functions

#### 2.1.1 Mixed Ionic–Electronic Conductors

Mixed ionic–electronic conductors (MIECs) represent a foundational class of materials for electrochemically coupled electronic systems, wherein ionic motion and electronic charge transport are intrinsically and volumetrically integrated within a single active material [33, 34]. Unlike conventional semiconductors, which rely exclusively on electronic charge carriers, MIECs enable the concurrent transport of ions and electrons throughout the same material volume [35]. This dual‐transport capability allows external ionic stimuli, such as electrolyte composition, ionic concentration, or applied electrochemical potentials, to directly and continuously modulate the electronic conductivity of the material [10, 36].

**FIGURE 4:**
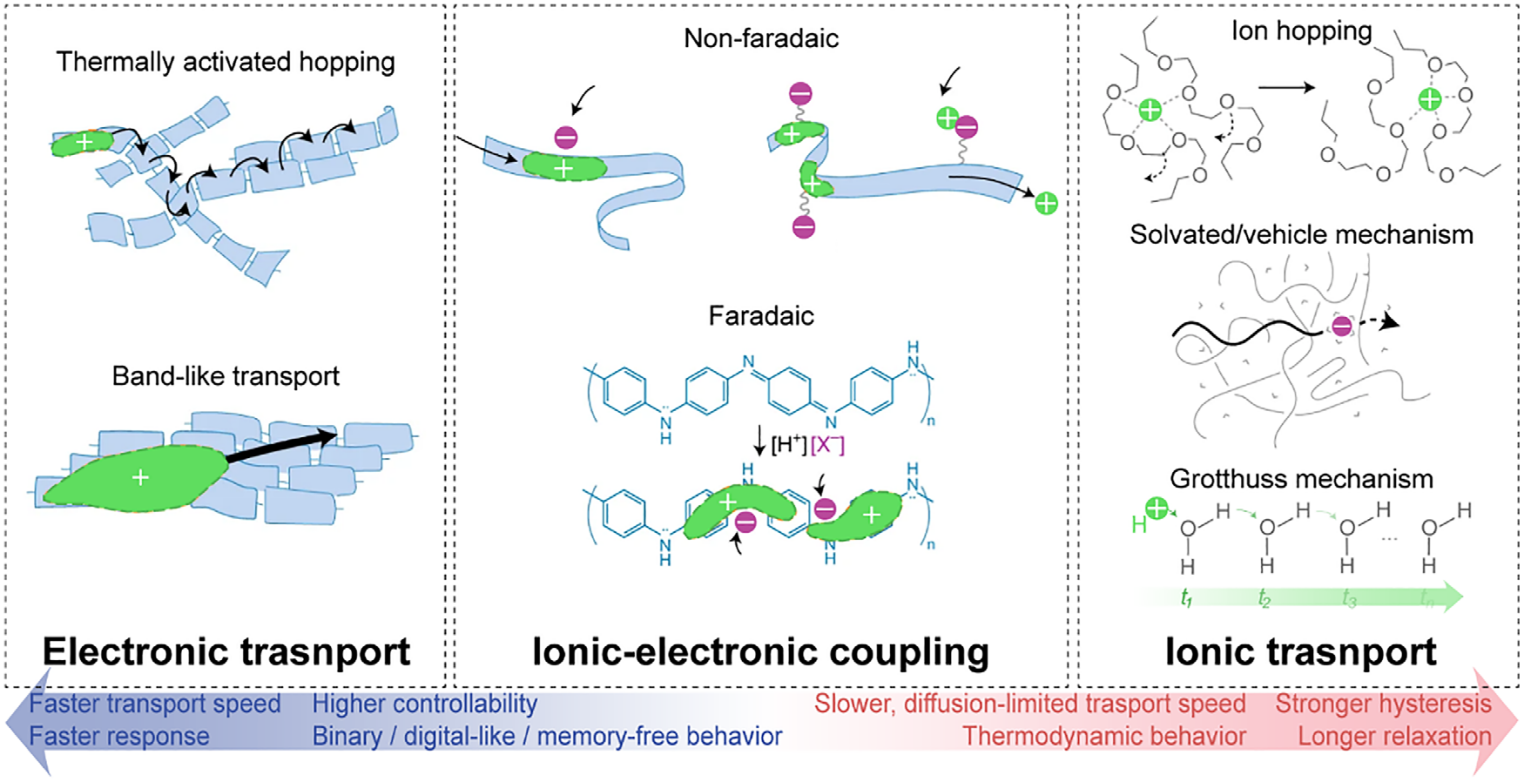
Comparison of electronic transport, ionic–electronic coupling, and ionic transport mechanisms relevant to OMIECs. Reproduced with permission [12]. Copyright 2019, Springer Nature. Reproduced with permission [11]. Copyright 2024, Springer Nature.

Among various MIEC systems, OMIECs, including conducting polymers and conjugated polymers with polar side chains, have emerged as a versatile and broadly applicable materials platform for biointerfaced and electrochemically coupled electronic systems [12, 14, 15, 37, 38, 39, 40, 41]. As schematically illustrated in Figure 4, OMIECs occupy an intermediate transport regime between purely electronic and purely ionic systems. Electronic transport in organic semiconductors typically proceeds either by thermally activated hopping between localized states (common in disordered regions) or by more delocalized, band‐like transport in highly ordered domains. Ionic transport, in contrast, requires mass motion of ions and is commonly described by segmental or coordination site hopping in dry polymer environments, solvated vehicular transport in electrolyte‐swollen films, or, in the case of protons, the Grotthuss mechanism in which charge propagates through a hydrogen bond network. Purely electronic transport is typically rapid and readily controlled, whereas purely ionic transport is often diffusion‐limited and characterized by pronounced hysteresis and long relaxation times. In OMIECs, ionic–electronic coupling enables volumetric interactions between mobile ions and electronic carriers through both non‐Faradaic and Faradaic processes. This coupled transport gives rise to gradual, history‐dependent conductance modulation governed by ionic kinetics, thereby establishing a material‐level physical basis for memory and adaptive behavior [11, 12].

The intrinsic ability of OMIECs to support simultaneous ionic and electronic transport, together with their soft mechanical properties, compatibility with aqueous electrolytes, and chemically tunable ionic accessibility, renders them uniquely suited for interfacing with biological environments and constructing adaptive, bioinspired electronic architecture [42, 43, 44]. Importantly, in many OMIECs, ionic motion is not confined to a surface interface but can penetrate the bulk of the material, enabling true volumetric ionic–electronic interactions [5, 10].

In biological synapses, ionic flux across neuronal membranes induces gradual and reversible changes in synaptic strength, forming the physical foundation of learning and memory. Emulating such continuously evolving, ion‐mediated dynamics in artificial systems requires materials in which electronic conductance is intrinsically modulated by ionic motion [45, 46]. Within this framework, OMIECs, owing to their distinctive mixed ionic–electronic conduction, offer unique advantages for emulating biological synapses. OMIECs enable continuous, analog modulation of conductance rather than abrupt transitions between discrete electronic states. This transport behavior supports analog weight updates, temporal integration, and history‐dependent responses, functionalities that are difficult to achieve using conventional purely electronic materials without substantial circuit‐level complexity [31, 32]. Consequently, OMIECs have emerged as central active materials in neuromorphic bioelectronics for emulating synaptic and neuronal functions [17, 47, 48, 49].

#### 2.1.2 Volumetric Charge Modulation and Electrochemical Gating

A defining operational principle of neuromorphic bioelectronic systems based on OMIECs is volumetric charge modulation enabled by electrochemical gating. In contrast to conventional FETs, where charge accumulation is confined to a narrow interfacial channel at the semiconductor–dielectric interface, electrochemical gating in OMIECs permits ions from an electrolyte to penetrate deeply into the bulk of the active material. This ionic ingress induces reversible redox processes or electrostatic charge compensation throughout the entire channel volume, leading to a volumetric modulation of electronic carrier density rather than a purely interfacial effect [12, 50, 51, 52, 53].

**FIGURE 5:**
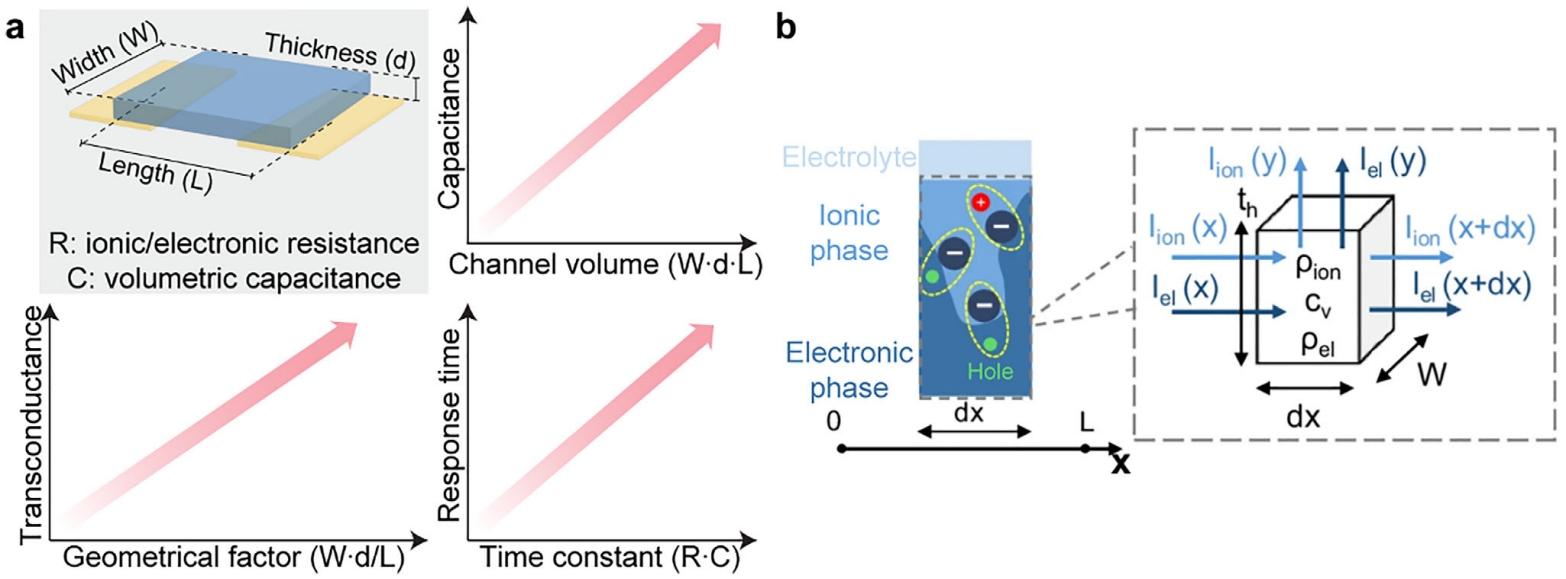
Geometry‐dependent volumetric capacitance and transport in OMIEC thin films. (a) OMIEC channel geometry defined by length (*L*), width (*W*), and thickness (d), which together determine channel volume and volumetric capacitance. (b) Schematic transport model of an infinitesimal OMIEC volume element (d*x*), where ionic and electronic currents flow along the lateral (*x*) and vertical (*y*) directions. Charge accumulation at internal ionic–electronic interfaces is described by volumetric capacitance. Reproduced under the terms of the CC‐BY‐NC‐ND license [43]. Copyright 2019, The Authors, published by Springer Nature.

As illustrated in Figure 5a, the extent of volumetric charging is intrinsically governed by device geometry, defined by the channel length (*L*), width (*W*), and thickness (*d*), which together determine the active channel volume (*W* × *d* × *L*). Because ionic charge is stored throughout the bulk, the effective capacitance of OMIEC devices scales with volume rather than area, giving rise to a volumetric capacitance that can exceed conventional interfacial capacitances by several orders of magnitude. This unusually large capacitance enables substantial modulation of electronic conductivity at low operating voltages, a key advantage for soft, biointerfaced, and low‐power neuromorphic systems [52, 53].

Beyond static charge storage, volumetric electrochemical gating introduces intrinsic temporal dynamics associated with ion transport and relaxation within hydrated polymer matrices. As schematically depicted in Figure 5b, electronic transport along the channel is coupled to ionic displacement and volumetric charging across the film thickness, leading to dissipative and frequency‐dependent signal propagation. Consistent with this framework, a recent study demonstrated that electrical signals in poly(3,4‐ethylenedioxythiophene): poly(styrenesulfonate) (PEDOT:PSS) OMIEC channels exhibit attenuation and phase delay governed by transmission‐line dynamics, with propagation speed and dissipation controlled by the balance between electronic transport and volumetric capacitance [43].

From a neuromorphic perspective, these geometry‐dependent ionic dynamics provide a physical substrate for emulating adaptive synaptic behaviors. Short‐term plasticity (STP) can arise from transient ionic accumulation and rapid relaxation within shallow regions of the bulk, whereas long‐term plasticity (LTP) and memory retention are associated with deeper ion penetration, stabilization, or slow structural relaxation processes within the OMIEC matrix [31]. Repeated electrical stimuli or pulse trains can therefore induce gradual and cumulative conductance modulation, allowing OMIEC‐based devices to encode stimulus history and temporal correlations in a manner analogous to biological synapses. Importantly, the neuromorphic performance envelope of OMIEC systems emerges from the interplay between ion mobility, material morphology, and device geometry. This relationship is captured quantitatively by the OECT transconductance, *g*_m_ = (*Wd*/*L*) × *μ* × *C* ^∗^, where *µ* is the electronic carrier mobility and C* is the volumetric capacitance [10]. Beyond governing amplification, these same geometric parameters also regulate ionic diffusion pathways and relaxation times, thereby defining trade‐offs between speed, stability, reversibility, and energy efficiency [54, 55]. This strong coupling between geometry and function distinguishes OMIEC‐based devices from conventional solid‐state electronics and underscores their unique suitability for bioinspired, time‐dependent information processing.

These geometry–function relationships give rise to scaling considerations that are fundamentally distinct from those governing conventional FETs. Unlike conventional FETs, where interfacial confinement of charge means that a reduction in lateral dimension generally improves both speed and integration density, OECTs store ionic charge throughout the channel volume (*W* × *d* × *L*), and the characteristic ionic time constant scales as *τ*_ion_ ∝ *d* ^2^/*D*_ion_, where *D*_ion_ is the effective ion diffusivity within the OMIEC matrix [10]. Thus, unlike FET scaling, where performance is often improved by reducing device dimensions, OECT scaling is governed by competing dependencies in which smaller dimensions accelerate ion transport but simultaneously reduce volumetric charge storage. Although lateral miniaturization can increase integration density, excessive reduction of channel volume ultimately limits ionic storage capacity and the accessible conductance range, introducing trade‐offs between density, transconductance, and analog resolution [56].

Practically, this volumetric constraint shapes the design space for neuromorphic OECT arrays in ways that differ markedly from conventional CMOS scaling. In FET‐based neuromorphic hardware, aggressive lateral scaling increases transistor density while preserving or improving speed. In OECT‐based systems, the same lateral compression reduces per‐device ionic charge capacity and narrows the conductance modulation window, potentially degrading weight precision and temporal dynamic range precisely when array density increases. Vertical architectures, in which the ionic diffusion length is defined by film thickness rather than lithographic channel length, offer one route to partially decouple footprint from volumetric capacity [57, 58, 59], and electrolyte engineering strategies that enhance *D*_ion_ can potentially improve switching speed while preserving channel volume. These considerations underscore that the path toward dense, high‐fidelity OECT neuromorphic arrays requires co‐optimization of volumetric material properties, device geometry, and electrolyte configuration, a design paradigm with no direct precedent in conventional semiconductor scaling.

### 2.2 Temporal and Adaptive Material Responses

The temporal dynamics of OMIECs originate from finite ion transport kinetics and the coupled structural relaxation of hydrated polymer matrices. Unlike crystalline semiconductors, where electronic carriers respond quasi‐instantaneously, ionic motion in OMIECs is governed by diffusion‐limited transport, viscoelastic matrix deformation, and ion–polymer interactions. These intrinsic processes give rise to rich time‐dependent electrical responses that constitute the physical substrate of neuromorphic and adaptive functionalities [60, 61]. In OMIEC systems, volatility corresponds to the spontaneous relaxation of stimulus‐induced ionic distributions and polymer strain toward equilibrium, whereas non‐volatility requires ion trapping, chemical conversion, or stabilized structural reconfiguration that suppresses back‐diffusion.

**FIGURE 6:**
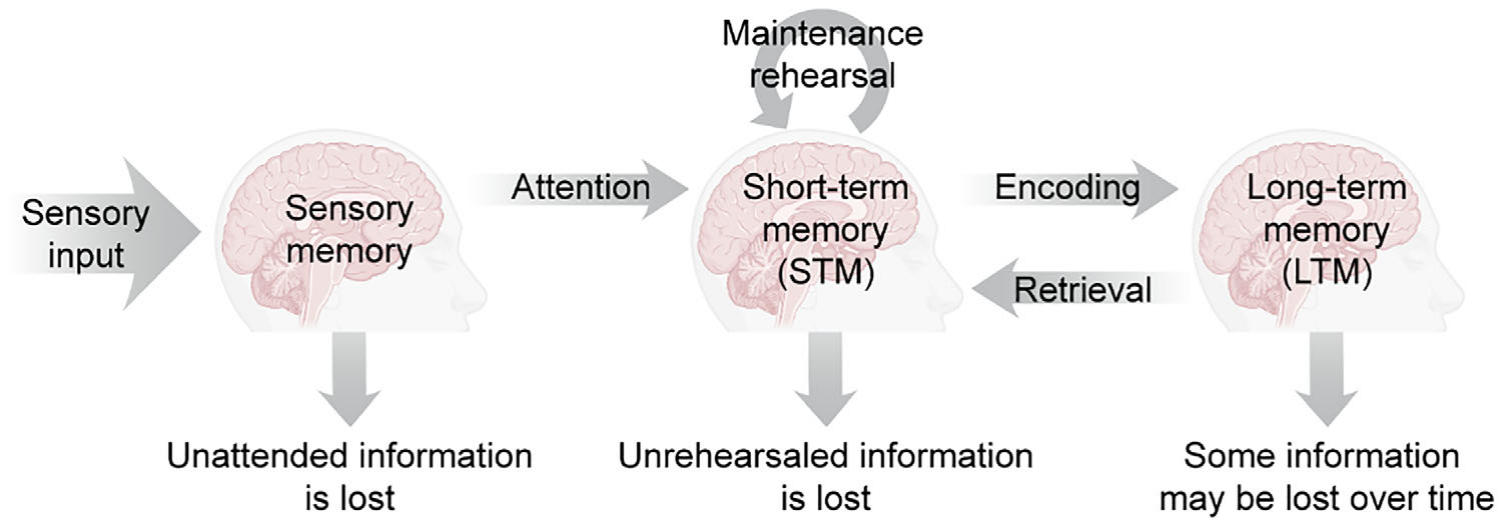
Structure of the memory system.

Crucially, such temporal behaviors are not parasitic artifacts, but functional analogues of biological information processing, in which memory emerges from the selective retention or decay of stimulus‐induced states. As summarized in Figure 6, biological memory is hierarchically organized from sensory memory to short‐term memory (STM) and long‐term memory (LTM), each defined by characteristic stabilization mechanisms and relaxation timescales. Owing to their volumetric ionic–electronic coupling, OMIECs provide a materials platform capable of reproducing multiple memory timescales within a single device architecture [62].

#### 2.2.1 Short‐Term and Long‐Term Memory Effects

**FIGURE 7:**
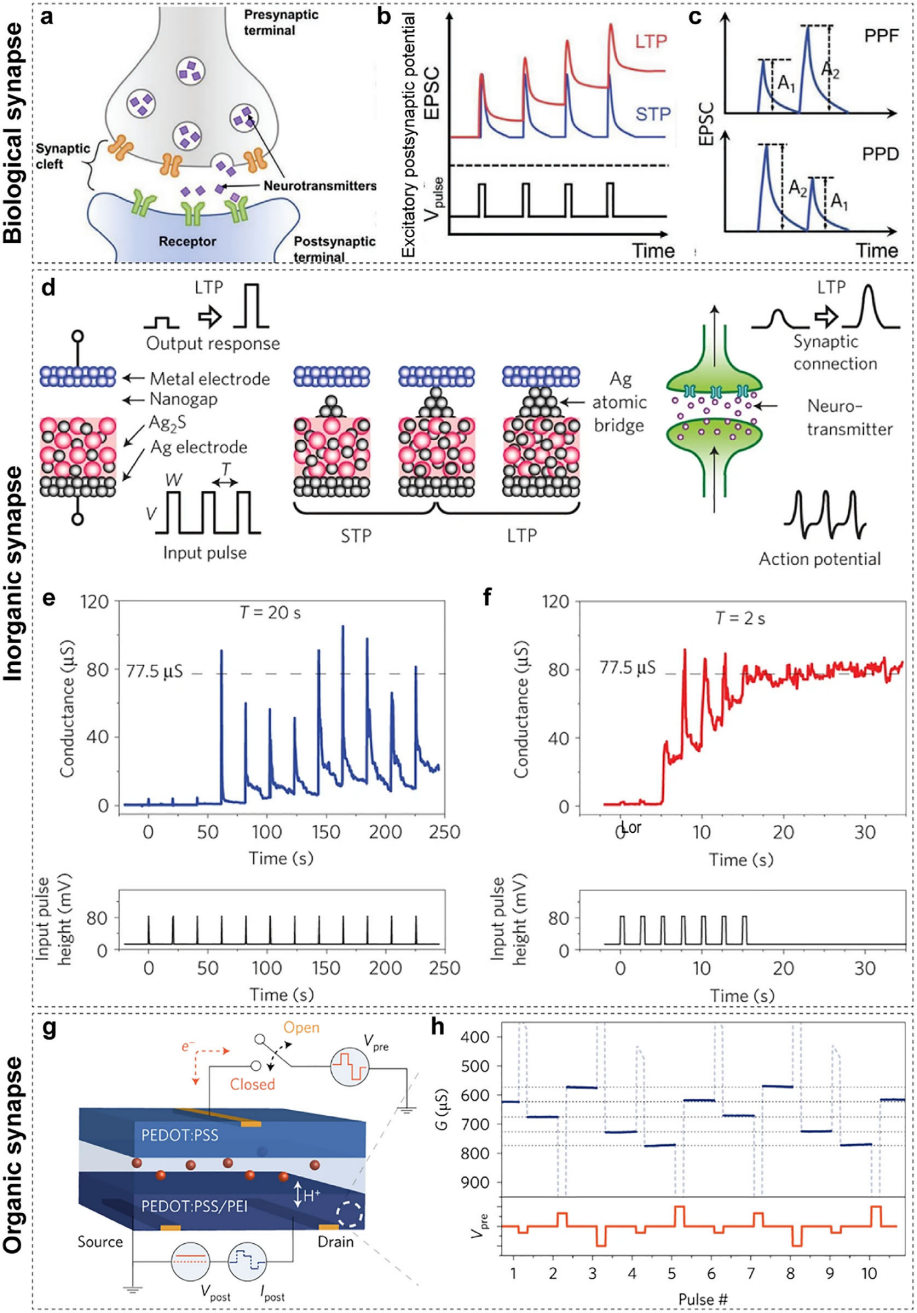
Synaptic plasticity in biology and material emulation. (a) Schematic of neurotransmitter‐mediated synaptic transmission. (b) Example EPSC traces illustrating activity‐dependent transition from STP to LTP. (c) PPF and PPD. A1 and A2 mark the peak EPSC amplitudes for the first and second pulses, and the paired‐pulse ratio is defined as *PPR* = A2/A1. Reproduced with permission [63]. Copyright 2021, Wiley‐VCH. (d) Ag_2_S atomic switch (electrochemical metallization) synapse. Conductance is governed by the formation and relaxation/dissolution of a metallic Ag filament, also described as an Ag atomic bridge, within Ag_2_S, driven by Ag^+^ migration and Ag^+^/Ag^0^ redox. (e) Long inter‐pulse intervals yield volatile conductance relaxation (STP‐like). (f) Short inter‐pulse intervals stabilize filament growth and yield persistent conductance increases (long‐term potentiation‐like). Reproduced with permission [66]. Copyright 2011, Springer Nature. (g) ENODe operates by transporting ions or protons through an electrolyte to modulate the redox state and conductivity of the postsynaptic PEDOT:PSS/PEI film, where the penetration depth into the film thickness determines the characteristic timescales. (h) Pulse‐programmable conductance states in the organic device. Reproduced with permission [32]. Copyright 2017, Springer Nature.

In biological neural systems, memory emerges from activity‐dependent modulation of synaptic efficacy across multiple temporal scales. Neurotransmitter‐mediated signaling within the synaptic cleft dynamically regulates postsynaptic responses (Figure 7a), and repeated stimulation induces distinct synaptic plasticity regimes [63]. STP manifests as transient modulation of the excitatory postsynaptic current (EPSC), whereas LTP represents a sustained enhancement of synaptic strength (Figure 7b). Among the most widely studied forms of STP are paired‐pulse facilitation (PPF) and paired‐pulse depression (PPD), which reflect the dynamic response of synapses to successive stimuli (Figure 7c). In this framework, A1 and A2 denote the peak amplitudes of the first and second EPSC, respectively, and the paired‐pulse ratio is commonly defined as *PPR* = A2/A1. Thus, PPF corresponds to A2 > A1 (*PPR* > 1) while PPD corresponds to A2 < A1 (*PPR* < 1). These responses originate from residual ionic concentrations and vesicle release dynamics within the presynaptic terminal, leading to volatile modulation of synaptic efficacy that decays over characteristic relaxation times [64, 65]. Collectively, these mechanisms establish a temporal hierarchy of biological memory, in which the persistence of synaptic states is intrinsically governed by underlying ionic processes and neurotransmitter kinetics.

Artificial synaptic devices emulate this temporal hierarchy through material‐dependent charge transport and relaxation. In inorganic memristive atomic switch synapses based on Ag_2_S (Figure 7d–f), conductance is governed by electrochemically driven formation and dissolution of a metallic Ag filament, also referred to as an Ag atomic bridge in Figure 7d, within the Ag_2_S solid electrolyte [66]. Under electrical bias, Ag at the active electrode can be oxidized to Ag^+^. These ions migrate through Ag_2_S and are reduced to Ag^0^ near the counter electrode, forming a conductive filament or bridge. When the interpulse interval is long, the metallic filament, also described as an atomic bridge, becomes unstable and relaxes or dissolves between pulses, producing volatile conductance consistent with STP‐like behavior (Figure 7e). In contrast, closely spaced pulses promote cumulative filament growth and stabilization, yielding persistent high conductance states consistent with LTP‐like behavior (Figure 7f). Artificial synaptic devices thus emulate biological memory by tuning the temporal characteristics of electrical pulses, where pulse duration and spacing determine whether conductance changes remain volatile or evolve into stable long‐term states.

In organic electrochemical synaptic devices (Figure 7g,h), memory originates from ionic–electronic coupling in OMIECs. Ion or proton motion modulates the redox state of the channel material and therefore its conductivity [32]. In Figure 7g, the electrolyte transports ions or protons between the presynaptic PEDOT:PSS and the postsynaptic PEDOT:PSS/PEI film. Deeper ion penetration refers to ions or protons diffusing further into the bulk thickness of the postsynaptic PEDOT:PSS/PEI layer, away from the electrolyte interface, rather than remaining confined near the interface. Interfacial ionic accumulation tends to produce short‐lived modulation, while deeper penetration and stabilized redox configurations can lead to longer retention and LTM‐like behavior [32, 67].

Recent developments further extend this framework by improving retention stability, regulating ion–electron coupling, and integrating dual‐modal memory behavior within single devices [68, 69, 70]. Collectively, Figure 7 illustrates a unifying principle in which memory in both biological and artificial systems is closely associated with charge carrier or ionic state dynamics operating over distinct relaxation timescales. Engineering these dynamics enables the translation of biological plasticity rules into programmable material states, thereby bridging synaptic physics and neuromorphic computation.

#### 2.2.2 Hysteresis, Relaxation, and History‐Dependent Dynamics

**FIGURE 8:**
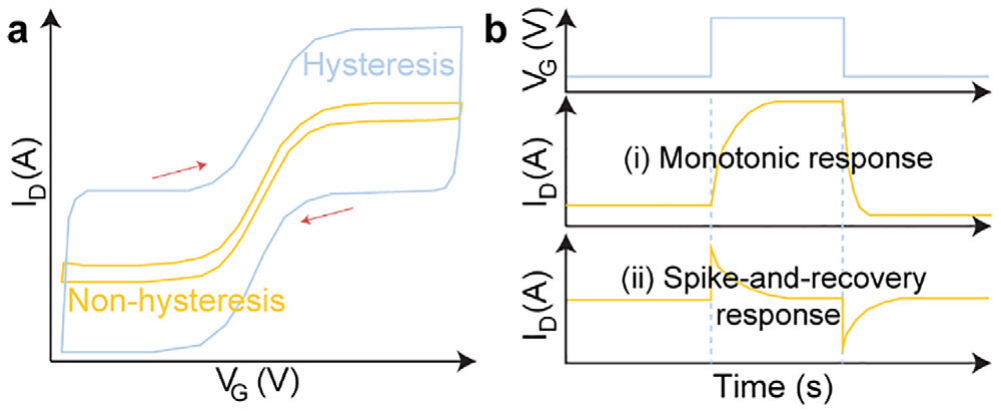
Hysteretic behavior and transient dynamics in OMIECs. (a) Transfer characteristics showing hysteresis in the drain current–gate voltage response. (b) Transient drain current responses to a gate voltage pulse: (i) monotonic relaxation, where the drain current approaches steady state following electrochemical charging, and (ii) spike‐and‐recovery dynamics, characterized by a transient current overshoot followed by relaxation.

Electrochemical doping in OMIECs inherently produces hysteresis in the drain current‐gate voltage characteristics (Figure 8a), reflecting the history‐dependent nature of ionic–electronic coupling, where channel conductance depends on both the instantaneous stimulus and preceding ionic states [71, 72].

Following a change in gate bias, the device approaches equilibrium through relaxation processes governed by ion diffusion, steric constraints of the polymer network, and viscoelastic responses of the host matrix. The transient current therefore exhibits either monotonic relaxation associated with ion‐limited volumetric charging or a spike‐and‐recovery response when electronic transport becomes rate‐limiting (Figure 8b) [73].

Accordingly, OMIECs integrate successive stimuli and encode temporal correlations between inputs, giving rise to intrinsic temporal memory and adaptive signal processing behaviors such as temporal integration and filtering [74, 75, 76]. At a more fundamental level, operando grazing‐incidence X‐ray photon correlation spectroscopy measurements have shown that hysteresis and relaxation originate from coupled charge‐carrier dynamics and mesoscale structural evolution, exhibiting long‐lived non‐equilibrium strain and reversible structural rearrangements beyond simple ion diffusion processes [77].

#### 2.2.3 Reversible Strategies for Volatile–Nonvolatile Tuning

Non‐volatility in OMIEC‐based devices is often associated with ion trapping or structural reconfiguration that suppresses back‐diffusion. For adaptive neuromorphic systems, however, it is often desirable to reversibly access both volatile and non‐volatile regimes within the same device platform. Recent studies show that such reversible control can be implemented at distinct levels of the device stack, channel architecture, stimulation protocol, and molecular trap design without introducing separate materials or destructive processing.

At the architecture level, phase‐separated channel morphologies enable bias‐selectable volatility. In the vertical traverse cv‐OECT (Section 4.2.1), low gate bias dopes only the amorphous fraction, producing rapidly relaxing, volatile behavior, while bias above a threshold drives ions into crystalline domains, yielding stable multi‐level non‐volatile states retained for over 20 000 s [78]. Both regimes are reversibly accessed within the same device, so gate‐voltage amplitude alone serves as a non‐destructive volatile/non‐volatile switch.

At the stimulation‐protocol level, the temporal structure of gate pulses can determine whether conductance modulation remains transient or evolves into a persistent state within a single ion‐trapping channel. A PEDOT:Tosylate/PTHF channel exhibits transient, short‐term facilitation under brief pulse trains, while increasing the number of presynaptic pulses, analogous to rehearsal, progressively shifts the response toward a persistent, LTM level retained for over 200 min within a ± 0.8 V write/erase window [79]. Within this same bias window, the conductance can be reversibly programmed to different levels by pulses of opposite polarity, without chemical modification of the channel. Thus, stimulus history, rather than chemical modification, can govern whether a given response behaves as transient or persistent for a fixed material.

At the molecular design level, deep ion traps can be engineered through purely electrostatic (non‐Faradaic) interactions rather than redox‐driven doping. A zwitterionic crosslinker in the channel polymer combines a repulsive sulfonate barrier that delays ion injection with a cationic ammonium site, forming a ∼2.03 eV electrostatic trap that stabilizes the doped state. This design yields a memory window of 8.65 V and an on/off ratio of ∼10^6^, with Z‐FPA–incorporated devices retaining 89.6% of their initial conductance after 20 000 switching pulses, and operando structural analysis confirming reversible lamellar reorganization rather than chemical degradation [80].

Taken together, volatile and non‐volatile regimes need not correspond to distinct materials or irreversible states, but can be reversibly accessed via architectural phase separation, stimulus‐protocol design, or electrostatic molecular traps. Combining these levers, for example, an electrostatically stabilized channel within a threshold‐gated architecture driven by rehearsal‐like pulse protocols, offers a route to OECT platforms where working‐memory and long‐term‐memory functions coexist within a single device and can be dynamically reallocated without progressive degradation, directly relevant to adaptive neuromorphic systems that must continually reconfigure their memory hierarchy.

### 2.3 OMIECs as Representative Material Systems

OMIECs support coupled ionic and electronic transport, and their neuromorphic behavior becomes most evident when implemented in device architectures that exploit volumetric electrochemical gating. OECTs represent the most widely adopted platform for translating OMIEC material properties into functional neuromorphic devices [10, 81].

**FIGURE 9:**
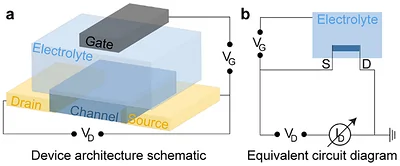
Device‐level representation of an OECT. (a) Device architecture showing electrolyte‐gated modulation. (b) Equivalent circuit.

In OECTs, an electrolyte gate modulates the conductivity of the OMIEC channel via ion penetration into the bulk of the material, producing volumetric doping and dedoping. This operation enables low‐voltage operation, large transconductance, and time‐dependent responses governed by ion transport and relaxation. As illustrated in Figure 9a, ions are injected from the electrolyte into the channel between source and drain, while Figure 9b shows the equivalent circuit describing gate‐controlled modulation of the drain current. As a result of their intrinsic ionic–electronic coupling and history‐dependent conductance dynamics, OECTs have been widely adopted both as bioelectronic interfaces [13, 82, 83] and as artificial synapses and neurons in neuromorphic systems [57, 84, 85].

#### 2.3.1 PEDOT:PSS and Related Conducting Polymers

**FIGURE 10:**
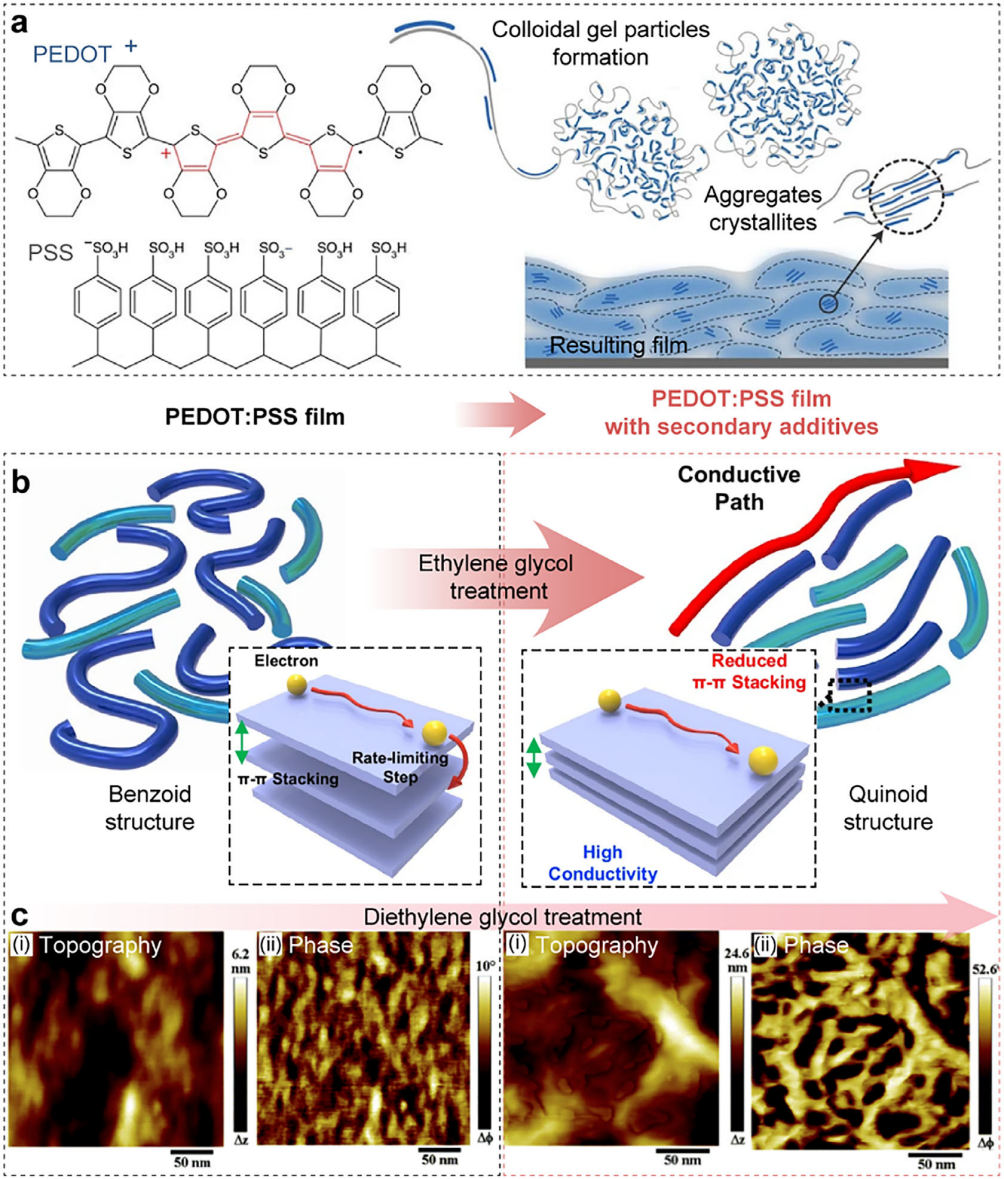
Secondary‐dopant‐induced structural and electrical evolution of PEDOT:PSS films. (a) Pristine PEDOT:PSS film. Reproduced under terms of the CC‐BY license [56]. Copyright 2026, The Authors, published by Springer Nature. (b) Schematic structural reorganization before and after ethylene glycol treatment. Reproduced under the terms of the CC‐BY license [90]. Copyright 2025, The Authors, published by Springer Nature. (c) AFM images before and after diethylene glycol treatment. Reproduced with permission [87]. Copyright 2006, American Chemical Society (ACS).

PEDOT:PSS is the most widely used OMIEC due to its high conductivity, aqueous stability, and solution processability. In this biphasic system, the PSS‐rich phase acts as an ion‐conducting polyelectrolyte matrix, while the PEDOT‐rich domains form a percolating electronic pathway, enabling efficient volumetric ionic–electronic coupling (Figure 10a) [56, 86, 87].

Performance is strongly governed by nanoscale phase connectivity. Secondary dopants such as ethylene glycol weaken Coulombic PEDOT:PSS interactions and induce PEDOT chain aggregation, forming more continuous conductive pathways (Figure 10b). This reorganization increases structural ordering and phase separation, as evidenced by the continuous pathways observed in AFM morphology after treatment (Figure 10c), which facilitates more uniform ion penetration and dedoping throughout the bulk. Consequently, secondary‐doped PEDOT:PSS exhibits increased volumetric capacitance, improved carrier transport, and more stable analog conductance modulation, establishing it as the benchmark channel material for OECT‐based neuromorphic devices [88, 89, 90].

#### 2.3.2 Glycolated and Donor–Acceptor Polymers

**FIGURE 11:**
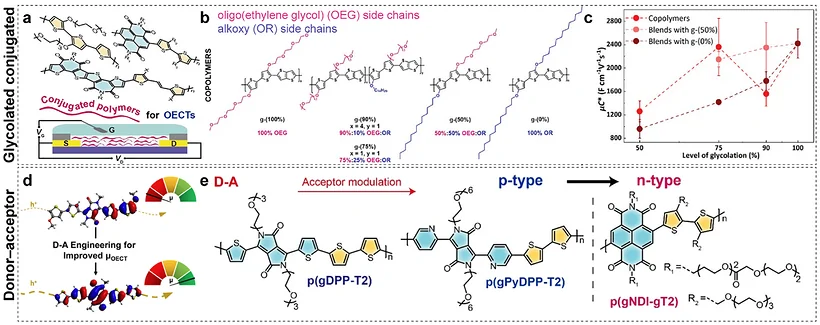
Molecular design strategies for glycolated conjugated polymers in OECTs. (a) Schematic of glycolated conjugated polymer channels. Reproduced with permission [96]. Copyright 2025, Wiley‐VCH. (b) OEG versus OR side chains. (c) Mobility–capacitance product (µC*) versus OEG: OR side‐chain ratio for OECT channel materials. g‐(*x*%) represents the percentage of glycolated side chains in the polymer. µC* (*µ* × C*) reflects the efficiency of mixed ionic–electronic conduction. Reproduced under the terms of the CC‐BY license [95]. Copyright 2025, The Authors, published by Wiley‐VCH. (d) Schematic of donor–acceptor backbone engineering. Reproduced with permission [100]. Copyright 2020, Wiley‐VCH. (e) Polarity tuning from p‐type to n‐type OMIECs. Reproduced with permission [96]. Copyright 2025, Wiley‐VCH.

While PEDOT:PSS provides efficient mixed conduction, its phase‐separated morphology and intrinsically p‐type transport limit complementary circuit architectures. Glycolated conjugated polymers overcome this constraint by covalently incorporating oligo (ethylene glycol) (OEG) side chains directly into the semiconducting backbone, introducing ion‐coordination sites within the bulk semiconductor rather than relying on an external polyelectrolyte phase (Figure 11a,b). This molecular design promotes homogeneous ion penetration and enables volumetric electrochemical doping governed primarily by polymer energetics rather than phase morphology. Consequently, the degree of glycolation directly influences swelling, ionic accessibility, and OECT performance. A key figure of merit for OECT channel materials is the mobility–capacitance product (µC*), defined as the product of electronic carrier mobility (*µ*) and volumetric capacitance (C*), which reflects the ability of the material to simultaneously transport electronic charges and store compensating ionic charges during electrochemical gating. Figure 11c compares the OECT performance of copolymer and blended channel materials with different OEG‐to‐alkoxy (OR) side‐chain ratios, where g‐(*x*%) denotes the fraction of glycolated side chains in the polymer. The copolymer series exhibits a non‐monotonic µC* trend, likely influenced by variations in molecular weight and synthesis‐related factors. In contrast, the blended systems display clearer and more predictable behavior: blends containing g‐(50%) maintain µC* values comparable to the fully glycolated polymer, whereas blends incorporating the hydrophobic g‐(0%) polymer show a gradual decrease in µC* as the alkoxy fraction increases. Overall, polymer blending emerges as an effective strategy to systematically tune OECT performance while preserving high µC* [91, 92, 93, 94, 95, 96].

In parallel, donor–acceptor backbone engineering tunes the frontier orbital energies through acceptor modulation, stabilizing electron transport in aqueous electrolytes and enabling n‐type OMIECs alongside conventional p‐type materials (Figure 11d,e). The coexistence of both carrier polarities supports complementary OECT operation, reducing static power consumption and enabling more biologically realistic signal‐processing dynamics [97, 98, 99, 100].

#### 2.3.3 Hydrogel‐Based and Soft Semiconducting Materials

Hydrogel‐based semiconducting materials constitute the most mechanically compliant class of OMIECs, closely matching the hydrated and ionic environment of biological tissue. Their high water content (typically >90%) enables rapid ion transport through porous networks, while embedded conducting polymer or conjugated domains preserve electronic conduction, supporting efficient mixed ionic–electronic operation in soft biointerfaces [7, 16, 101].

This combination of high ionic mobility and continuous electronic percolation enables rapid electrochemical modulation at low voltages with tissue‐like mechanics, making these materials attractive for neuromorphic synaptic transistors and soft neural interfaces. Embedding semiconducting polymer networks within hydrated hydrogel matrices yields devices with strong ionic–electronic coupling and mechanical compliance that can reliably transduce ionic signals with high fidelity and support low‐power signal amplification.

**FIGURE 12:**
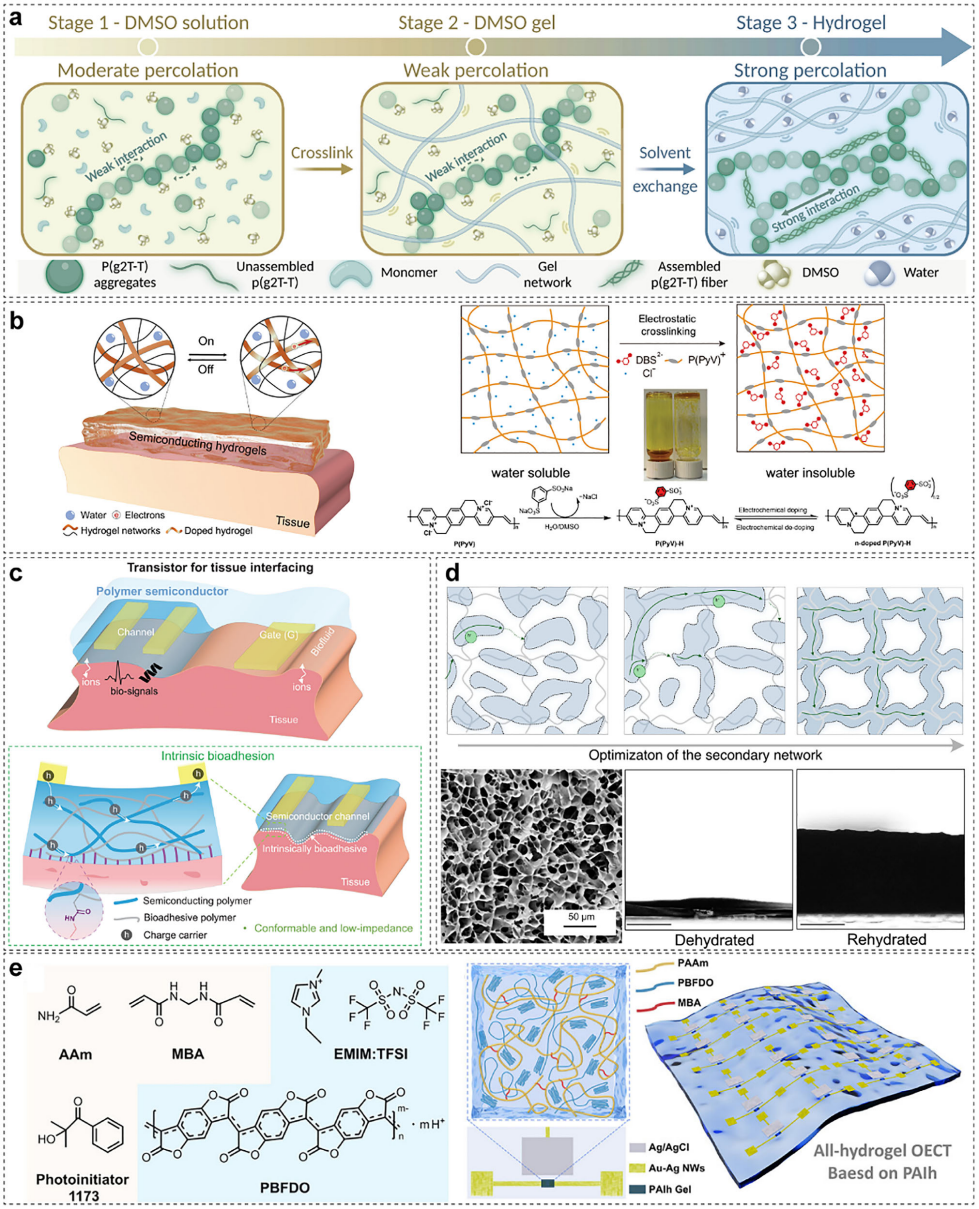
Semiconducting hydrogel materials and device architectures. (a) Ultrasoft mixed‐conducting hydrogel enabling volumetric electrochemical doping at low voltage. Reproduced with permission [102]. Copyright 2024, The American Association for the Advancement of Science (AAAS). (b) n‐type semiconducting hydrogel supporting complementary ionic–electronic operation. Reproduced with permission [103]. Copyright 2024, AAAS. (c) Bioadhesive hydrogel transistor providing stable tissue–device electrical coupling. Reproduced with permission [104]. Copyright 2023, AAAS. (d) Three‐dimensional hydrogel transistor architecture enabling volumetric signal modulation. Reproduced with permission [105]. Copyright 2025, AAAS. (e) Interconnected PBFDO networks in a soft PAAm matrix form a photo‐patternable n‐type semiconducting hydrogel for all‐hydrogel OECTs. Reproduced under the terms of the CC‐BY license [106]. Copyright 2025, The Authors, published by Wiley‐VCH.

Ultrasoft conjugated‐network hydrogels form continuous mixed‐conducting pathways within hydrated matrices, enabling volumetric electrochemical doping at biologically compatible voltages while maintaining tissue‐level mechanics (Figure 12a) [102]. Chemically stabilized n‐type semiconducting hydrogels further introduce electron‐transporting mixed conductors, allowing complementary operation beyond conventional p‐type OMIECs (Figure 12b) [103]. At the interface level, bioadhesive semiconducting polymers create conformal electrical contact with wet tissues, reducing interfacial impedance and preserving signal fidelity under deformation (Figure 12c) [104]. Moving beyond planar devices, three‐dimensional hydrogel transistor architectures exploit volumetric charge injection and spatial ion–electron coupling, enabling distributed signal modulation throughout the material volume (Figure 12d) [105]. Finally, ionic‐liquid–induced phase separation forms interconnected conductive domains that enable a photo‐patternable n‐type depletion‐mode hydrogel transistor capable of stable signal amplification and physiological monitoring (Figure 12e) [106]. Together, these advances demonstrate a progression from mechanically matched mixed conductors to polarity‐complementary materials, biointegrated interfaces, and volumetric device architectures, establishing semiconducting hydrogels as a foundation for neuromorphic bioelectronics.

### 2.4 Hybrid and Composite Material Systems

While single‐component OMIECs effectively support coupled ionic–electronic transport, their performance is often constrained by trade‐offs among mechanical compliance, ionic accessibility, electronic mobility, and long‐term operational stability. Hybrid and composite material strategies address these limitations by spatially and functionally decoupling ionic and electronic transport pathways while introducing additional dynamic behaviors relevant to neuromorphic operation [5, 15, 107].

**FIGURE 13:**
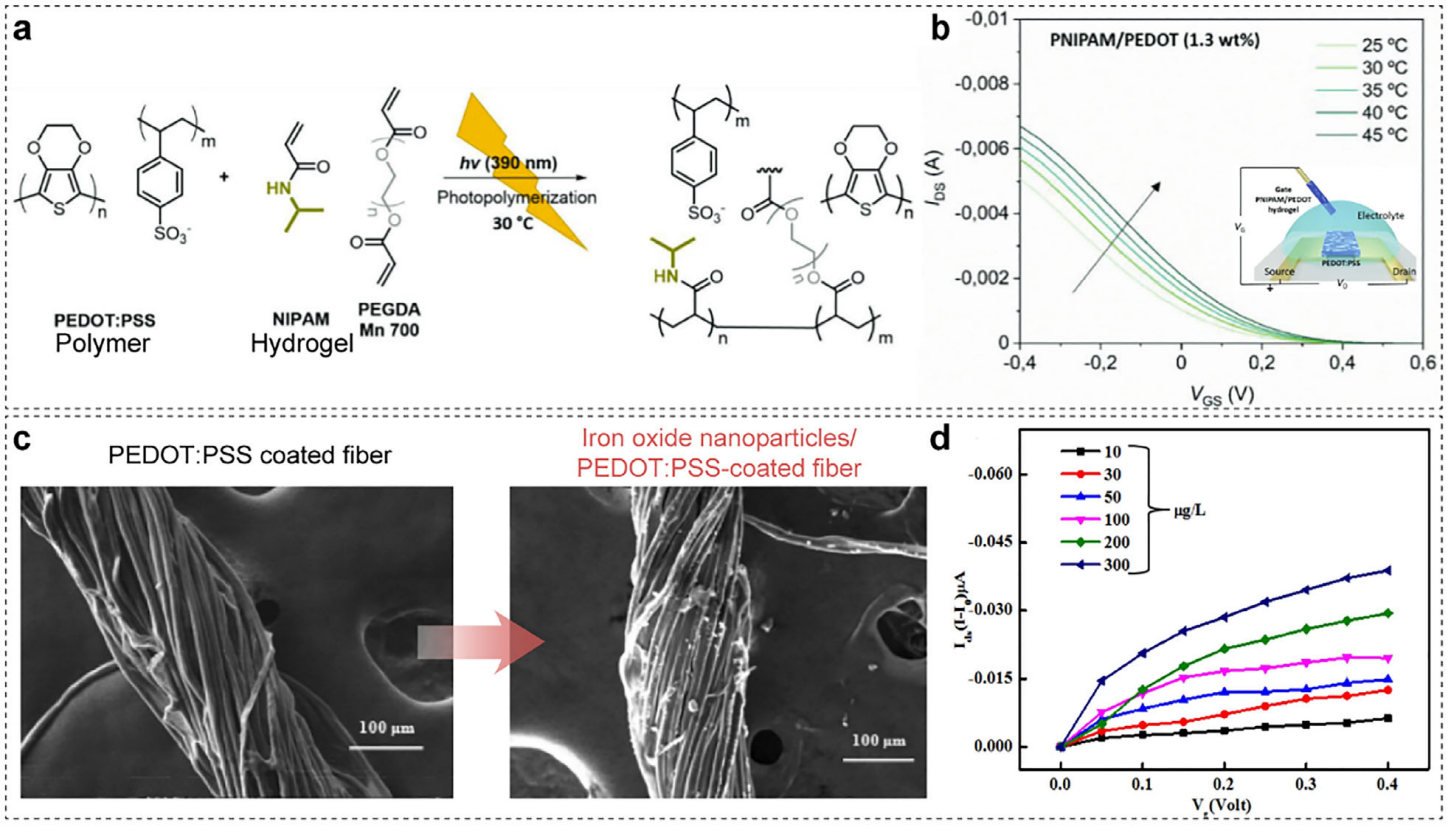
Hybrid composite material systems. (a) Formation of an interpenetrating PEDOT:PSS–hydrogel network for hybrid hydrogel‐gated OECTs. (b) Temperature‐dependent transfer characteristics of the PNIPAM/PEDOT hybrid system. Reproduced under the terms of the CC‐BY license [108]. Copyright 2024, The Authors, published by Wiley‐VCH. (c) Scanning electron microscopy images of PEDOT:PSS‐coated fibers before and after nanoparticle incorporation. (d) Output characteristics of an iron oxide nanoparticle/PEDOT:PSS nanocomposite‐modified cotton fiber‐based OECT showing current modulation at different arsenic concentrations. Reproduced with permission [110]. Copyright 2022, Springer Nature.

In polymer–hydrogel hybrid systems, PEDOT:PSS integrated with hydrated polymer networks such as poly(N‐isopropylacrylamide) (PNIPAM) forms a soft, ion‐permeable gating interface while maintaining efficient electronic conduction within the OMIEC channel. For example, incorporating a PNIPAM/PEDOT hydrogel as the gate electrode of an OECT enables miniaturized bioelectronic devices (Figure 13a) [108, 109]. The corresponding temperature‐dependent transfer characteristics demonstrate thermally tunable transistor operation, exhibiting reversible thermoresponsive behavior between 25°C and 45°C with a temperature sensitivity of approximately 0.05°C^−^^1^ (Figure 13b).

Polymer–nanomaterial composites introduce controlled electronic heterogeneity within the OMIEC channel. For instance, the incorporation of iron‐oxide nanoparticles into PEDOT:PSS‐coated fibers modifies electronic conduction pathways while preserving ionic penetration, thereby perturbing local doping dynamics and relaxation kinetics and inducing nonlinear conductance evolution (Figure 13c). Such nanocomposite architectures enable tunable analog current modulation and broaden transfer characteristics in fiber‐based OECT devices (Figure 13d) [110].

More recently, interpenetrating all‐gel OMIEC architectures based on PEDOT:PSS/polyacrylamide double‐network hydrogels have been reported, in which co‐continuous ionic and electronic transport pathways are maintained during mechanical deformation and extended electrochemical operation [111]. This uniform volumetric doping mechanism enhances operational stability while preserving repeatable history‐dependent conductance behavior during prolonged device operation. The material systems discussed throughout this section should be viewed as complementary components of a broader neuromorphic design space rather than competing alternatives. PEDOT:PSS remains the most mature and widely adopted OMIEC owing to its high volumetric capacitance, facile processing, and robust low‐voltage operation, making it particularly suitable for biointerfaced sensing and adaptive signal processing.

Glycolated polymers offer improved control over ionic transport and electrochemical stability, enabling more tunable neuromorphic responses. Donor–acceptor OMIECs further expand the design space through stable n‐type operation and complementary circuit functionality, which are expected to become increasingly important for energy‐efficient neuromorphic architectures. In contrast, hydrogel semiconductors prioritize mechanical compliance and tissue‐like properties, making them attractive for long‐term biointegration despite remaining challenges in stability and manufacturing reproducibility. Viewed collectively, these materials reveal a recurring structure–property–function relationship in neuromorphic OMIECs: materials with higher ionic accessibility generally facilitate efficient volumetric gating and low‐voltage operation but may sacrifice retention and long‐term stability, whereas materials that stabilize ionic distributions often improve memory performance at the expense of switching speed or fabrication complexity. At present, no OMIEC material simultaneously combines high µC*, complementary carrier polarity, tissue‐like mechanics, long‐term operational stability, and scalable manufacturability. Consequently, optimal device performance will likely emerge from application‐specific material selection or co‐integration of complementary material classes within a single device stack, guided by the structure–property–function principles established here. To provide a quantitative benchmarking perspective across representative OMIEC material classes, Table 1 summarizes key material‐level performance metrics relevant to neuromorphic OECT operation, including carrier polarity, electrolyte system, mobility–capacitance product (µC*), volumetric capacitance (C*), response time, and demonstrated neuromorphic functions. The comparison reveals the distinct trade‐offs among ionic accessibility, electrochemical stability, charge‐transport efficiency, and temporal dynamics that govern neuromorphic performance across different OMIEC families.

**TABLE 1:** Representative OMIEC channel materials for OECT‐based neuromorphic bioelectronics: Key performance parameters.

Material class	Channel material	Carrier polarity	Electrolyte (aq.)	Mobility (cm^2^/V/s)	µC* (F/cm/V/s)	C* (F/cm^3^)	Response time	Neuromorphic function	Refs.
PEDOT:PSS	PEDOT:PSS	p‐type	0.1 m NaCl	N/R	∼48	∼40	N/R	STP, LTP, PPF	[56, 86]
Glycolated polythiophene	p(g2T2‐g4T2) / p(g1T2‐g5T2)	p‐type	0.1 m NaCl	1.72/2.61	522/496	187/133	N/R	STP, LTP; tunable relaxation timescale	[94]
Glycolated D‐A copolymer (p‐type)	PDPP‐4EG	p‐type	0.1 m NaCl	6.49 ± 0.60	702	108	39 ms	STP, LTP	[92]
Ambipolar D‐A copolymer	P(bgTBDOPV‐EDOT)	p/n‐type	0.1 m NaCl	1.57(p)/0.45(n)	235(p)/98(n)	150(p)/218(n)	0.48 ms (p), 0.41 ms (n)	Complementary inverter	[97]
n‐type D‐A polythiophene	o‐CNgTVT‐2FT	n‐type	0.1 m NaCl	0.517	100	193	2.86 ms	n‐type stable accumulation; complementary circuit	[99]
Hydrogel semiconductor	p(g2T‐T)/PAAc hydro‐SC	p‐type	0.1 m NaCl	1.43	N/R	N/R	N/R	Hydrogel‐based OECT; volumetric biosensing; photomodulation	[102]

## 3 Manufacturing Technologies and Device Architectures for Neuromorphic Bioelectronics

In the previous section, we discussed the key properties and functionalities of materials required for the efficient operation of neuromorphic bioelectronics. While ionic–electronic coupling and the temporal dynamics of the material constitute the origin of neuromorphic functions, appropriate manufacturing technologies are necessary to realize these functions stably [12, 31, 32]. Even when the same material is employed, different processing conditions can lead to distinct operating mechanisms, and device geometry, dimensionality, and electrolyte accessibility further play decisive roles [10, 56, 112, 113, 114].

For instance, thin planar channels exhibit fast transistor‐like responses governed by interfacial capacitive gating, whereas thick porous channels display synaptic plasticity and memory retention arising from volumetric electrochemical doping [32, 53, 115, 116]. In both cases, the observed device dynamics originate from the extent of ionic penetration into the channel. Similarly, when electrolyte interaction is confined to the interface, conductance modulation is typically volatile, whereas deeper ionic penetration into the channel bulk promotes long‐term stabilization of the conductance state [32, 117]. These observations highlight that intrinsic material properties alone do not fully determine device behavior. Instead, neuromorphic functionality emerges from the interplay between material properties and device architecture. Consequently, identical material systems can exhibit distinct neuromorphic behaviors, ranging from interfacial capacitive gating to volumetric electrochemical modulation, depending on fabrication strategies and architectural design [72].

As a result, not only materials but also manufacturing technologies and device architectures are critical factors governing the operating principles of neuromorphic bioelectronics. In this section, we summarize conventional microfabrication strategies and emerging manufacturing approaches, and discuss how fabrication methods correlate with device architecture, electrolyte organization, and the resulting modes of operation.

### 3.1 Conventional Microfabrication Approaches

OECTs operate based on volumetric ionic–electronic coupling within the channel, where ion penetration modulates the conductivity of the entire bulk. As a result, device behavior depends not only on the intrinsic characteristics of the channel material but also on the fabrication strategy that determines geometry and interfacial accessibility. The fabrication of OECTs involves multiple processing steps. Over the past decade, OECT microfabrication has advanced significantly through the adoption of conventional silicon‐based techniques, such as photolithography and vacuum deposition [118, 119]. However, these techniques were originally optimized for interfacial electronic devices and can introduce challenges when implemented on flexible substrates [22]. Furthermore, solvent exposure and plasma treatment during processing may modify polymer morphology, and the fabrication procedures are typically associated with high cost and substantial process complexity [120]. Therefore, a detailed understanding of the manufacturing processes is essential for interpreting device performance. The following section presents recent developments in conventional microfabrication technologies.

#### 3.1.1 Lithography‐Based Processing

**FIGURE 14:**
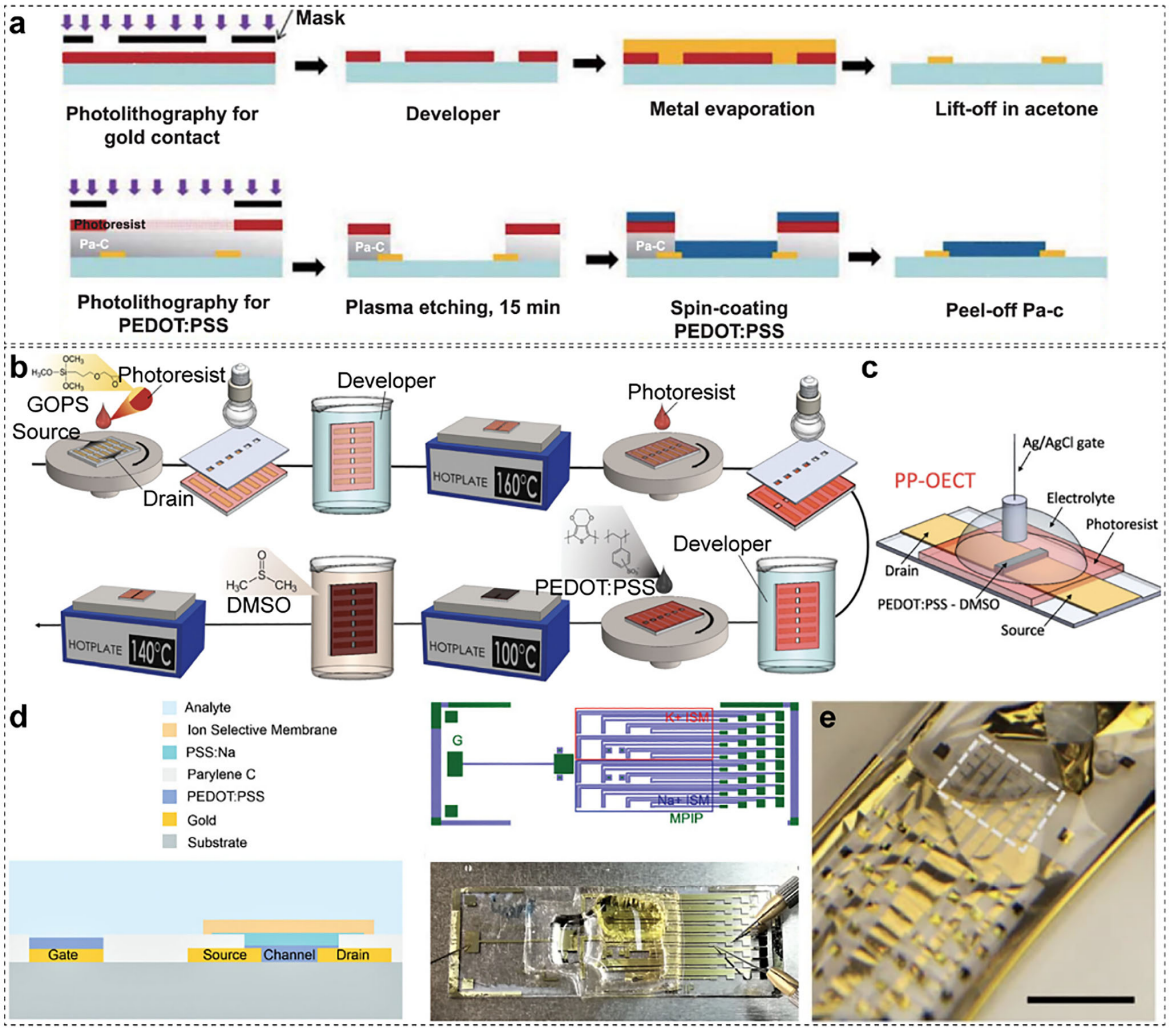
Photolithography‐based microfabrication strategies. (a) Conventional photolithography process used for OECT fabrication. Reproduced under the terms of the CC‐BY license [24]. Copyright 2023, The Authors, published by IOP Publishing. (b) Process flow for photolithographic patterning of PEDOT:PSS OECTs using two photoresist layers. (c) Schematic structure of a photolithographically patterned PEDOT:PSS OECT (PP‐OECT). Reproduced under the terms of the CC‐BY license [120]. Copyright 2025, The Authors, published by Wiley‐VCH. (d) Cross‐sectional schematic of the ion‐selective membrane OECT (ISM‐OECT) device structure. Top‐view layout of the device. Optical micrograph of the fabricated device. Reproduced under the terms of the CC‐BY‐NC‐ND license [122]. Copyright 2021, The Authors, published by Wiley‐VCH. (e) Photograph of flexible OECT arrays fabricated using parylene peel‐off lithography. Reproduced under terms of the CC‐BY‐NC‐ND license [52]. Copyright 2013, The Authors, published by Springer Nature.

Lithography, a representative microfabrication technique used to precisely define patterns on substrates in Figure 14a, serves as the foundation of modern electronic device manufacturing. Among various lithographic approaches, photolithography has become the central processing method [21, 22, 24]. Owing to its high resolution and reliability, this technique is widely utilized for fabricating dense arrays and complex micro‐ and nanoscale architectures [121]. Because ionic–electronic coupling scales with channel thickness and lateral dimensions, precise pattern definition in OECTs is essential for controlling gain, switching dynamics, and energy dissipation. This dimensional sensitivity becomes increasingly critical in multichannel arrays and nanoscale bioelectronic platforms.

Conventional photolithography requires expensive equipment and complex processing and is difficult to apply to flexible substrates or curved devices. In addition, OECT channel materials are easily damaged by photoresist solvents, necessitating special solvents and protective layers [22, 120]. To address these challenges, numerous studies have explored modified microfabrication strategies to adapt photolithographic processing to OECTs. For example, a dual‐crosslinker photoresist strategy has been reported to precisely pattern PEDOT:PSS OECTs (Figure 14b,c), suppressing Faradaic reactions and improving device reproducibility [120]. Moreover, parylene peel‐off photolithography has been employed to enable patterning while protecting the semiconducting polymer from harsh processing conditions (Figure 14d), enabling fabrication on flexible substrates (Figure 14e) [52, 122]. Collectively, photolithography has continuously evolved for organic material systems and remains a central manufacturing strategy for high‐density bioelectronics and neuromorphic biointerfaces. Importantly, precise dimensional control arising from photolithography directly influences key device properties, such as switching dynamics and energy consumption. This device‐level optimization and uniformity are essential for large‐scale neuromorphic arrays and learning accuracy.

#### 3.1.2 Deposition Processing

After pattern definition, other components are typically formed using deposition processes such as spin coating and vapor deposition. Spin coating, a low‐cost, highly uniform, and high‐throughput thin‐film deposition technique, is widely used for forming OECT channels [123]. However, because the entire substrate is coated, additional patterning steps are required to define the active regions accurately, and a significant amount of material is wasted during processing. On the other hand, vapor deposition processes such as chemical vapor deposition (CVD) and physical vapor deposition (PVD) are widely utilized for the formation of high‐quality thin films [22]. CVD enables precise thickness control while producing highly crystalline films. In contrast, PVD relies on the physical transfer of material, allowing for the formation of dense metal and functional multilayer structures. However, both techniques typically require expensive vacuum equipment and cleanroom infrastructure. In addition, their relatively high thermal and energy requirements limit compatibility with thermally sensitive flexible substrates. Therefore, despite their advantages, equipment dependence and process complexity remain important considerations in conventional microfabrication. These deposition processes define film thickness, morphology, and interfacial quality, directly affecting ion transport, transconductance, and switching behavior in OECTs, which are critical for large‐scale neuromorphic arrays and integrated bioelectronic systems.

### 3.2 Additive and Emerging Manufacturing Technologies

The limitations of conventional lithography‐based fabrication processes have highlighted the need for alternative manufacturing strategies for OECTs. In particular, polymer‐photoresist incompatibility and multistep processing increase cost, process complexity, and material waste, thereby limiting scalable fabrication and integration across diverse substrates. In contrast, AM provides a fundamentally different fabrication paradigm based on direct material deposition [19, 23]. Importantly, AM processing does not require extensive processing steps, reducing material consumption and enabling low‐cost large‐area fabrication [19, 21]. Furthermore, direct fabrication on non‐planar, soft, and flexible substrates significantly broadens applications toward wearable and biointegrated systems [23].

Currently, various AM techniques, including inkjet printing, aerosol‐jet printing, and transfer printing, have attracted considerable attention for OECT fabrication [124]. These approaches allow direct patterning of functional materials, resulting in on‐demand device fabrication by precisely controlling the channel geometry, volume, and electrolyte configuration. As a result, AM‐based OECTs have demonstrated excellent versatility across various bioelectronic applications, such as sensors, logic circuits, active matrices, and healthcare monitoring. Notably, conventional neuromorphic hardware relies on photolithography and thin‐film deposition, which limits structural flexibility and makes it difficult to mimic complex three‐dimensional neural networks. AM facilitates the fabrication of multi‐material three‐dimensional architectures, creating new opportunities for artificial synapses and neuromorphic circuits [18]. From this standpoint, it accelerates the development of OECT‐based systems tailored for soft and biointegrated platforms. In this section, we introduce representative AM strategies and confirm their fabrication principles and applications.

#### 3.2.1 Inkjet Printing

**FIGURE 15:**
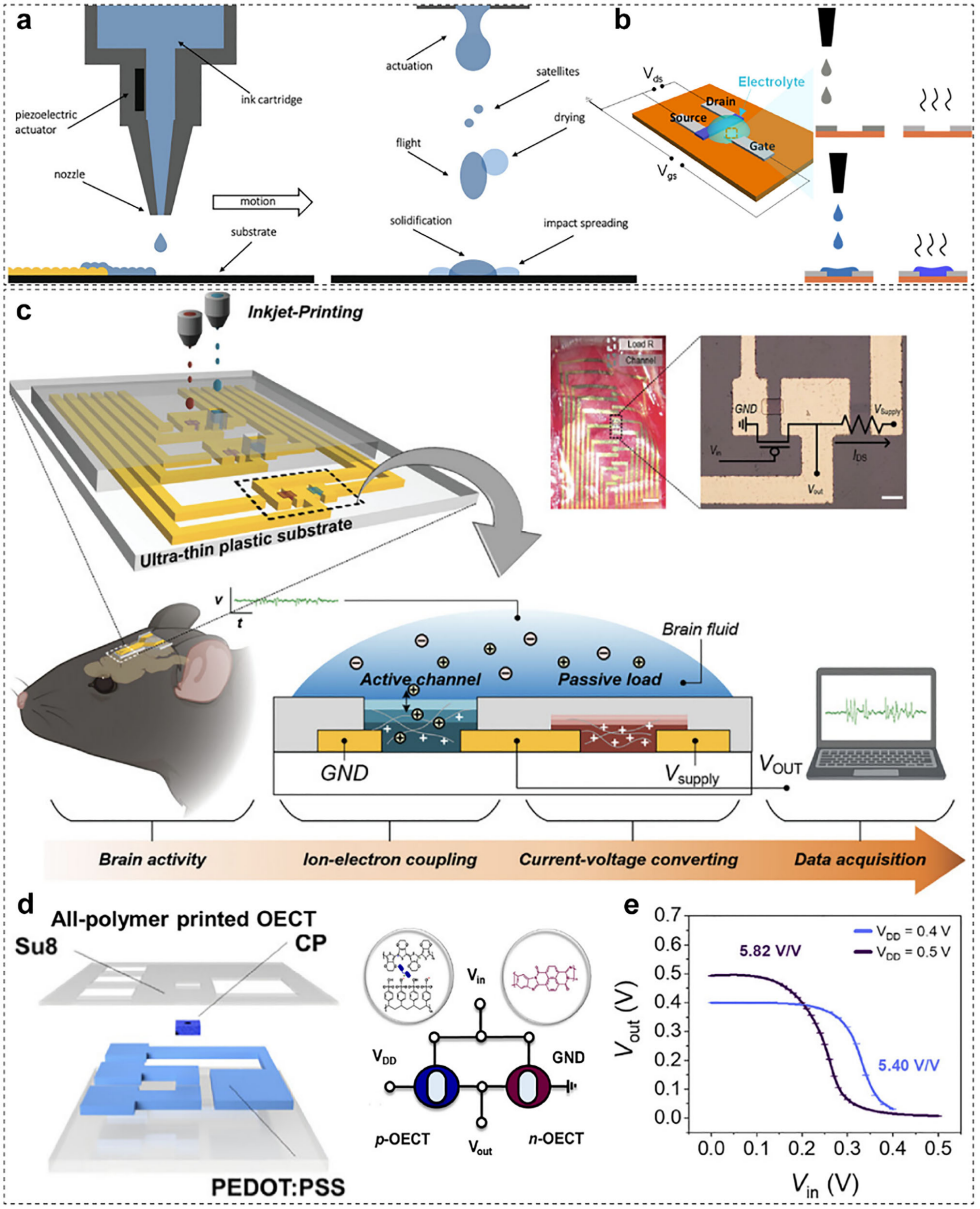
Inkjet printing‐based AM strategies. (a) Schematic illustration of the inkjet printing technique, where ink droplets are ejected from a nozzle for patterned material deposition. Evolution of ink droplets during the printing process. Reproduced under the terms of the CC‐BY license [19]. Copyright 2025, The Authors, published by Wiley‐VCH. (b) Schematic illustration of OECT device structure and biasing configuration during operation, alongside cross‐sectional fabrication steps highlighting seamless multimaterial integration enabled by inkjet printing. Reproduced with permission [129]. Copyright 2022, ACS. (c) Layout and optical micrographs of inkjet‐printed Electrocorticography probes integrating an active OECT and a passive resistor for neural signal acquisition. Reproduced with permission [130]. Copyright 2023, Wiley‐VCH. (d) Schematic and photograph of an all‐printed OECT fabricated by inkjet printing. Complementary device configuration composed of p‐type and n‐type channels printed on the same substrate. (e) Inverter characteristics of an inkjet‐printed OECT. Reproduced under terms of the CC‐BY license [131]. Copyright 2025, The Authors, published by Wiley‐VCH.

Among various emerging additive manufacturing technologies, inkjet printing has garnered considerable attention as a high‐precision patterning strategy for soft and wearable OECTs [125, 126]. Inkjet printing is a non‐contact, digitally controlled direct‐write additive technique in which low‐viscosity inks containing active materials are expelled from nozzles and propelled toward the substrate surface, as schematically depicted in Figure 15a. As inkjet printing is a programmable process that selectively deposits material at predefined coordinates, it minimizes material waste. Moreover, the technique eliminates the requirement for masks, enabling accurate patterning without complex photolithographic steps. Consequently, these advantages establish inkjet printing as a compelling AM platform for the fabrication of flexible electronics and wearable bioelectronic devices [18, 19, 21, 22, 127, 128].

Notably, inkjet printing is particularly well‐suited for OECT fabrication due to its capability to precisely pattern conjugated polymers and other organic materials [19]. Specifically, inkjet printing supports the deposition of not only the semiconducting channel but also electrodes and solid‐state electrolytes within the same process framework (Figure 15b) [129]. From a processing standpoint, the technique can be implemented under low‐temperature conditions, ensuring compatibility with flexible and biodegradable substrates. Recent studies have demonstrated that inkjet printing serves as an effective fabrication platform. For example, the monolithic integration of OECTs and polymer resistors on a single flexible substrate via inkjet printing has enabled near‐sensor processing architectures (Figure 15c) [130]. This approach establishes the feasibility of integrating active amplification circuits within conformal neural interfaces. Additionally, inkjet printing has demonstrated the capability to extend beyond individual device toward logic‐level integration (Figure 15d,e) [131]. As a result, this technique has evolved beyond a simple patterning tool into a versatile, material‐efficient, and integration‐friendly manufacturing paradigm for next‐generation OECT‐based neuromorphic and biointerface systems.

#### 3.2.2 Direct Ink Writing: Extrusion‐Based Printing

**FIGURE 16:**
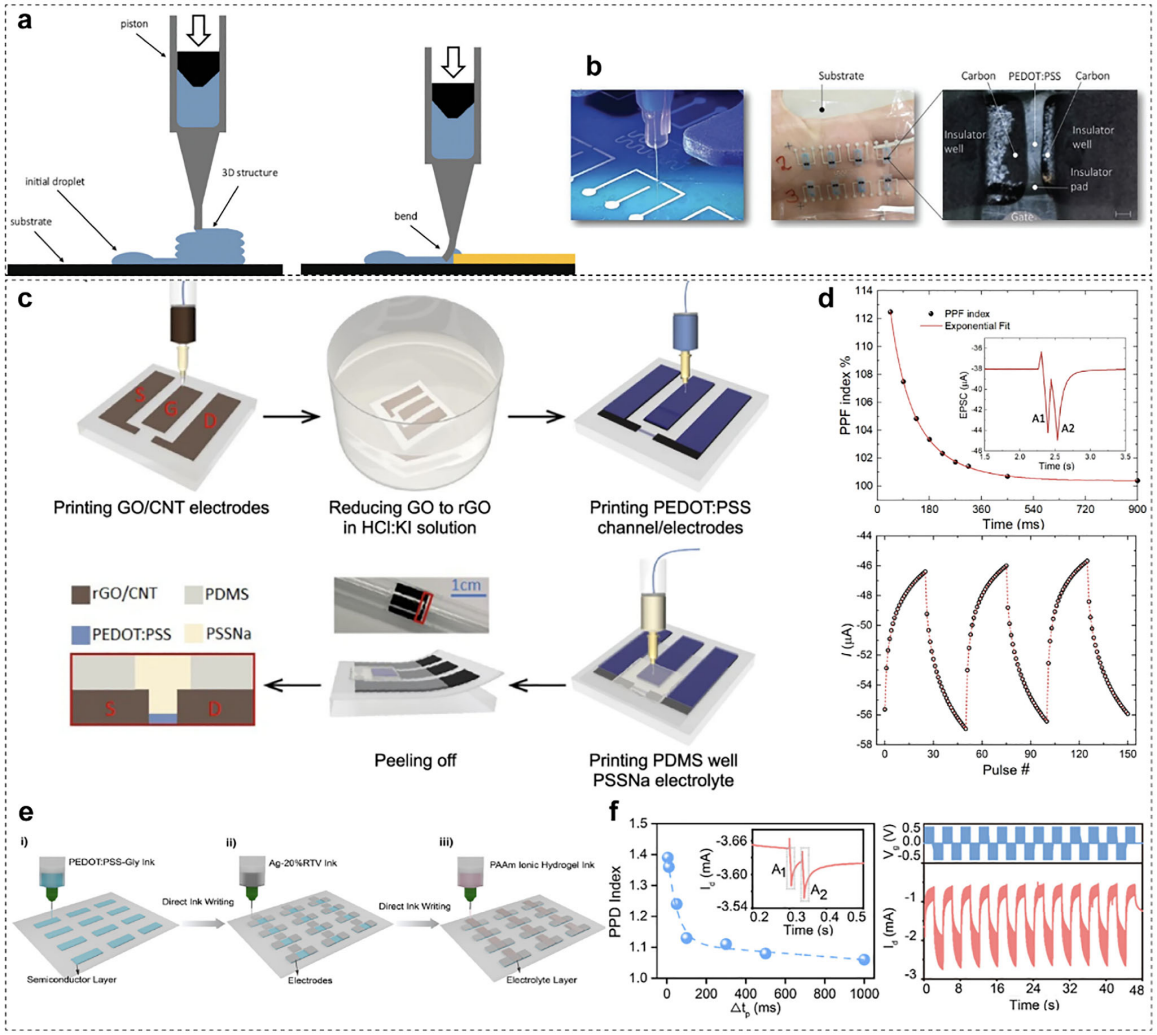
Extrusion printing‐based AM strategies. (a) Schematic illustration of extrusion‐based DIW printing. Functional inks are continuously extruded through a nozzle and deposited layer‐by‐layer. Reproduced under the terms of the CC‐BY license [19]. Copyright 2025, The Authors, published by Wiley‐VCH. (b) Photographs of the DIW printing system and printed OECT devices. Reproduced under the terms of the CC‐BY‐NC license [135] Copyright 2022, The Authors, published by Wiley‐VCH. (c) Schematic illustration of the fabrication process for fully printed OECTs using extrusion‐based 3D printing. (d) PPF characteristics of the printed OECT synapse operating in the short‐term doping/dedoping regime. Long‐term depression (LTD) and potentiation characteristics of the printed OECT synapse. Reproduced under the terms of the CC‐BY license [136] Copyright 2023, The Authors, published by Springer Nature. (e) Flowchart of the fabrication process for a fully printed stretchable ionic synaptic OECT (is‐OECT) array using a custom‐built multimaterial extrusion printer equipped with multiple dispensing heads. (f) PPD characteristics of the is‐OECT synapse. Potentiation and depression behavior of the printed is‐OECT synapse, demonstrating stable long‐term synaptic plasticity. Reproduced with permission [137]. Copyright 2023, ACS.

Another emerging AM technique, extrusion printing, has also gained attention as an alternative fabrication approach for constructing multidimensional and highly functional structures. As schematically illustrated in Figure 16a, extrusion printing is an AM process capable of fabricating two‐ and three‐dimensional architectures through layer‐by‐layer deposition [18, 19, 132, 133, 134]. Extrusion printing is particularly suitable for the fabrication of OECTs because it enables stable processing of high‐viscosity functional inks and sequential deposition of channel, electrode, and electrolyte layers. Furthermore, the capability to directly print on unconventional substrates provides significant process versatility for bioelectronic and neuromorphic applications. As illustrated in Figure 16b, these advantages, including broad material compatibility, structural design freedom, and simplified fabrication workflows, make extrusion printing a promising manufacturing strategy for fully printed OECTs [19, 135]. For example, recent studies have demonstrated fully printed OECTs in which all components are directly deposited via wet extrusion‐based 3D printing (Figure 16c) [136]. The devices exploit the volumetric ion–electron coupling characteristic of OECT operation to emulate synaptic plasticity and exhibit neuromorphic functionalities (Figure 16d). In addition, stretchable OECT arrays have been realized by sequentially depositing functional inks with different viscosities through multi‐material extrusion printing (Figure 16e) [137]. These devices reproduce key synaptic behaviors, including STP and LTP (Figure 16f), while operating stably under mechanical deformation. Therefore, these results highlight extrusion printing as an effective manufacturing platform for neuromorphic bioelectronic systems capable of constructing complex multilayer architectures and soft biointerfaces.

#### 3.2.3 Aerosol Jet Printing and Hybrid Processes

While inkjet printing and extrusion‐based printing, introduced in the previous sections, offer distinct advantages for printed OECT fabrication, both technologies also present intrinsic process limitations. Inkjet printing provides high resolution and precise patterning but is restricted to low‐viscosity inks and is less suitable for fabricating thick structures. In contrast, extrusion‐based printing is well suited for high‐viscosity inks and the fabrication of three‐dimensional structures but suffers from comparatively lower resolution and limited fine patterning capability. From this perspective, aerosol jet printing (AJP) may become a promising alternative [18].

**FIGURE 17:**
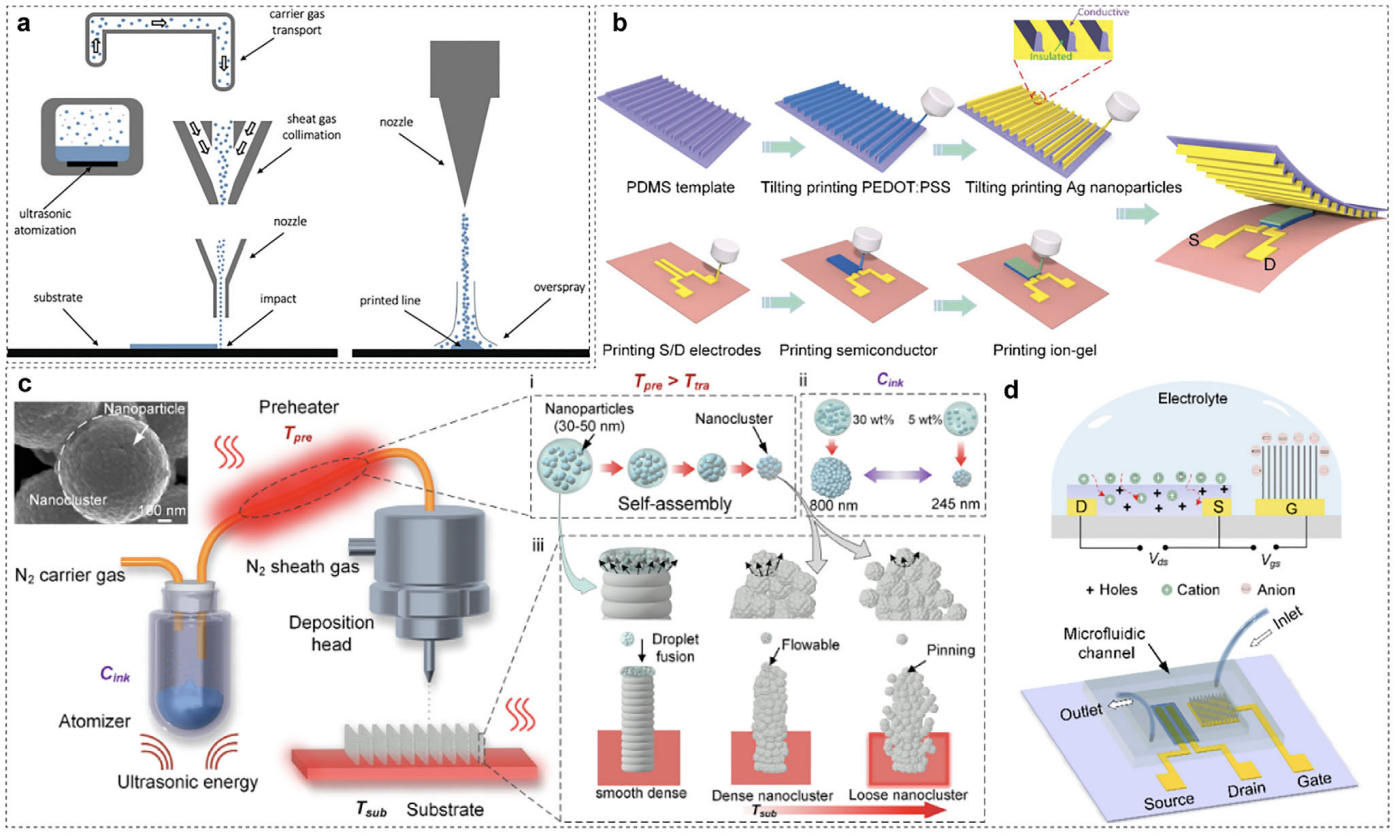
Aerosol jet printing‐based AM strategies. (a) Schematic illustration of the key processes in AJP. Functional inks are atomized into microscale droplets to form an aerosol mist, which is transported by a carrier gas and collimated by a sheath gas into a focused jet for deposition through a nozzle onto the substrate. Reproduced under the terms of the CC‐BY license [19]. Copyright 2025, The Authors, published by Wiley‐VCH. (b) Additive fabrication process of an aerosol jet printed OECT force sensor. Sequential AJP deposition enables the formation of microstructure electrodes. Reproduced with permission [139]. Copyright 2023, Wiley‐VCH. (c) Schematic illustration of the aerosol jet printing system. The resulting micro–nano hierarchical structures can be tuned by controlling the preheating temperature, Ag nanoparticle ink concentration, and substrate temperature. (d) Schematic diagram of OECT operation. Illustration of an AJP‐fabricated OECT platform. Reproduced under the terms of the CC‐BY license [140]. Copyright 2023, The Authors, published by ACS.

AJP is a noncontact, direct‐write AM technique that deposits functional materials by transforming inks into a fine aerosol (Figure 17a). AJP can process inks across a wide viscosity range and achieve high‐precision patterning with feature sizes on the order of tens of micrometers [19, 21, 22, 127, 128, 138]. The high‐resolution, maskless, and non‐contact nature of AJP facilitates precise patterning of OECT components and supports multimaterial, multilayer architectures compatible with neuromorphic device fabrication. These capabilities are especially advantageous for OECT‐based neuromorphic systems that require precise device arrays and multimaterial integration, expanding opportunities for complementary OECT circuits and densely integrated neural‐mimetic devices [19, 21]. Consequently, AJP is increasingly regarded as a promising manufacturing approach for next‐generation printed OECTs and neuromorphic bioelectronic platforms. Recent studies have demonstrated that AJP supports the fabrication of complex OECT‐based sensory and biointerfacing devices (Figure 17b) [139]. Microstructured gate electrodes fabricated via AJP effectively amplify interfacial responses induced by mechanical stimuli, leading to active multidimensional tactile sensing functionalities. In addition, three‐dimensional microarchitectures produced through AJP enhance ion transport and electrochemical reactions, resulting in highly sensitive detection of biochemical signals such as neurotransmitters (Figure 17c,d) [140]. These findings highlight the potential of AJP as an effective fabrication strategy for neuromorphic OECT systems.

**FIGURE 18:**
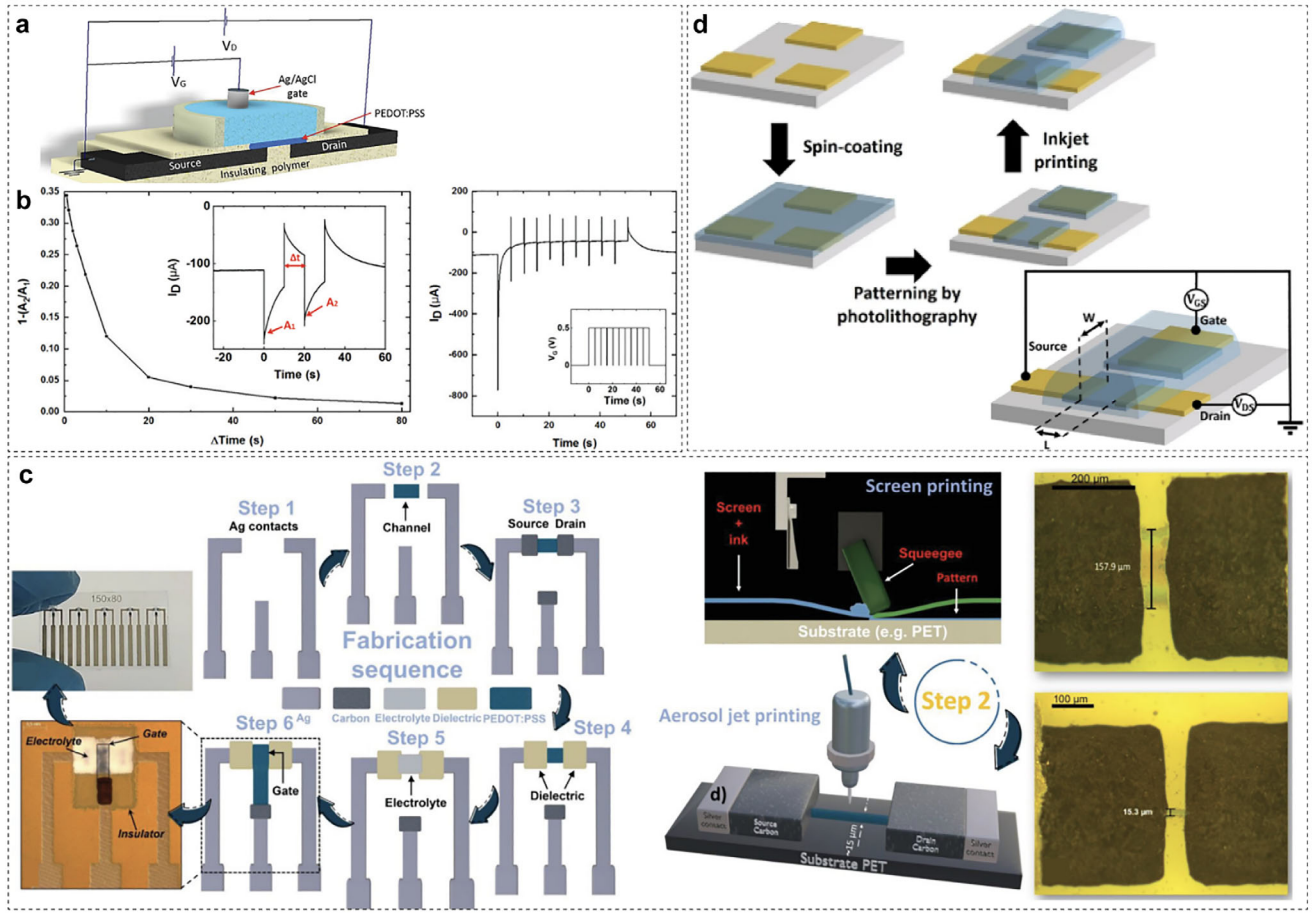
Hybrid manufacturing strategies. (a) Schematic illustration of an additively manufactured OECT using inkjet printing and 3D printing. (b) Paired‐pulse depression characteristics of the OECT device. Adaptive response of the OECT under a train of gate voltage pulses. Reproduced under the terms of the CC‐BY license [141]. Copyright 2022, The Authors, published by Wiley‐VCH. (c) Hybrid fabrication sequence combining multiple printing techniques for OECT manufacturing. The process integrates screen printing and aerosol jet printing to deposit different device layers. Optical microscope images compare screen‐printed and aerosol jet printed OECT channels. Reproduced under the terms of the CC‐BY‐NC license [142]. Copyright 2022, The Authors, published by Wiley‐VCH. (d) Schematic of a hybrid OECT fabrication strategy integrating photolithography and inkjet printing. Reproduced under the terms of the CC‐BY license [143]. Copyright 2023, The Authors, published by Springer Nature.

More recently, hybrid fabrication strategies that combine multiple printing techniques have been explored as viable routes toward integrated bioelectronic systems. Hybrid approaches aim to exploit the complementary strengths of different fabrication methods. For instance, OECTs fabricated via hybrid direct‐write processes integrating 3D printing and inkjet printing have demonstrated neuromorphic processing behaviors (Figure 18a), including synaptic plasticity and adaptive responses, in which memory and computation coexist within a single device (Figure 18b) [141]. Similarly, the combination of screen printing and aerosol jet printing achieves both large‐area manufacturability and high‐resolution patterning (Figure 18c), allowing the fabrication of high‐speed OECT logic circuits and integrated inverters [142].

However, despite these efforts, achieving the resolution and integration density required for highly integrated electronic systems remains a significant challenge. For this reason, hybrid manufacturing strategies that combine AM with top–down microfabrication approaches have attracted increasing attention. In particular, lithography‐based processes offer high resolution, excellent pattern fidelity, and dense device integration, whereas AM enables direct patterning of functional materials, fabrication of three‐dimensional architectures, and compatibility with flexible and biocompatible substrates. Therefore, combining the strengths of these two approaches may provide a practical route to overcome resolution limitations of printed bioelectronics while achieving higher levels of device integration and system‐level functionality. For example, hybrid processes integrating orthogonal photolithography with inkjet printing provide micrometer‐scale control over transistor geometries, significantly improving device uniformity (Figure 18d) [143]. Therefore, the development of additive and hybrid manufacturing technologies serves as a key driving force for advancing OECTs beyond individual devices toward integrated neuromorphic systems.

### 3.3 Device Architectures Enabled by Manufacturing Strategies

Recent advances in AM have significantly expanded the design freedom of OECTs. These manufacturing technologies are no longer limited to process steps for device formation but instead serve as central design parameters that determine device geometry, material distribution, and functional integration. A typical OECT is a three‐terminal active device consisting of gate, source, and drain electrodes, an OMIEC channel positioned between the source and drain, and an electrolyte that ionically connects the channel and the gate. Depending on the relative position of the gate electrode, the electrode arrangement, and the overall device configuration, OECTs can be designed in a variety of architectures. This structural flexibility is closely associated with fabrication strategies, and recent advances in AM provide greater freedom in realizing diverse device geometries and integrated structures. Consequently, the evolution of OECT systems is shifting toward a design paradigm that integrates manufacturing strategies and device architectures. In this section, we focus on OECT structures arising from advanced manufacturing approaches and discuss how structural design contributes to neuromorphic information processing and next‐generation bioinspired electronic systems [19, 21, 57].

#### 3.3.1 Planar and Vertical Device Architectures

**FIGURE 19:**
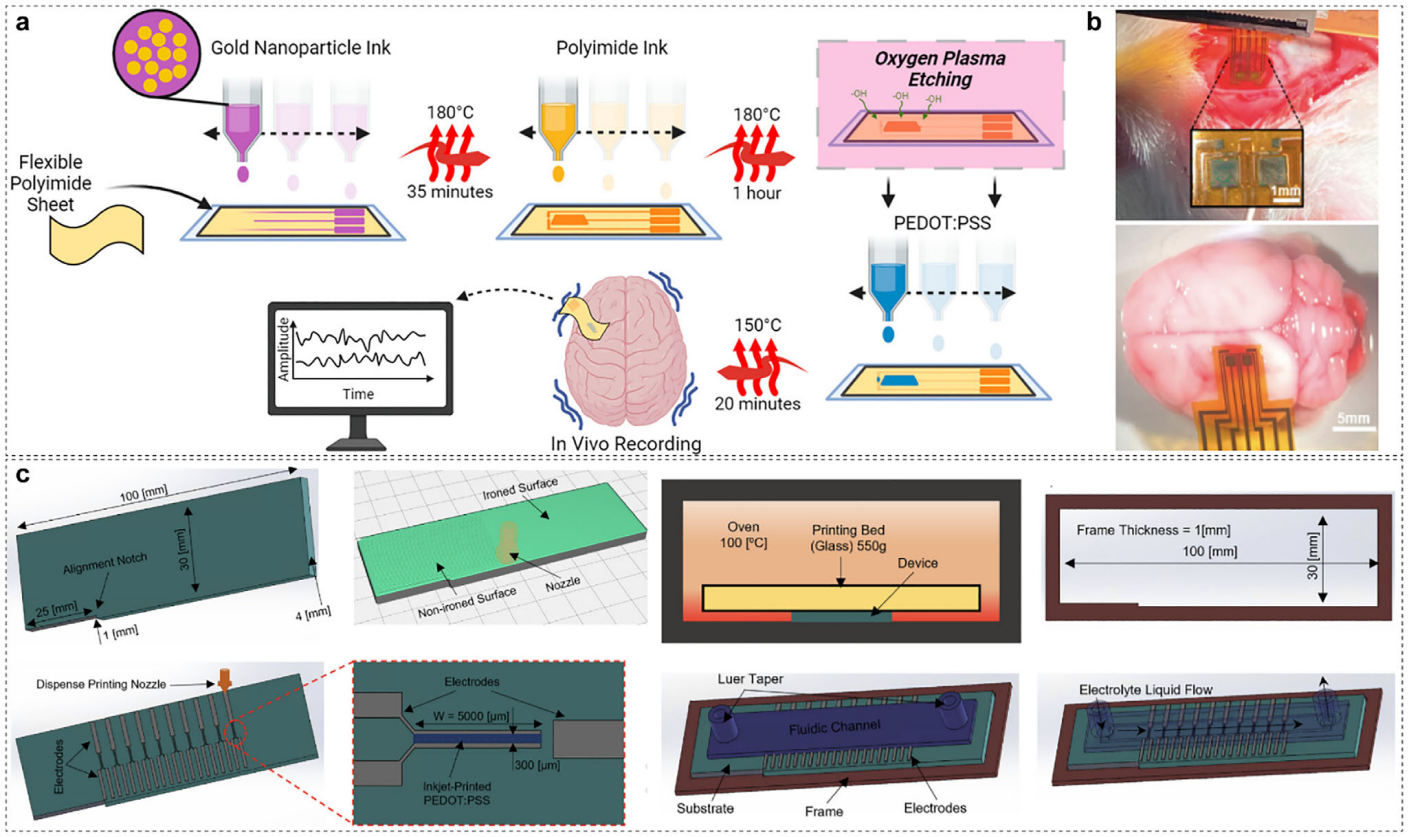
Planar OECT device architectures. (a) Fabrication process of inkjet‐printed planar OECTs. (b) Photographic images of a planar OECT device used for in vivo neural signal recording. Reproduced under the terms of the CC‐BY license [144]. Copyright 2024, The Authors, published by ACS. (c) Additive fabrication process of a planar OECT integrated with a 3D‐printed substrate. The substrate is first fabricated via FDM printing with surface ironing to reduce roughness. Silver electrodes are then deposited by dispensing printing, followed by inkjet printing of the PEDOT:PSS channel. Reproduced under the terms of the CC‐BY license [145]. Copyright 2025, The Authors, published by Wiley‐VCH.

The operating properties of OECTs depend strongly on ion transport pathways and channel geometry. Therefore, device architecture functions as a critical factor governing information processing behavior and operational modes. Recent advances in manufacturing technologies have led to the redesign of conventional planar configurations, providing expanded design flexibility for neuromorphic functionalities. For example, OECTs fabricated via inkjet printing have realized highly reproducible fully planar devices without relying on complex cleanroom microfabrication processes (Figure 19a) [144]. In particular, compact planar channel geometries are advantageous for neural signal recording requiring high spatial resolution, and successful detection of distinct seizure phases with high signal‐to‐noise ratios has been demonstrated in vivo (Figure 19b). This research suggests that printing‐based fabrication approaches extend beyond simplifying device production by enabling the optimization of ion–electron interactions and signal amplification characteristics within planar architectures, thereby supporting OECT designs for neural interfacing. Moreover, the direct realization of OECTs on planarized 3D‐printed substrates through the integration of various printing processes has highlighted that fabrication strategies themselves significantly influence device architecture and performance [145]. Figure 19c illustrates a hybrid AM process is employed, in which surface ironing during 3D printing reduces substrate roughness, followed by dispense printing of low‐resistance silver electrodes and inkjet deposition of PEDOT:PSS channels. This process of integration results in fully additive planar OECTs achieving high transconductance and low contact resistance. This co‐design of fabrication processes and device architecture promotes the development of customized bioelectronic and neuromorphic systems.

**FIGURE 20:**
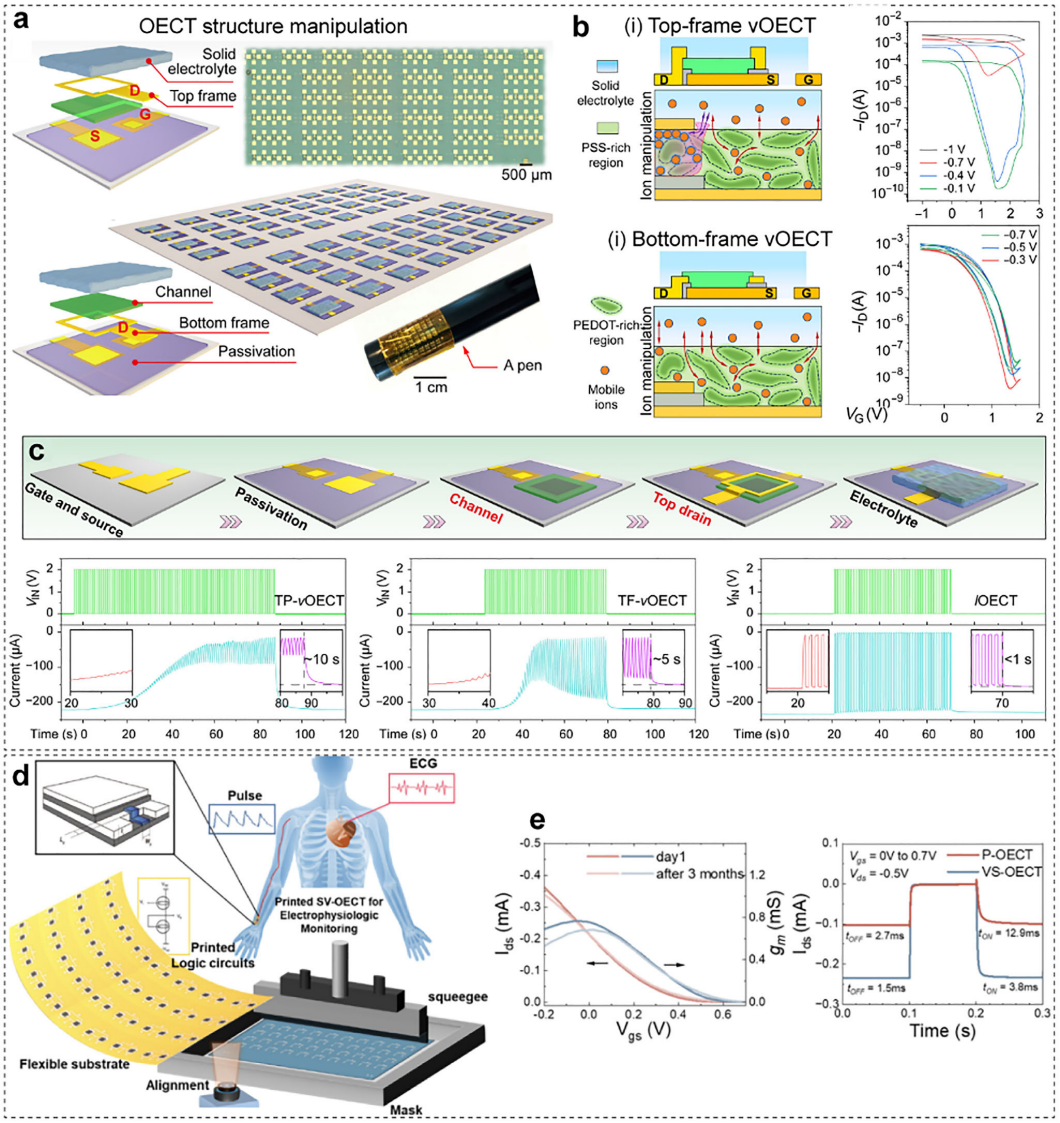
Vertical OECT device architectures. (a) Schematic illustration of structure manipulation in solid‐state vertical OECTs, comparing top‐frame and bottom‐frame drain configurations. Monolithic fabrication of vertical OECT arrays enables integration of densely patterned devices on both rigid and flexible substrates. (b) Device structures and electrical characteristics of vertical OECTs with (i) top‐frame drain and (ii) bottom‐frame drain configurations. The relative position of the drain electrode with respect to the PEDOT:PSS channel modulates ion transport pathways and device switching behavior. (c) Monolithic integration process of solid‐state vertical OECTs. Synaptic plasticity characteristics of TP‐vOECTs, TF‐vOECTs, and conventional IOECTs are compared. Reproduced under the terms of the CC‐BY license [59]. Copyright 2025, The Authors, published by AAAS. (d) Schematic illustration of the screen‐printing fabrication process for vertical step OECTs (VS‐OECTs). (e) Transfer characteristics and transconductance of the VS‐OECT measured on the first day and after three months, demonstrating long‐term device stability. Transient response of VS‐OECTs with different structural configurations, highlighting the rapid response enabled by the shortened ion transport pathway in the vertical step architecture. Reproduced with permission [58]. Copyright 2026, ACS.

However, while planar OECT architecture offers advantages in spatial resolution and fabrication simplicity, it faces limitations in simultaneously optimizing ionic accessibility, switching speed, and integration density. Although increasing the effective ion‐accessible volume and surface area can enhance ion–electron interactions, excessive channel dimensions may lead to increased parasitic capacitance and longer ion transport distances, ultimately reducing switching speed. Consequently, various three‐dimensional device architectures have attracted increasing attention as strategies to improve ionic accessibility while minimizing parasitic effects. Among them, vertically configured OECT architectures have emerged as a promising approach owing to their compact footprint and tunable ion transport pathways [119, 146]. In particular, vertical solid‐state OECTs based on monolithic integration processes have been established by stacking channel materials between source and drain electrodes [59]. In these devices, microscale patterning of polyelectrolyte (pDADMAC) and PEDOT:PSS channels enables precise regulation of large polycation transport pathways (Figure 20a). As a result, when the drain electrode is positioned above the channel, restricted ion movement produces synaptic plasticity characteristic of neuromorphic operation, whereas placement beneath the channel facilitates faster ionic responses, leading to logic‐type switching behavior (Figure 20b,c). These findings emphasize that vertical OECT architectures can serve as functional design parameters for next‐generation neuromorphic bioelectronic systems. More recently, all‐printed vertical step OECTs (VS‐OECTs) have been realized using screen‐printing processes (Figure 20d), in which the source and drain electrodes are vertically stacked with a thin channel layer formed between them [58]. This structural configuration helps overcome the resolution and speed limitations observed in conventional planar OECTs. In the vertical step structure, ion injection pathways are shortened through direct contact between the channel and the electrolyte, resulting in both high transconductance and rapid transient responses (Figure 20e). This study further demonstrates that fabrication strategies play a decisive role in defining vertical architectures and improving signal amplification performance, supporting the development of printed neuromorphic platforms and flexible bioelectronic systems. These results suggest that vertical OECT architecture represents one of the most practical design strategies for balancing ionic accessibility, integration density, and switching speed. In addition, fin‐like geometries and other high‐surface‐area architectures may provide further opportunities to enhance ion–electron interactions. Such architectures are considered promising design directions for future high‐performance OECT‐based neuromorphic bioelectronic systems.

#### 3.3.2 Flexible and Wearable Devices

Because the primary applications of OECTs involve direct interaction with biological tissues, mechanical compatibility with soft and dynamic biological environments has become an essential design consideration. In particular, devices should be flexible, stretchable, and conformal to biological tissues to ensure stable interfacial contact and reliable signal acquisition, even under continuous motion and deformation. The realization of such mechanically adaptive devices has been closely associated with advances in manufacturing technologies. As a result, OECTs have evolved from rigid laboratory‐scale platforms into flexible and wearable systems, leading to diverse device architectures, including skin‐conformal sensors, wearable physiological monitoring systems, and neuromorphic bioelectronic platforms [24, 57, 147, 148, 149, 150, 151].

**FIGURE 21:**
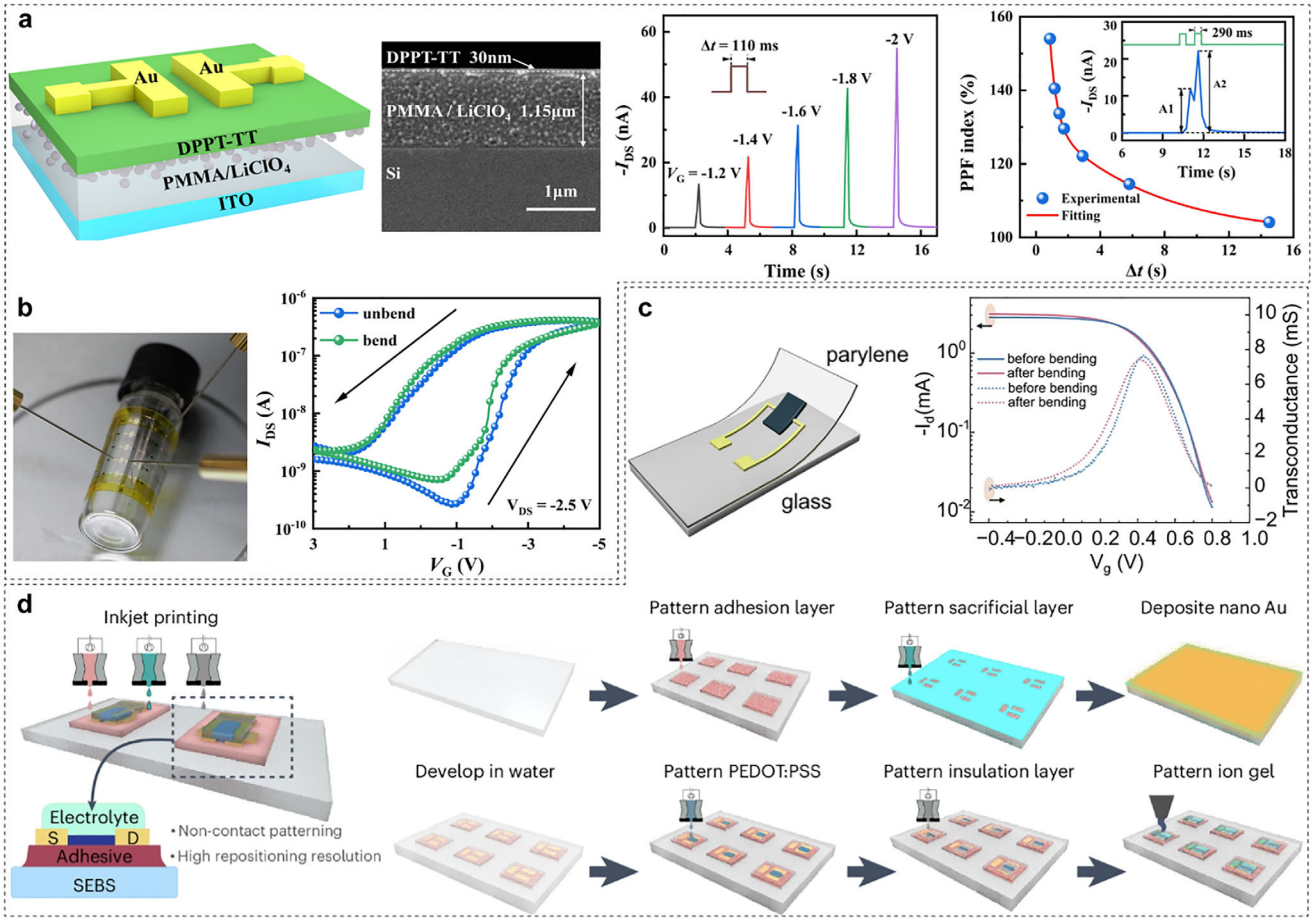
Flexible and wearable OECT architectures. (a) Schematic device structure and cross‐sectional SEM image of a flexible OECT device based on a DPPT‐TT organic semiconductor and a solid‐state electrolyte. EPSC responses under single‐pulse stimulation and the PPF index demonstrate synaptic plasticity characteristics. (b) Optical image of the flexible OECT device under mechanical bending. Transfer characteristics of the device demonstrate stable electrical performance under mechanical deformation. Reproduced under the terms of the CC‐BY license [152]. Copyright 2024, The Authors, published by Multidisciplinary Digital Publishing Institute (MDPI). (c) Schematic illustration of a flexible OECT. Transfer curves measured before bending and after mechanical recovery confirm the mechanical robustness of the device. Reproduced under the terms of the CC‐BY‐NC‐ND license [153]. Copyright 2025, The Authors, published by Springer Nature. (d) Fabrication and architecture of stretchable OECT arrays produced via multichannel inkjet printing. Reproduced with permission [154]. Copyright 2024, Springer Nature.

For instance, recent studies have demonstrated ultralow‐power neuromorphic devices based on OECTs fabricated on flexible substrates, achieving both mechanical compliance and synaptic computing functionality (Figure 21a,b) [152]. Flexible OECTs employing a DPPT‐TT organic semiconductor and a solid‐state electrolyte operate at low voltages and reproduce synaptic plasticity behaviors through electric double‐layer effects while maintaining extremely low energy consumption. These findings indicate that flexible OECTs are promising platforms for energy‐efficient neuromorphic computing and wearable artificial intelligence systems. In addition to printing‐based AM approaches, complementary fabrication strategies such as electropolymerization have been explored to enhance the mechanical robustness of flexible OECTs (Figure 21c) [153]. Furthermore, OECTs have progressed toward wearable in‐sensor computing systems that integrate sensing and computation [154]. Stretchable OECT arrays fabricated via inkjet printing, as shown in Figure 21d, perform biosignal acquisition and processing directly at the device level while maintaining high fabrication yield and stretchability exceeding 50%. Collectively, these studies demonstrate that the co‐design of fabrication processes and device architectures plays a central role in the development of wearable neuromorphic bioelectronic systems.

### 3.4 Electrolyte Configurations Enabled by Manufacturing

In OECTs, the electrolyte is a critical component that governs device operation. It ionically couples the gate electrode and the channel and induces electrochemical doping and dedoping through ion penetration into the active material. The most conventional electrolyte configuration is an aqueous liquid electrolyte. Electrolyte solutions such as physiological saline and phosphate‐buffered saline (PBS) provide high ionic conductivity and rapid ion transport, resulting in fast switching and high transconductance. Moreover, they are suitable for wearable and implantable biosignal sensors due to their superior biocompatibility. Despite these advantages, liquid electrolytes present inherent limitations in terms of structural and operational stability. Their fluidic nature can result in leakage and evaporation, leading to degradation of interfacial stability under prolonged operation [20, 49, 73, 155, 156]. These issues pose challenges for device miniaturization, system integration, and long‐term biointerfaced applications. Accordingly, extensive efforts have been directed toward replacing liquid electrolytes, including the incorporation of gel electrolytes.

#### 3.4.1 Gel and Hydrogel Electrolytes

Conventional liquid electrolyte‐based OECTs suffer from several intrinsic limitations, including leakage, evaporation, difficulties in patterning, and limited compatibility with large‐area integration. These limitations impose fundamental constraints on long‐term operational stability and scalable manufacturing processes. To overcome these issues, gel or hydrogel electrolytes have been proposed as alternative materials. Gel electrolytes consist of polymer networks that immobilize water or ionic liquids within a crosslinked matrix. Such networks maintain high ionic conductivity while simultaneously providing structural stability and mechanical flexibility. In contrast to liquid electrolytes, gel‐based systems allow precise control over electrolyte thickness, lateral dimensions, geometry, and spatial positioning through various fabrication strategies, including printing, photopatterning, and layer‐by‐layer deposition processes. As a result, manufacturing‐defined electrolyte configurations can directly modulate key OECT parameters such as response speed, power consumption, and operational stability. In neuromorphic applications, ionic transport dynamics are closely correlated with synaptic weight modulation and plasticity behaviors. Therefore, gel and hydrogel electrolytes serve as key platforms that integrate material design, device architecture, and neuromorphic functionality through ion transport pathways defined by the manufacturing process [20, 157, 158, 159].

**FIGURE 22:**
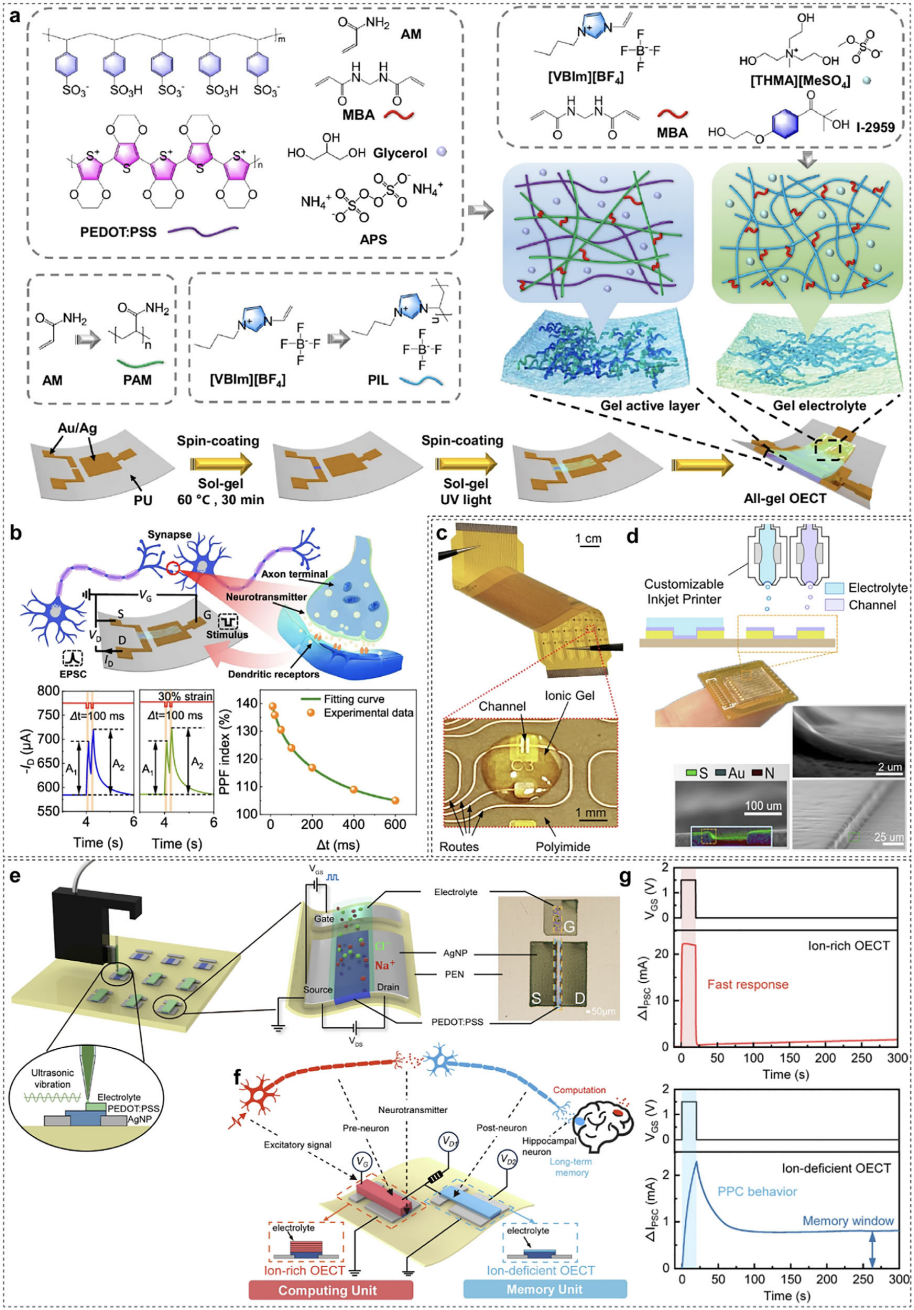
OECT systems with gel‐ and hydrogel‐based electrolyte. (a) Fabrication strategy of all‐gel OECTs. The three‐dimensional gel network promotes efficient ion penetration and transport, enabling fully gel‐integrated transistor architectures. (b) Operating mechanism and synaptic behavior of an all‐gel OECT. EPSC responses triggered by two consecutive pulses are compared before and after 30% mechanical strain. The PPF index demonstrates synaptic plasticity. Reproduced under the terms of the CC‐BY‐NC‐ND license [111]. Copyright 2025, The Authors, published by Springer Nature. (c) Optical images of electrodes fabricated using a flexible printed circuit board (fPCB) process. (d) Hybrid manufacturing strategy combining fPCB processing with inkjet printing. A customized inkjet printer is used to pattern the PEDOT:PSS channel and gel electrolyte. Reproduced under the terms of the CC‐BY‐NC‐ND license [160]. Copyright 2025, The Authors, published by Springer Nature. (e) Direct printing approach for fully printed OECTs using an ultrasonic vibration‐assisted dispenser. The schematic and optical micrographs illustrate the architecture of an all‐printed OECT integrating electrolyte, semiconductor channel, and electrodes. (f) Conceptual illustration of neuromorphic computing systems based on OECT architectures that emulate biological neural networks, where distinct devices function as computing and memory units. (g) Pulse responses of ion‐rich and ion‐deficient OECTs controlled through electrolyte layer configuration. The ion‐rich OECT (multilayer electrolyte) exhibits rapid transient responses, whereas the ion‐deficient OECT (single‐layer electrolyte) shows persistent responses suitable for memory functions. Reproduced under the terms of the CC‐BY license [161]. Copyright 2025, The Authors, published by Springer Nature.

Recent studies have actively explored gel‐based electrolyte systems to simultaneously improve the mechanical robustness and neuromorphic functionality of OECTs [111]. In particular, all‐gel OECTs have combined a poly(ionic liquid) (PIL)‐based ionogel electrolyte with a PEDOT:PSS/PAM double‐network semiconducting gel channel, in which the three‐dimensional gel network efficiently promotes ion penetration and transport (Figure 22a). As a result, the device exhibited a high transconductance (86.4 mS) and a large on/off ratio while maintaining 50% stretchability and stable operation under repeated mechanical deformation. Moreover, Figure 22b demonstrates synaptic behaviors, including EPSC, PPF, and learning–forgetting–relearning processes, suggesting the potential of gel‐integrated OECT architectures as stretchable artificial synaptic platforms.

Meanwhile, the advantages of gel electrolytes extend beyond the material level to manufacturing and system‐integration strategies. For example, a hybrid fabrication approach combining commercial flexible printed circuit board (fPCB) processing with inkjet printing has been reported. In this strategy, electrodes, interconnects, and insulating layers are first defined using established fPCB processes, followed by patterned deposition of the PEDOT:PSS channel and gel‐based electrolyte via inkjet printing (Figure 22c,d) [160]. This process flow effectively restructures conventional fabrication routes for flexible OECTs. Unlike liquid electrolytes, gel electrolytes can be deposited as printable solid layers at predefined locations. This characteristic allows the electrolyte to be naturally incorporated into fabrication steps, thereby contributing to the utilization of OECT nonlinear ionic dynamics in neuromorphic in‐sensor computing and rapid prototyping of integrated systems. Furthermore, the implementation of neuromorphic systems requires the coexistence of volatile elements for rapid signal preprocessing and non‐volatile elements for memory retention within the same platform, analogous to biological neural networks [161]. Conventionally, transitions between volatile and non‐volatile behavior have been achieved by modifying material composition or bias conditions. However, such strategies often increase process incompatibility and circuit complexity. To address these limitations, recent studies have shifted the focus toward electrolyte structural design (Figure 22e,f). In other words, precise control over the thickness and layer configuration of the electrolyte allows the areal ionic concentration within the channel to be defined at the manufacturing stage (Figure 22g). Consequently, the electrolyte evolves from a static material component into a three‐dimensionally engineered ionic architecture.

#### 3.4.2 Other Solid Electrolytes

**FIGURE 23:**
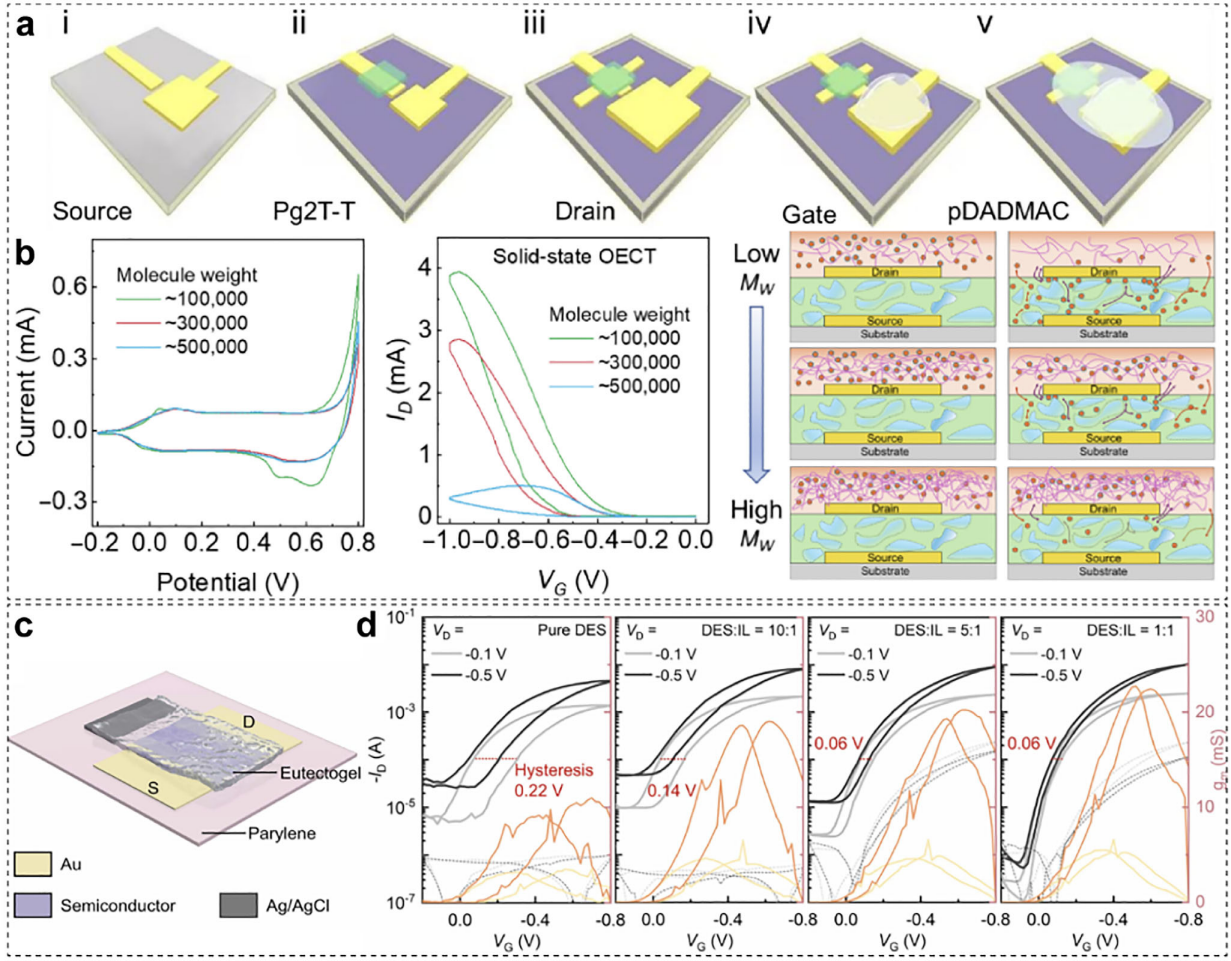
Polymer and eutectogel electrolytes for OECT devices. (a) Stepwise fabrication process of a solid‐state OECT using a polyelectrolyte electrolyte. (b) Electrochemical and transistor characteristics of OECT gated by pDADMAC electrolytes with different molecular weights. The schematic illustrates how electrolyte molecular weight regulates ion transport and electrochemical doping processes. Reproduced under the terms of the CC‐BY license [163]. Copyright 2025, The Authors, published by MDPI. (c) Schematic illustration of OECT architecture incorporating an eutectogel electrolyte layer. (d) Transfer characteristics and corresponding transconductance of OECTs gated by eutectogel electrolytes with different DES: IL mass ratios. Reproduced with permission [164]. Copyright 2026, Elsevier Inc.

Polymer electrolytes, where ionic transport occurs through a polymer matrix, have also been explored as solid‐state electrolyte materials for OECT devices, complementing the use of gel electrolytes. Polyelectrolytes are solid‐state electrolytes that simultaneously comprise immobile polymer‐bound ions and mobile counterions. However, certain systems remain partially dependent on solvents for ionic transport, which may limit long‐term operational stability. Moreover, the transport of only one dominant ionic species imposes constraints on channel material selection [162]. Recently, studies have reported vertical OECTs incorporating solid polymer electrolytes, in which the molecular weight of the electrolyte influences ionic transport dynamics and synaptic plasticity (Figure 23a,b) [163]. These findings suggest that the molecular design of polyelectrolytes represents a key materials strategy for regulating learning dynamics in OECT‐based neuromorphic systems.

On the other hand, eutectogels have emerged as low‐cost and environmentally friendly alternative gel electrolytes. Similar to ionogels, eutectogels consist of a polymer matrix incorporating a liquid ionic conductor. However, they are based on deep eutectic solvents (DESs). DESs are typically formed through the combination of a salt‐based hydrogen bond acceptor and a hydrogen bond donor, exhibiting low vapor pressure, high ionic conductivity, favorable thermal stability, and relatively low toxicity [162]. In studies employing DES‐based eutectogels as gate electrolytes, ultra‐flexible OECTs have been demonstrated (Figure 23c) [164]. By adjusting the ionic liquid (IL) content within the DES‐based eutectogel, doping–dedoping kinetics and hysteresis behavior can be systematically modulated, indicating that electrolyte compositional design directly influences device operation characteristics (Figure 23d). Higher IL content increases ion mobility and facilitates more efficient volumetric electrochemical doping of the P(g2T‐T) channel, resulting in higher drain current and transconductance, whereas pure DES systems exhibit lower ionic conductivity and larger hysteresis.

Collectively, polymer electrolytes and eutectogel‐based systems provide structural stability while supporting precise patterning and independent gating of individual devices. Therefore, these electrolyte platforms extend beyond simple ion‐transport layers and constitute key architectural elements closely associated with manufacturing strategies in neuromorphic and integrated OECT systems.

### 3.5 Manufacturing‐Enabled Tuning of Operating Mechanisms

The device architecture of OECTs is strongly dependent on the employed manufacturing strategy, which directly determines the geometry, thickness, and footprint of each component. Conventional microfabrication techniques have been primarily optimized for the advancement of thin‐film transistors (TFTs), focusing on the formation of ultrathin semiconducting layers. This processing paradigm emphasizes two‐dimensional, surface‐to‐surface interfaces. In contrast, OECTs operate through volumetric electrochemical reactions within the channel material. Therefore, sufficient channel thickness and a three‐dimensional network structure are essential. In addition, seamless integration with biological systems requires devices to be fabricated on uneven and mechanically compliant surfaces. To address these requirements, printing‐based manufacturing approaches have been introduced. These strategies function as active design parameters that tune the operating mechanisms of OECTs, rather than serving merely as fabrication tools. Specifically, manufacturing techniques enable precise control over channel geometry, thickness, ion diffusion length, and temporal response. By modulating these structural parameters, manufacturing directly influences ion transport pathways and volumetric doping kinetics, ultimately serving as a key factor in tuning the operating mechanisms of OECTs.

No single manufacturing strategy currently satisfies all requirements for high‐performance neuromorphic OECT systems, and the choice of fabrication method must be guided by the targeted application, device architecture, and production scale rather than resolution alone. Photolithographic approaches offer the highest patterning precision and device uniformity, enabling dense array integration and reproducible channel definition. However, their reliance on rigid substrates, photoresist compatibility constraints, and multistep processing limits applicability to flexible biointerfaces and increases fabrication cost at scale. From a neuromorphic performance standpoint, photolithographically defined thin channels favor fast ionic dynamics and volatile STP, whereas thicker channels realized by printing‐based methods support deeper ion penetration, longer retention, and more pronounced long‐term potentiation, directly linking fabrication choice to the volatile–nonvolatile balance. Printing‐based methods, including inkjet, aerosol jet, and screen printing, provide greater material versatility, substrate compatibility, and lower manufacturing cost, but limitations in feature resolution, registration accuracy, and droplet‐to‐droplet variability can introduce device‐to‐device inconsistency in densely integrated circuits. Vertical and three‐dimensional architectures offer additional design freedom by partially decoupling device footprint from volumetric ionic capacity, although fabrication complexity currently remains a barrier to large‐scale implementation. As a result, scalability is determined not only by achievable resolution, but also by the ability to preserve volumetric electrochemical functionality across increasingly dense device arrays.

It is important to note that the impact of manufacturing on OECT operation should be viewed differently from the traditional microelectronic perspective, where high resolution, precise uniformity, and minimal device‐to‐device variation are prioritized. In contrast, analog responses that resemble biological neural networks can be beneficial in neuromorphic systems. From this viewpoint, certain structural trade‐offs arising from AM may provide functional advantages rather than limitations. Although device‐to‐device variability is generally undesirable for deterministic circuit operation, controlled variability can be exploited in specific neuromorphic paradigms such as stochastic computing and reservoir computing. Similarly, unlike conventional microfabrication, soft geometries achieved through printing‐based manufacturing can become a distinct advantage in bioelectronic systems, where conformal interfacing with biological tissues is crucial. Moreover, the analog heterogeneity that naturally arises from AM can mimic the diversity observed in biological neural networks, enrich the dynamic responses of neuromorphic systems, and enhance their capacity for information representation.

A deeper challenge is that manufacturing parameters do not merely define device geometry; they directly encode neuromorphic function. Channel thickness governs ionic time constants and the balance between volatile and non‐volatile behavior; electrolyte geometry determines inter‐device crosstalk and the accessible conductance modulation range, and encapsulation strategies determine long‐term electrochemical stability under physiological conditions. This tight coupling between processing and function means that manufacturing cannot be treated as a downstream step but must instead be co‐optimized with material selection and device architecture from the outset. Future fabrication strategies will therefore likely converge on hybrid workflows, for instance, combining lithographic high‐resolution patterning for channel and electrode definition with inkjet or aerosol jet printing for electrolyte integration and three‐dimensional architectural control, enabling simultaneous optimization of ionic transport, device density, reproducibility, and system‐level integration beyond what any individual fabrication approach can currently achieve.

## 4 OECT‐Based Neuromorphic Bioelectronic Applications

The materials–architecture–manufacturing framework discussed in the preceding sections establishes that neuromorphic behavior in OECT systems emerges from geometry‐programmed ionic–electronic dynamics rather than from material composition alone. By regulating channel thickness, electrolyte configuration, and fabrication strategy, ionic penetration depth, volumetric capacitance, and relaxation kinetics can be deliberately tuned, thereby encoding temporal response, retention, and nonlinear adaptation at the device level. When such electrochemical dynamics are organized across increasing levels of structural and functional complexity, OECT platforms naturally extend beyond isolated synaptic elements toward hierarchical neuromorphic systems.

Within this context, OECTs and related OMIECs uniquely bridge biological ionic signaling and electronic computation through volumetric ion–electron coupling, enabling bioinspired neuromorphic operation in soft and hydrated environments [11]. Unlike conventional solid‐state synaptic devices that rely primarily on electronic band transport, OECT‐based platforms intrinsically operate within electrolyte‐mediated contexts, where ionic flux and electrochemical doping dynamically modulate channel conductance. This fundamental compatibility with biological ionic signaling renders OECT architectures particularly suitable for chemical sensing, physiological interfacing, and biohybrid neuromorphic systems [165].

Building upon these material and fabrication principles, OECT‐based neuromorphic applications can be organized according to a functional hierarchy that reflects the progressive integration of electrochemical device physics into system‐level intelligence. This hierarchy spans: (i) neurotransmitter‐driven synaptic encoding and chemically mediated plasticity, (ii) in‐sensor and multimodal neuromorphic processing for edge‐level signal transformation, (iii) device‐ and network‐level learning architectures enabled by material‐ and structure‐encoded dynamics, (iv) closed‐loop bioelectronic systems integrating sensing, computation, and adaptive actuation, and (v) perception‐level biohybrid intelligence coupling neuromorphic encoding with embodied sensory–motor functions.

Through this progression, OECT platforms evolve from material‐level ionic–electronic interactions to distributed, adaptive intelligence, demonstrating how electrochemical transport physics can be hierarchically organized into biointegrated neuromorphic architectures.

### 4.1 Neurotransmitter‐Driven Synaptic Encoding

#### 4.1.1 Neurotransmitter‐Induced Short‐Term and Long‐Term Plasticity

In this context, electroactive neurotransmitters act as chemical write operations that convert transient ionic gating into persistent or history‐dependent weight updates [166]. This dynamic encoding strategy was further extended to selectively discriminate dopamine from structurally similar metabolites by exploiting plasticity‐mediated temporal signatures instead of equilibrium electrochemical metrics [167].

Building on this foundation, dopamine‐mediated plasticity was realized in mechanically compliant and fully printed PEDOT:PSS‐based OECT platforms, confirming both structural robustness and fabrication scalability for biohybrid integration [168, 169]. In flexible PEDOT:PSS‐based OECT artificial synapses, dopamine oxidation at the gate generates protons that become trapped within the polymer matrix, converting reversible ionic gating into persistent volumetric dedoping and thereby transforming STP into long‐term synaptic weight modulation [168]. This concept was further demonstrated in fully printed OECT artificial synapses, where dopamine‐mediated oxidation produces persistent inhibitory PSC responses and cumulative conductance changes, enabling LTP‐like behavior governed by neurotransmitter concentration and stimulation history [169].

**FIGURE 24:**
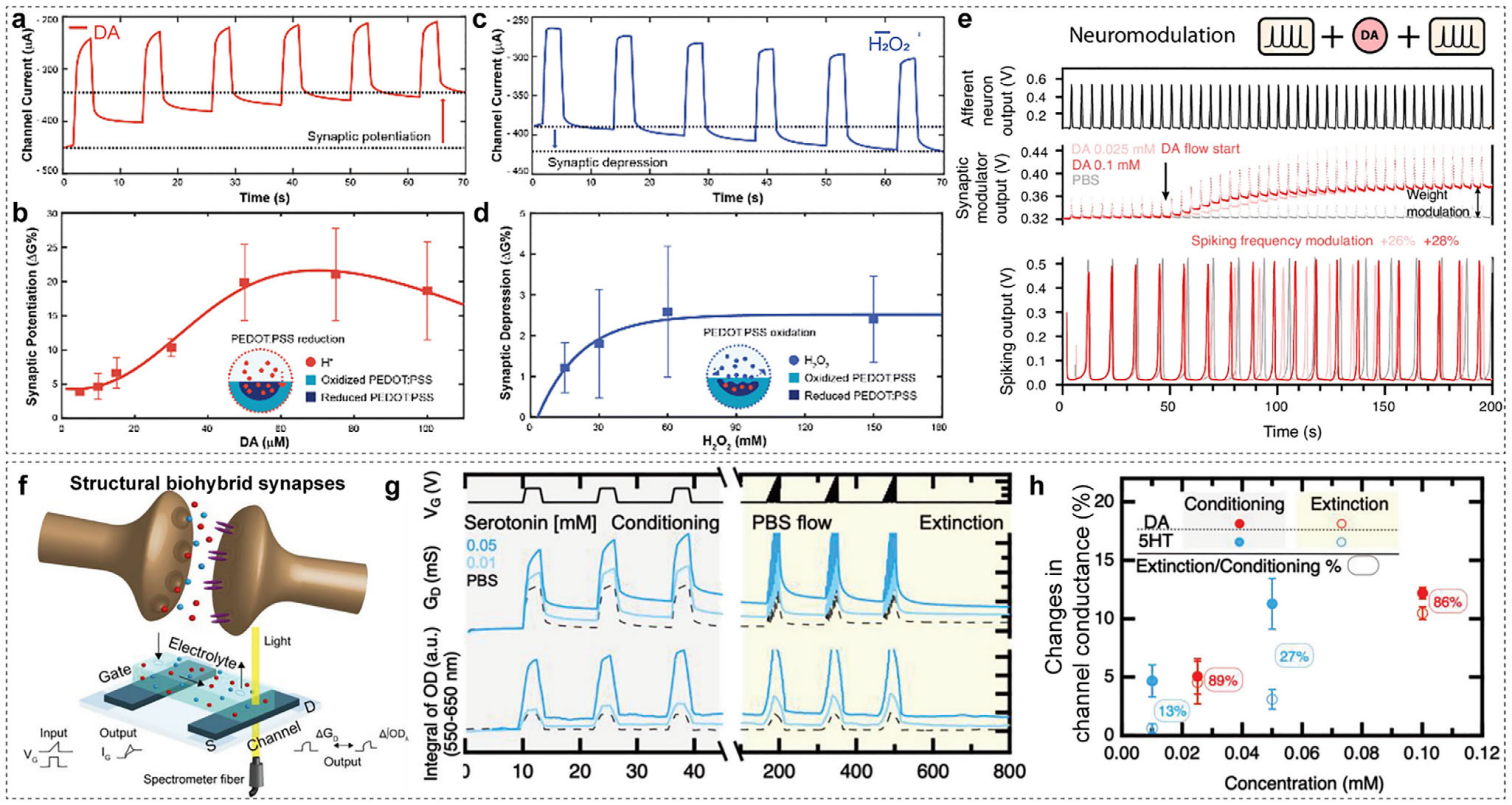
Dopamine (DA)‐induced synaptic potentiation (a) and its concentration dependence (b), arising from proton‐assisted reduction and dedoping of the PEDOT:PSS channel. H_2_O_2_‐induced synaptic depression (c) and corresponding concentration dependence (d) through oxidation of PEDOT:PSS. Reproduced under the terms of the CC‐BY license [170]. Copyright 2024, The Authors, published by Royal Society of Chemistry (RSC). (e) Dopamine‐regulated neuromodulation in a spiking circuit, where neurotransmitter‐driven conductance changes modulate interneuron spike frequency. Reproduced under the terms of the CC‐BY license [171]. Copyright 2024, The Authors, published by Springer Nature. (f) Fluidically integrated synapse platform. (g) Conditioning and extinction dynamics. (h) Neurotransmitter‐dependent recovery behavior. Reproduced under the terms of the CC‐BY license [172]. Copyright 2023, The Authors, published by Wiley‐VCH.

A representative implementation of neurotransmitter‐driven synaptic modulation was demonstrated in an electrochemical neuromorphic device (ENODe) platform in which redox‐active biochemical species directly regulate the conductance state of the PEDOT:PSS channel through faradaic reactions [170]. As shown in Figure 24a, the application of repeated square voltage pulses at the gate in the presence of dopamine (DA) produces progressive baseline shifts in the channel current, corresponding to cumulative synaptic potentiation. Mechanistically, dopamine oxidation at the gate electrode generates protons that penetrate the PEDOT:PSS bulk and bind with the sulfonate groups of PSS^−^, resulting in volumetric reduction of PEDOT and persistent dedoping of the conducting polymer channel (schematically illustrated in Figure 24b). The resulting decrease in channel conductance represents a non‐volatile update of synaptic weight analogous to long‐term synaptic potentiation in biological systems. Importantly, the magnitude of potentiation scales with dopamine concentration, exhibiting an approximately linear dependence within the physiologically relevant range (5–30 µm) before approaching saturation at higher concentrations due to limited oxidative species at the gate interface (Figure 24b). Complementary synaptic depression can be achieved by introducing oxidizing agents such as hydrogen peroxide (H_2_O_2_), which reverse the dopamine‐induced reduction of PEDOT:PSS. When voltage pulses are applied in the presence of H_2_O_2_, the PEDOT:PSS channel undergoes oxidation, gradually restoring its initial conductance state (Figure 24c). The corresponding dependence of synaptic depression on H_2_O_2_ concentration is summarized in Figure 24d, where the conductance recovery increases slightly with increasing oxidant concentration but rapidly reaches a plateau. This behavior suggests that the oxidation process primarily occurs at the polymer surface rather than through bulk ion penetration, in contrast to the volumetric reduction induced by dopamine [170]. Together, these complementary redox reactions enable reversible tuning of synaptic weights via chemical inputs, establishing a biochemical analogue of potentiation and depression in organic neuromorphic devices.

Beyond isolated plasticity events, neurotransmitter‐mediated conductance modulation can also regulate spiking neural circuits through neuromodulatory feedback pathways. As demonstrated in Figure 24e, the ENODe‐based synaptic modulator was interfaced with a spiking neuron circuit that emulates afferent neural firing. When the spike voltage amplitude exceeds the oxidation potential of dopamine (*V* ≈ 0.3 V), oxidation reactions are triggered at the gate electrode, inducing non‐volatile conductance changes in the OECT channel. This modulation shifts the baseline output voltage of the synaptic modulator, effectively altering the synaptic weight that controls the firing frequency of the downstream neuron. Consequently, increasing dopamine concentration leads to a gradual rise in interneuron spiking frequency, with experimentally observed increases of approximately 26% and 28% over the PBS baseline at dopamine concentrations of 0.025 and 0.1 mm, respectively [171]. Such neurotransmitter‐dependent spike‐rate modulation closely resembles biological neuromodulation mechanisms, in which dopamine dynamically regulates synaptic strength during learning and reward‐based adaptation.

Collectively, these studies establish OECTs as chemical‐to‐synaptic transducers, in which neurotransmitter redox reactions directly modulate channel conductance through coupled ionic–electronic processes. This mechanism enables neuromorphic devices to translate biochemical signals into persistent synaptic weight updates, providing a scalable and mechanically compliant platform for biohybrid neuromorphic interfaces.

#### 4.1.2 Multi‐Gate and Multi‐Modal Chemical Synaptic Architectures

While single‐neurotransmitter systems emulate elementary plasticity, biological synapses integrate multiple presynaptic inputs and co‐released neuromodulators. To approximate this functionality, dual‐gate and multimodal OECT architectures have been developed. Dual‐gate OECT architectures emulate the convergence of multiple presynaptic inputs by integrating two independent gate electrodes on a shared channel–electrolyte system, where reversible Na^+^‐mediated gating enables symmetric multi‐input responses and dopamine oxidation introduces persistent conductance suppression for neurotransmitter‐driven multi‐presynaptic integration [169].

Beyond multi‐gate electrical integration, multimodal electrochemical neuromorphic platforms have incorporated fluidic neurotransmitter delivery to emulate co‐modulation and synaptic recycling processes. As shown in Figure 24f, the artificial synapse architecture integrates a fluidic module with an electrochemical channel, mapping presynaptic neurotransmitter release and postsynaptic conductance modulation onto a single device framework. Periodic gate voltage pulses trigger oxidation of serotonin (5‐HT), inducing distinct conductance modulation dynamics (Figure 24g). Time‐resolved measurements reveal neurotransmitter‐specific conditioning and extinction behavior under controlled PBS flow. Quantitative analysis in Figure 24h shows serotonin produces more persistent conductance changes (<27% recovery), whereas dopamine‐induced modulation is largely semi‐reversible (>80% recovery), indicating neurotransmitter‐dependent memory retention and forgetting dynamics [172].

Collectively, these multi‐gate and multimodal architectures move beyond single‐input chemical sensing toward integrated synaptic computation, where spatially distributed electrical inputs and chemically distinct neuromodulators cooperatively regulate synaptic weight evolution. Such systems represent an important step toward biohybrid neuromorphic interfaces capable of simultaneously processing electrical, chemical, and biochemical signals within soft and physiologically compatible environments.

Although numerous OECT‐based artificial synapses and memory devices have been reported, direct comparison remains challenging because key performance metrics are often distributed across different studies and device architectures. To provide a quantitative benchmarking perspective, Table 2 summarizes representative OECT‐based neuromorphic devices in terms of operating voltage, retention time, conductance states, energy consumption per event, and weight‐update linearity. These metrics provide a practical framework for evaluating synaptic performance and identifying device‐level trade‐offs across different neuromorphic OECT platforms.

**TABLE 2:** Representative performance parameters of OECT‐based neuromorphic devices.

Device platform	Channel material	Device architecture	Operating voltage (V)	Retention time	Conductance states	Energy per event	Weight update linearity (NL)	Refs.
ENODe	PEDOT:PSS/PEDOT:PSS‐PEI	Planar electrochemical synapse	∼1 V operating range	>25 h	>500	<10 pJ for 10^3^ µm^2^	Highly linear (NL N/R)	[32]
OECT artificial synapse	PEDOT:PSS	Planar OECT, aqueous KCl electrolyte	±0.5	STP (∼200–250 ms)	N/R	N/R	N/R	[31]
Three‐terminal organic neuromorphic device	PEDOT:PSS	Three‐terminal electrolyte‐gated organic transistor	VGS = −0.5–0 V; VDS pulse = −0.5 V	Tunable STP	N/R	N/R	N/R	[67]
Organic electrochemical synaptic transistor (OEST)	PIBET‐AO (p‐type)	Solid‐state OEST with semi‐solid ionic liquid gel	<1 V operation (±1.5 V programming)	82% retention after 1000 s	>200	N/R	3.5 (long‐term potentiation), 2.63 (LTD)	[68]
Fully 3D‐printed OECT artificial synapse	PEDOT:PSS	Fully extrusion‐printed depletion‐mode OECT with printed PSSNa hydrogel electrolyte	*VG* = −0.4–−0.8 V	∼1 h	25	N/R	N/R	[136]
3D‐printed intrinsically stretchable OECT synapse array	PEDOT:PSS/ Glycero	Fully 3D‐printed intrinsically stretchable depletion‐mode OECT with PAAm ionic hydrogel electrolyte	*VG* = ±0.5 V; VD = −0.5 V	N/R	N/R	N/R	N/R	[137]
Hybrid 3D/inkjet‐printed neuromorphic OECT	PEDOT:PSS	Hybrid FDM/inkjet‐printed depletion‐mode OECT with PBS electrolyte	*VG* = 0–0.8 V	STP memory up to ∼80 s	N/R	N/R	N/R	[141]
cv‐OECT for multi‐modal sensing, memory, and processing	PTBT‐p	Vertical traverse OECT with crystalline–amorphous OMIEC channel and ion‐gel electrolyte	Volatile: −0.7 V; non‐volatile: >0.8 V (typically −1.5 V)	>10 000 s	1024 (10‐bit)	N/R	0.20 (long‐term potentiation), 1.63 (LTD)	[78]

### 4.2 In‐Sensor and Multimodal Neuromorphic Processing

#### 4.2.1 Single‐Device Sensing–Memory–Processing Integration

**FIGURE 25:**
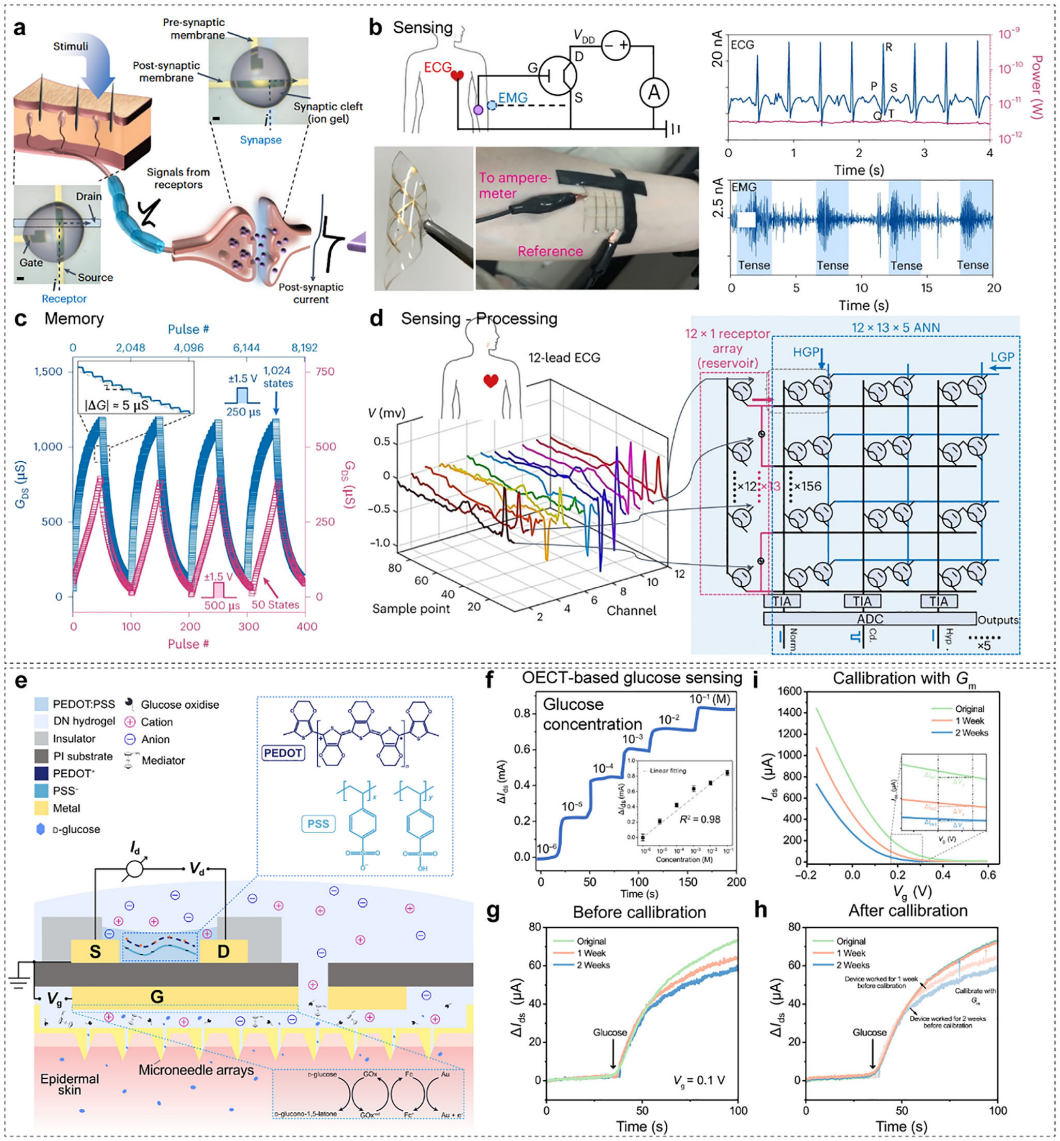
Single‐Device Sensing–Memory–Processing Integration. (a) Design of a mode‐switchable cv‐OECT capable of operating as both a volatile receptor and a non‐volatile synapse. (b) Multi‐modal volatile sensing of physiological signals (e.g., ECG/EMG) using flexible cv‐OECT devices. (c) Voltage‐controlled LTP in cv‐OECTs demonstrates nonvolatile analog synaptic behavior with up to 1024 conductance states and low switching stochasticity. Blue and pink boxes indicate 1024 and 50 analog states, respectively. (d) Homogeneous device integration enabling reservoir computing and real‐time ECG classification. Reproduced with permission [78]. Copyright 2023, Springer Nature. (e) Schematic of the OECT‐based glucose sensor and its enzymatic signal transduction mechanism. (f) Real‐time current response of the OECT sensor to varying glucose concentrations. (g) Degradation of sensing response after prolonged usage due to reduced transconductance (*g*_m_). (h) Recovery of sensing performance after *g*_m_ normalization. (i) Self‐calibration strategy based on extracting and normalizing *g*_m_ from transfer characteristics. Reproduced under terms of the CC‐BY license [173]. Copyright 2024, The Authors, published by AAAS.

The coexistence of nonlinear dynamics and fading memory in ionic–electronic coupling enables OECT devices to simultaneously perform sensing, memory, and computation within a single physical platform. In particular, vertically structured mode‐switchable crystalline–amorphous OECTs (cv‐OECTs) provide a representative example of this integration (Figure 25a) [78]. The mixed OMIEC channel allows for reconfigurable operation between a volatile sensing receptor and a non‐volatile analogue synapse.

In the volatile regime, ionic doping occurs primarily within amorphous domains of the channel, enabling rapid ion transport and reversible conductance modulation. Under these conditions, the device operates as a sensitive receptor capable of multimodal physiological sensing, including direct electrophysiological signal acquisition such as electrocardiography (ECG) and electromyography (EMG) under ultralow power operation (Figure 25b). At higher gate potentials, ions become partially trapped within crystalline domains, producing stable electrochemical doping states and long retention times. This regime enables analogue synaptic functionality, where voltage‐controlled long‐term potentiation produces up to 1024 programmable conductance levels (Figure 25c). When multiple identical devices are integrated into arrays, the intrinsic nonlinear dynamics and memory effects enable reservoir computing architectures capable of real‐time ECG signal classification, demonstrating fused sensing–memory–processing within a unified material system (Figure 25d).

Extending this concept to wearable biochemical systems, a fully integrated OECT‐based glucose monitoring platform demonstrates how ionic–electronic coupling enables hardware‐level signal amplification and adaptive sensing. In this configuration, enzymatic glucose reactions induce small gate potential variations that are amplified through transconductance (*g*_m_) into large channel current responses (Figure 25e,f), significantly improving signal‐to‐noise ratio. Although prolonged use leads to gradual sensitivity drift due to *g*_m_ variation (Figure 25g), a self‐calibration strategy based on *g*_m_ normalization restores sensing accuracy and stability (Figure 25h,i) [173].

Together, these studies highlight a unifying principle of OECT‐based systems in which the coupled ionic and electronic dynamics of OMIECs enable dynamic transitions between transient sensing, persistent analogue memory, and hardware‐level signal transformation within a single material platform. In such architectures, ionic penetration, trapping, and transconductance modulation collectively serve as physical mechanisms for perception, weighting, and adaptive calibration. These demonstrations establish OECTs not merely as bioelectronic sensors or artificial synapses, but as intrinsic sensing–memory–processing nodes, representing a critical architectural shift toward in‐sensor computing and hardware‐efficient neuromorphic biointerfaces.

#### 4.2.2 Flexible and Wearable in‐Sensor Computing Platforms

Mechanically compliant in‐sensor neuromorphic preprocessing is increasingly being realized in flexible and wearable OECT platforms, where signal acquisition, analogue conditioning, and early‐stage computation are co‐localized directly at the body interface. Rather than miniaturizing conventional processors onto flexible substrates, these systems implement edge intelligence at the material level by exploiting ionic–electronic coupling within OECT arrays to perform nonlinear transformation and temporal projection intrinsically within the sensing hardware. In this paradigm, transistor arrays simultaneously function as transducers, dynamic processors, and reservoir layers within soft biointegrated systems.

**FIGURE 26:**
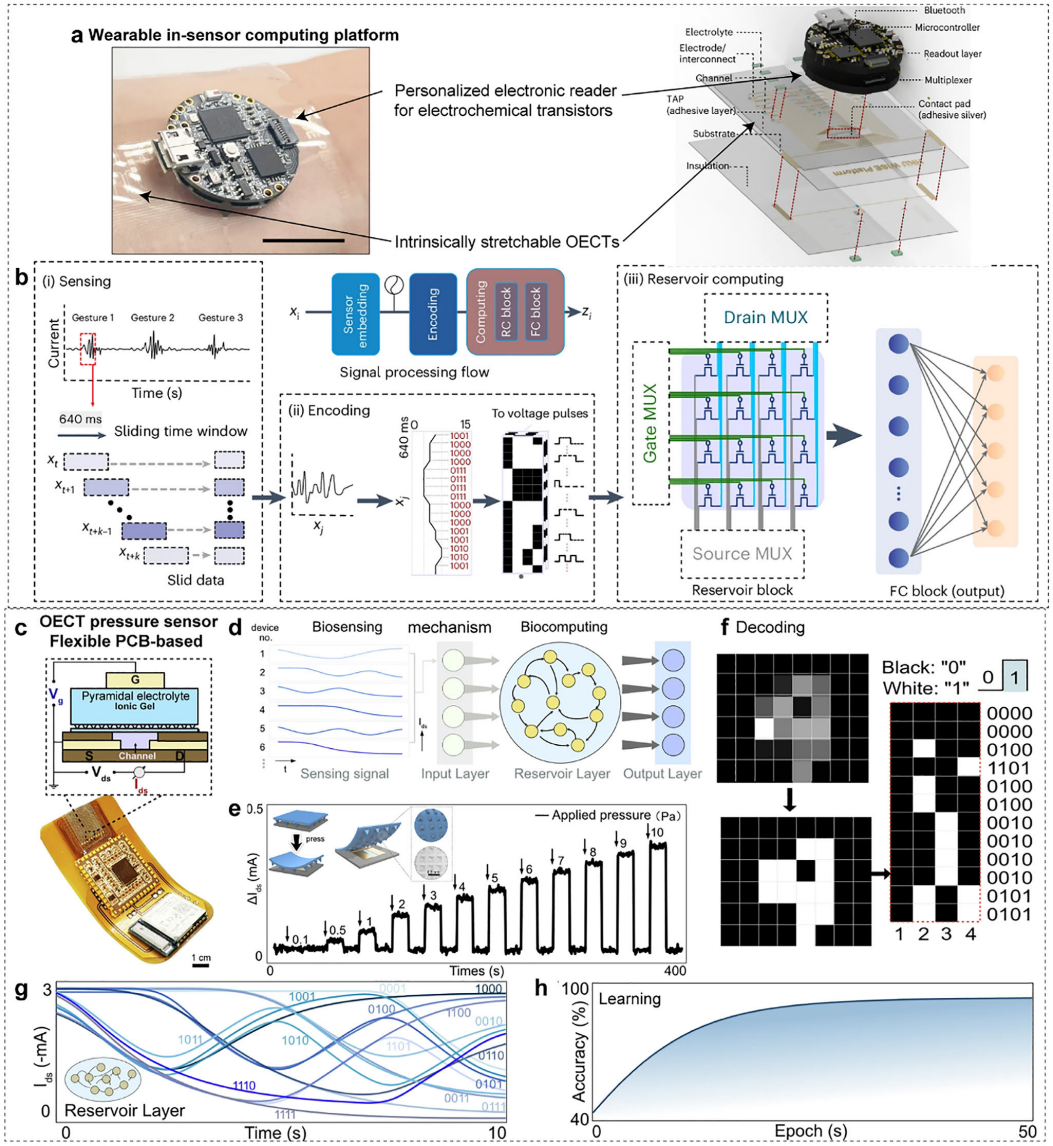
Flexible and wearable OECT‐based in‐sensor computing platforms. (a) Photograph and schematic of the stretchable ISOECT‐based wearable in‐sensor computing system. (b) EMG‐based gesture recognition workflow with sliding‐window encoding and reservoir classification. Reproduced with permission [154]. Copyright 2024, Springer Nature. (c) Flexible PCB‐based gel‐gated OECT sensor array. (d) Sensing and ion‐modulated computing mechanism. (e) Drain current responses to pressure stimuli. (f) Reservoir computing decoding results. (g) Transient *I_DS_* responses to binary gate patterns. (h) Training accuracy evolution (>90%). Reproduced under terms of the CC‐BY‐NC‐ND license [160]. Copyright 2025, The Authors, published by Springer Nature.

A representative stretchable wearable system integrates intrinsically stretchable OECT (ISOECT) arrays with compact readout electronics to establish a fully wearable in‐sensor computing framework (Figure 26a) [154]. EMG signals are detected and amplified at their origin, segmented using a 640 ms sliding window with a 40 ms stride, and encoded into four‐bit sequential voltage pulse streams (0000–1111). These pulse sequences are directly applied as gate inputs to a 4 × 4 ISOECT reservoir array, where nonlinear transient ionic–electronic coupling generates distinguishable drain‐current (*I_DS_*) states that are subsequently classified through a fully connected output layer. Importantly, the preservation of nonlinear reservoir dynamics under substantial mechanical deformation demonstrates that neuromorphic functionality can be maintained even under stretch (Figure 26b). In this context, mechanical compliance serves not only as a packaging attribute but also as a functional design constraint under which computational fidelity is preserved.

Complementing stretchability‐driven robustness, fully integrated flexible in‐sensor circuits fabricated on flexible printed circuit board (fPCB) substrates demonstrate scalable system‐level integration suitable for manufacturable wearable architectures (Figure 26c–h) [160]. In Figure 26c, a gel‐gated OECT pressure sensor array is monolithically integrated with flexible circuitry, where the gel electrolyte simultaneously serves as an ionic medium and gating interface, enabling stable electrochemical modulation of the PEDOT:PSS channel while maintaining mechanical compliance. Figure 26d illustrates the sensing‐to‐computing workflow enabled by this architecture, where mechanical pressure stimuli are transduced into electrical signals and encoded into transient current responses that serve as inputs for reservoir computing. The sensing characteristics are shown in Figure 26e, where reproducible modulation of the drain current under sequential pressure inputs establishes the physical signals used for neuromorphic processing. Building on this sensing layer, Figure 26f presents the decoding stage of the reservoir computing pipeline, where spatiotemporal current patterns generated by the OECT network are mapped through a trained readout layer to produce classified outputs. The reservoir dynamics are further illustrated in Figure 26g, where binary gate voltage patterns (0 and 0.4 V) generate multiple distinguishable transient *I_DS_* states, effectively expanding the dimensionality of the input signal for temporal feature extraction. Finally, Figure 26h shows that the system rapidly achieves >90% classification accuracy within approximately 50 training epochs, demonstrating reliable edge‐level neuromorphic computation in flexible wearable formats.

While these platforms are built on different material compositions and mechanical strategies, including intrinsically stretchable ISOECT arrays and gel‐gated OECT circuits integrated with flexible PCBs, they converge on a shared principle that ionic dynamics at soft interfaces simultaneously facilitate sensing and information processing. This represents a structural departure from the conventional architecture consisting of separate sensors and external processors, replacing rigid functional partitioning with distributed neuromorphic intelligence embedded directly within materials. Such developments point toward wearable bioelectronic systems in which computation is no longer added to sensing modules but becomes physically inseparable from the sensing substrate itself.

The efficiency of OECT‐based in‐sensor computing platforms depends strongly on the interplay between ionic transport and device geometry. Channel thickness and electrolyte confinement determine the characteristic timescale of nonlinear signal processing, whereas higher µC* generally enhances signal transduction and improves discrimination of transient conductance states. Materials with tunable volatility, such as cv‐OECTs capable of switching between volatile receptor‐like and non‐volatile synaptic modes, are particularly attractive for in‐sensor computing. By co‐localizing sensing, memory, and preprocessing within a single device, these platforms reduce the area and energy overhead associated with separate sensing and memory elements, representing a key architectural advantage over conventional edge‐computing approaches.

### 4.3 Device‐ and Network‐Level Learning Architectures

#### 4.3.1 Material‐ and Structure‐Engineered Synaptic Plasticity

At the device level, OECT‐based neuromorphic systems increasingly move beyond isolated synaptic emulation toward learning architectures in which plasticity is intrinsically governed by engineered material composition and structural topology. In contrast to neurotransmitter‐triggered modulation (Section 4.1), plasticity in this context arises from controlled ionic transport pathways, electrochemical doping kinetics, and morphology‐dependent charge retention mechanisms. Here, learning behavior is not externally programmed at the circuit level, but physically encoded within the material state and geometry of the device itself.

**FIGURE 27:**
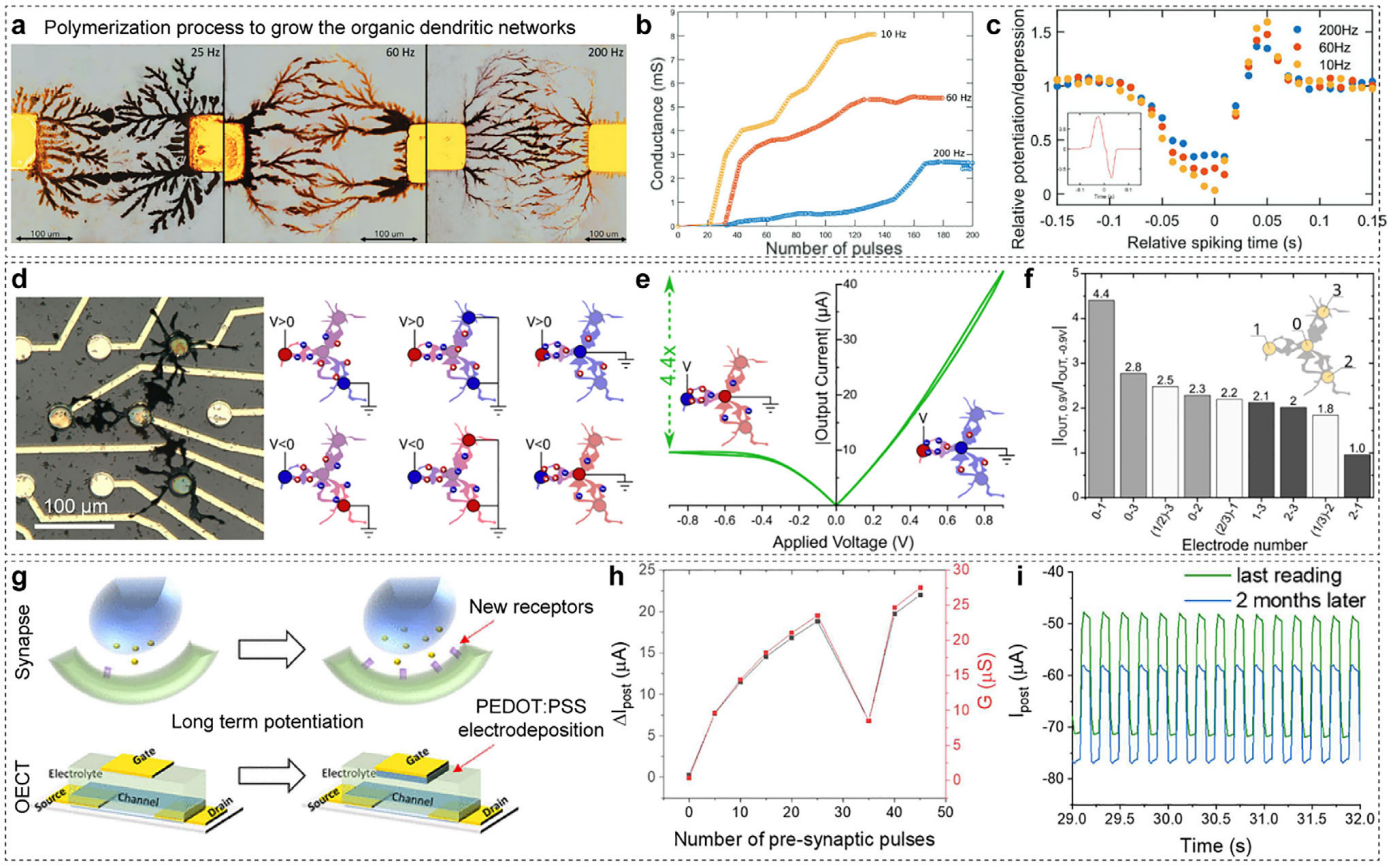
Material‐ and structure‐encoded synaptic plasticity in dendritic OECTs. (a) Frequency‐controlled dendritic growth via AC electropolymerization. (b) Growth‐dependent LTP‐like conductance reinforcement. (c) Morphology‐dependent STDP characteristics. Reproduced under the terms of the CC‐BY‐NC license [175]. Copyright 2021, The Authors, published by Wiley‐VCH. (d) Y‐shaped dendritic OECT architecture enabling topology‐driven ion redistribution. (e) Configuration‐dependent asymmetric current response and self‐gating behavior. (f) Topology‐encoded nonlinear and rectifying characteristics. Reproduced under the terms of the CC‐BY license [176]. Copyright 2025, The Authors, published by Wiley‐VCH. (g) Gate‐side PEDOT:PSS growth induced by repeated stimulation. (h) Pulse‐number‐dependent potentiation behavior. (i) Long‐term retention enabled by growth‐mediated structural stabilization. Reproduced under the terms of the CC‐BY license [179]. Copyright 2023, The Authors, published by ACS.

Electropolymerization‐driven growth of dendritic OECT channels provides a clear demonstration of morphology‐encoded learning. Early work on organic dendritic networks grown by electropolymerization showed that three‐dimensional branched morphologies introduce spatially heterogeneous ionic accessibility and distributed electrochemical modulation within the channel [174]. AC‐electropolymerization enables direct control over dendritic branching density and fiber thickness, thereby modulating ionic diffusion length scales and interfacial surface area (Figure 27a). Increasing the polymerization frequency yields thinner and more highly branched networks, effectively reshaping ionic accessibility at the electrolyte–channel interface. These morphology‐induced modifications alter the effective electrochemical time constants of the device, resulting in programmable conductance reinforcement under repeated stimulation (Figure 27b). Importantly, the spike‐timing‐dependent plasticity (STDP) window depends strongly on the growth frequency (Figure 27c), indicating that learning rules emerge directly from morphology‐dependent ionic kinetics [175]. In such dendritic OECT systems, synaptic plasticity is structurally encoded, where fabrication parameters define geometry, geometry defines ionic dynamics, and ionic dynamics define the learning function.

Beyond single‐branch architectures, brain‐inspired dendritic polymer networks further highlight how structural topology governs nonlinear signal integration in multiterminal OECT systems [176]. In Y‐shaped dendritic configurations, the spatial arrangement of interconnected polymer branches redistributes mobile ions in a configuration‐dependent manner (Figure 27d), leading to asymmetric current responses and self‐gating behavior (Figure 27e). Selective polarization induces branch‐specific dedoping, producing rectifying characteristics with pronounced asymmetry ratios (Figure 27f). These nonlinear and hysteretic responses arise not from discrete circuit components but from topology‐defined ionic cross‐talk and distributed capacitance within the dendritic network. Such structure‐encoded nonlinear integration represents a hardware‐level learning primitive that bridges device‐level plasticity with network‐level computation.

In addition to morphological control, intrinsic material parameters and structural evolution provide complementary axes for tuning synaptic behavior. Charge‐density engineering of PEDOT:PSS has demonstrated that volumetric capacitance, ionic penetration depth, and electrochemical doping efficiency can be systematically modulated, thereby influencing short‐ and long‐term potentiation characteristics through adjustments to the internal electrochemical environment of the polymer [177]. Evolvable OECT architectures extend this concept by enabling in situ electrochemical modification of the channel during operation, coupling conductance updates with gradual structural reconfiguration of the active material [178]. A particularly illustrative example is gate‐side electrochemical polymerization of PEDOT:PSS, in which synaptic potentiation is reinforced through growth‐mediated structural modification rather than solely through reversible ionic doping [179]. In these architectures, repeated presynaptic stimulation induces controlled interfacial polymer growth at the gate/channel interface (Figure 27g), progressively reshaping the local electrochemical landscape. This growth process produces cumulative conductance enhancement as a function of training pulses (Figure 27h), exhibiting long‐term potentiation behavior governed directly by stimulation history. Crucially, the potentiated states exhibit markedly improved retention over extended time scales (Figure 27i), in contrast to conventional doping‐based OECT plasticity that relies on transient ionic dedoping. Here, memory is embedded within the evolving material structure itself, demonstrating that synaptic weight can be physically stabilized through controlled material growth.

Collectively, these studies establish a unified design paradigm for OECT‐based neuromorphic hardware in which plasticity is co‐determined by morphology, topology, charge density, and material evolution. Rather than representing a purely transient electrical phenomenon, synaptic behavior increasingly reflects the underlying physical state of the device and its electrochemical history. By directly coupling fabrication conditions, structural geometry, and ionic–electronic transport dynamics, material‐ and structure‐engineered strategies provide a pathway toward neuromorphic systems in which learning rules are intrinsically embedded in matter.

#### 4.3.2 Artificial Neurons and Spiking Dynamics

Beyond synaptic plasticity, OECT/OMIEC platforms have advanced toward artificial neurons in which spiking and oscillatory behaviors arise intrinsically from ionic–electronic coupling rather than from purely electronic threshold circuits.

**FIGURE 28:**
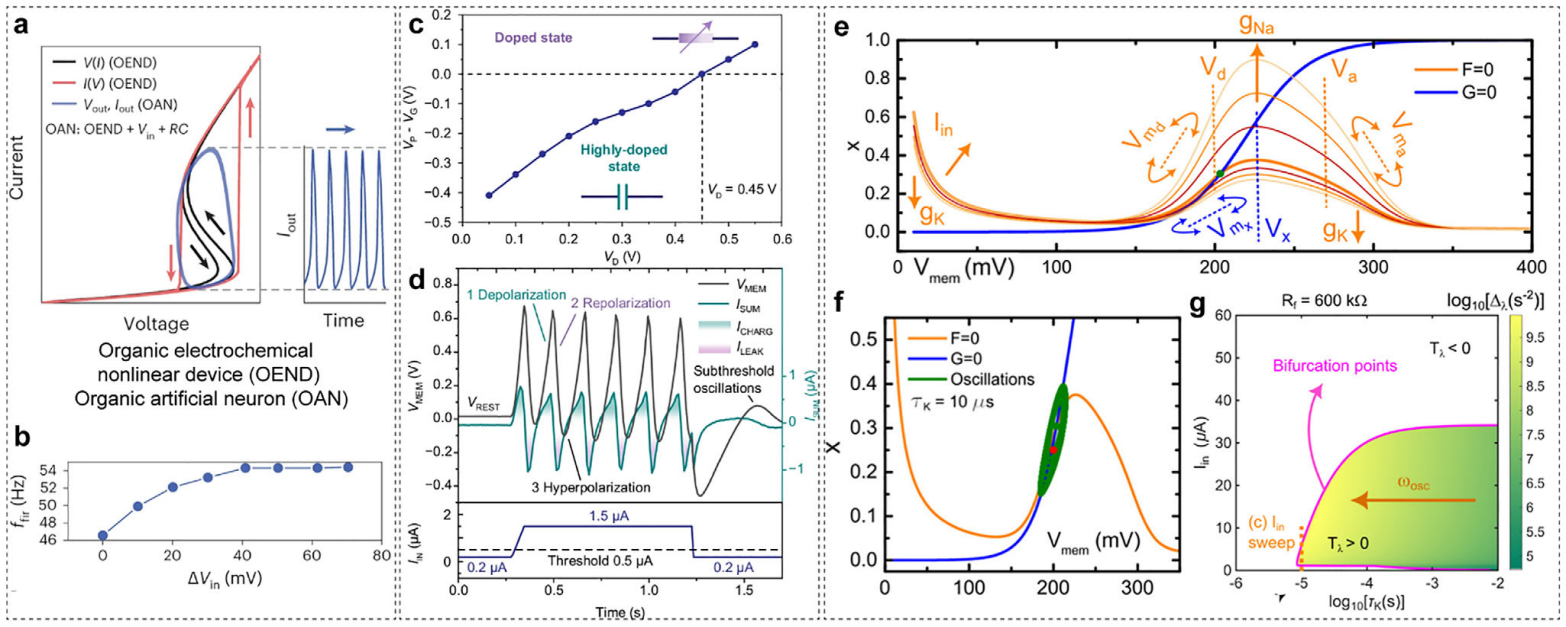
OECT‐based artificial neurons and nonlinear dynamics. (a) OEND negative differential resistance and RC‐driven oscillations. (b) Voltage‐controlled firing frequency. Reproduced under the terms of the CC‐BY license [180]. Copyright 2022, The Authors, published by Springer Nature. (c) Channel doping‐state transition near threshold. (d) Single‐transistor spike waveform (*I*_CHARG_/*I*_LEAK_). Reproduced under the terms of the CC‐BY‐NC license [183]. Copyright 2025, The Authors, published by Springer Nature. (e) Phase‐space nullclines. (f) Limit‐cycle oscillation. (g) Hopf bifurcation map. Reproduced under the terms of the CC‐BY license [185]. Copyright 2025, The Authors, published by ACS.

Early demonstrations of organic artificial neurons established that nonlinear electrochemical elements can directly generate spike‐like oscillations in aqueous environments. In the organic electrochemical negative differential resistance device (OEND), the current–voltage response exhibits an S‐shaped negative differential resistance region in the V(I) mode (Figure 28a) [180]. When integrated with an RC load to form an organic artificial neuron (OAN), this nonlinear element produces parametric oscillations, visualized as Lissajous‐like trajectories in phase space, indicating that integrate–discharge cycles originate from electrochemical state evolution rather than digital switching. The OAN further operates as a voltage‐controlled oscillator, with firing frequency increasing systematically with the input overdrive voltage Δ*V*_in_ above threshold (Figure 28b). Because ionic strength and biomolecular composition directly modulate the oscillation window, spike generation can be chemically modulated, enabling in situ biointerfacing under physiological conditions.

Device‐level implementations extended this concept through OECT‐based leaky integrate‐and‐fire (LIF) architectures, where proton accumulation and interfacial relaxation emulate membrane integration, leakage, and reset processes [181]. Unlike rate‐coded hardware neurons, these ionic systems encode information in spike timing, enabling coincidence detection and dynamic pattern recognition in noisy environments.

At the circuit level, fully printed complementary OECT‐based organic electrochemical neurons (OECNs) introduced compact Axon–Hillock architectures in which firing frequency is governed not only by electrical bias and capacitance but also by electrolyte ion concentration [182]. This ion‐mediated control axis establishes chemically tunable excitability while preserving compatibility with organic electrochemical synapses.

Subsequent work demonstrated that spiking can be embedded directly within a single electrochemical memtransistor. In BBL‐based single‐transistor (1T)–OECNs, ion‐tunable antiambipolarity and asymmetric transient responses produce hysteretic transitions between highly doped and less doped channel states (Figure 28c) [183]. When the membrane voltage exceeds a critical threshold (*V*_D_ ≳ 0.45 V at *V*_G_ = 0.4 V), delayed channel leakage current (*I*_LEAK_) activates, initiating repolarization analogous to K^+^‐channel opening. As the voltage decreases, the channel re‐enters the highly doped state, enabling capacitive recharge and spike regeneration (Figure 28d). The intrinsic interplay between charging (*I*_CHARG_) and leakage currents thus reproduces action‐potential‐like dynamics including refractory behavior and adaptation without external RC or amplifier blocks, enabling dense flexible arrays and direct sensory‐to‐spike encoding.

While the preceding architectures emphasize spike generation, computation can also emerge at the dendritic integration level. Planar artificial soma architectures integrating multiple presynaptic voltage inputs exploit electrolyte–OMIEC RC relaxation dynamics as the primary computational kernel [184]. Synaptic weights are tuned via electrolyte ionic strength, and classification is achieved through integration of the somatic current into a scalar charge output (|Q|end). These passive dendritic integrators demonstrated spatio‐temporal pattern recognition, including speech‐related tasks, illustrating how interfacial kinetics can serve as embedded low‐dimensional classifiers within soft ionic interfaces.

Beyond phenomenological demonstrations, nonlinear dynamical systems analysis provides a unified theoretical framework for OECT‐based neuron circuits. In hybrid OECT–amplifier architectures, oscillatory regimes are governed by nullcline intersections in phase space (Figure 28e,f) [185]. The balance between sodium‐like activation (*g*_Na_), potassium‐like relaxation (*g*_K_), membrane capacitance (*C*_m_), and input bias (*I*_in_) determines whether the system converges to a stable fixed point or evolves toward a limit cycle through Hopf bifurcation. Parametric bifurcation maps define the stability boundaries separating quiescent and oscillatory regimes (Figure 28g), while externally tunable parameters, including membrane capacitance, amplifier gain, and input current, enable systematic control over oscillation onset, amplitude, and frequency.

Collectively, these developments trace a progression from chemically tunable spiking elements to proton‐driven LIF neurons, ion‐modulated circuit architectures, single‐transistor electrochemical spiking devices, dendritic artificial somas, and predictive nonlinear oscillator theory. OECT platforms thus emerge not merely as synaptic mimics, but as physically grounded nonlinear neuronal systems in which computation arises directly from ionic transport and electrochemical state dynamics.

#### 4.3.3 Network Computation and Reconfigurable Learning

Extending beyond single‐device plasticity and spiking dynamics, OECT‐based systems increasingly exploit collective ionic–electronic interactions to realize distributed network‐level computation. In such architectures, computation is not primarily determined by static wiring density but instead emerges from electrolyte‐mediated coupling, nonlinear ionic transport, and temporally evolving conductance states. Consequently, connectivity, gain, and memory arise dynamically from controllable ionic motion within a shared electrochemical environment.

**FIGURE 29:**
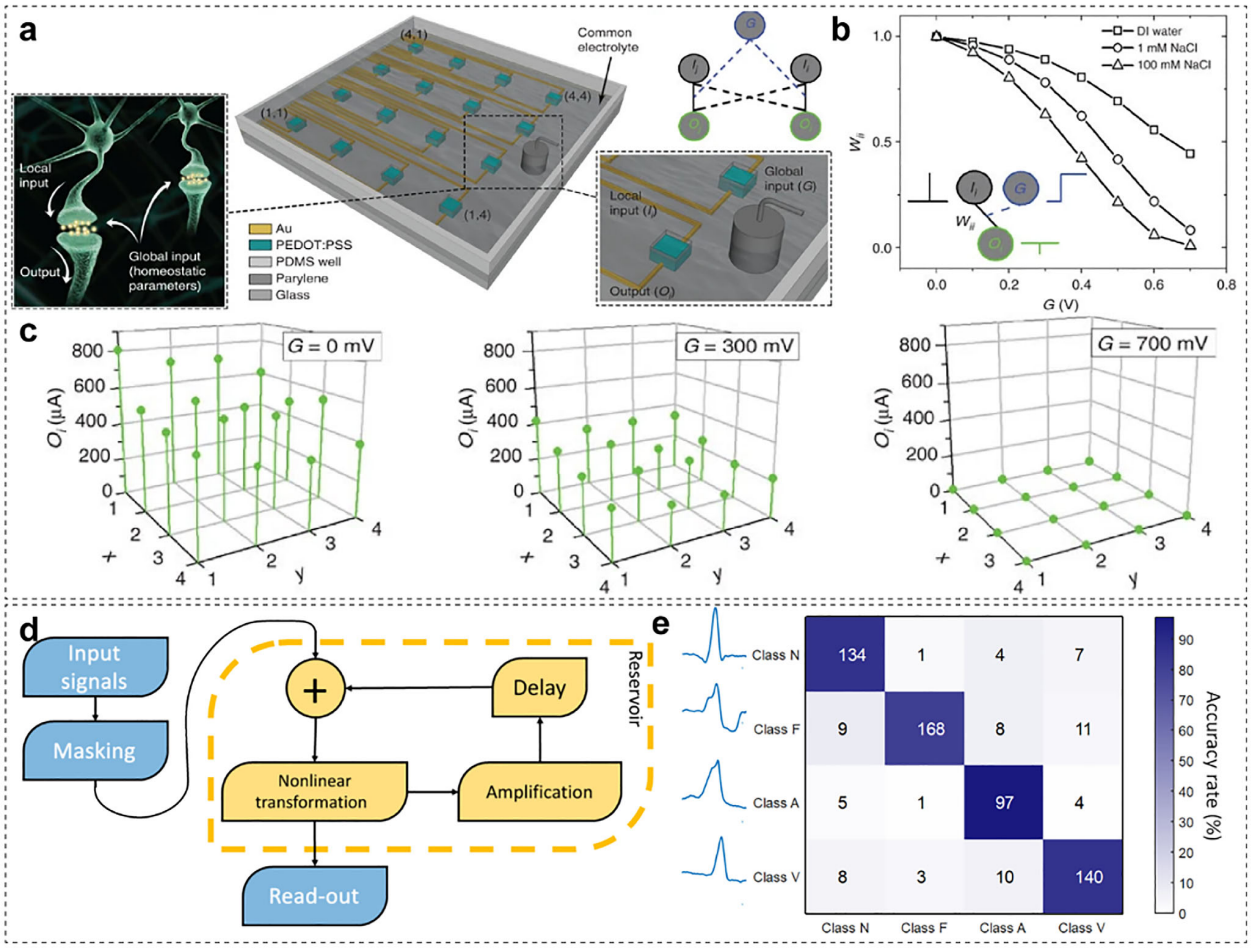
Network computation and reconfigurable learning in OECT‐based systems. (a) Schematic of a 4 × 4 device array sharing a common electrolyte and global gate. (b) Modulation of effective connection weight (𝑤_i__i_) by global gate voltage and electrolyte concentration. (c) Spatial output maps showing uniform suppression of network activity under increasing global bias. Reproduced under the terms of the CC‐BY license [155]. Copyright 2017, The Authors, published by Springer Nature. (d) Reservoir architecture leveraging nonlinear ionic dynamics and delay‐line recurrence. (e) Confusion matrix demonstrating high‐accuracy arrhythmic heartbeat classification. Reproduced under the terms of the CC‐BY license [186]. Copyright 2021, The Authors, published by AAAS.

Arrays of PEDOT:PSS devices immersed in a common electrolyte establish a hybrid connectivity framework that combines hardwired electrical pathways with soft ionic coupling. In these systems, a global gate electrode regulates the conductance state of the entire array (Figure 29a). The effective connection weight (𝑤_i__i_), defined as the normalized output current under global gating, can be continuously tuned by the gate bias and electrolyte ion concentration (Figure 29b). Spatial activity maps further reveal that increasing global gate voltage uniformly suppresses device output across the network (Figure 29c), thereby enforcing global normalization constraints analogous to homeostatic regulation observed in biological neural systems [155]. Importantly, the electrolyte simultaneously mediates lateral ionic pathways between neighboring devices, enabling distributed signal propagation and coincidence detection without explicit physical interconnects. In this framework, the ionic medium functions as a dynamic regulatory layer that reshapes network gain, synchronizes activity, and adaptively reweights connections. Network computation therefore emerges from electrolyte‐governed state modulation rather than solely from engineered circuit topology.

Beyond global regulation, distributed ionic relaxation within OECT networks can directly support physical reservoir computation. Dendritic PEDOT‐based networks immersed in physiological electrolyte perform nonlinear transformation of temporal inputs through ionic–electronic co‐transport (Figure 29d). The intrinsic relaxation time associated with ion migration provides a natural fading‐memory mechanism, while analog delay‐line feedback enhances recurrence and expands the effective dimensionality of the network state space. These collective ionic dynamics enable high‐dimensional projection of biosignals, allowing accurate real‐time classification of arrhythmic heartbeat patterns (Figure 29e) without internal training of the reservoir layer [186]. In this framework, electrolyte‐driven ionic kinetics generate separable trajectories in state space, whereas only a linear read‐out stage requires optimization. Computation thus arises from distributed electrochemical dynamics, positioning the electrolyte not merely as a gating medium but as a physical substrate for temporal signal processing.

In parallel, structural engineering of vertical solid‐state OECTs provides an additional pathway toward functional reconfiguration between neuromorphic learning and digital logic operation. In top‐frame drain geometries, the drain structure introduces an ion transport barrier that induces gradual polyelectrolyte penetration and progressively expanding hysteresis during repeated gate sweeps, producing memory‐like behavior analogous to synaptic weight evolution. In contrast, bottom‐frame drain configurations eliminate this ionic barrier, enabling rapid ion exchange and low‐hysteresis switching suitable for Boolean logic operation [59]. Through controlled manipulation of ionic transport pathways, identical device platforms can therefore transition between hysteretic learning regimes and fast‐switching transistor modes.

The computational performance of OECT‐based reservoir systems is closely linked to material properties and device architecture. In particular, the ionic relaxation time constant (*τ*_ion_ ∝ *d* ^2^/*D*_ion_) governs the fading‐memory window that underpins temporal information processing, while higher µC* and transconductance can improve the separability of reservoir states at the readout stage. Consequently, channel thickness, ionic mobility, and electrolyte configuration serve as important design parameters for tuning memory depth, nonlinear dynamics, and classification performance without modification of the readout layer.

Taken together, electrolyte‐mediated global regulation, intrinsic reservoir processing, and geometry‐controlled synapse logic transitions converge on a unified design principle in which ionic and electronic co‐transport acts as a programmable network substrate. Across these architectures, adaptive connectivity, temporal memory, nonlinear projection, and functional reconfiguration arise from controllable ionic motion rather than fixed interconnect density. By transforming the electrolyte from a passive gating environment into an active computational resource, OECT‐based systems establish a neuromorphic paradigm in which network intelligence is distributed, dynamically regulated, and structurally reconfigurable. Such hybrid analog–digital platforms offer a scalable pathway toward learning systems with minimal wiring overhead and intrinsic compatibility with biological environments.

While electrolyte‐mediated ionic coupling can enable biologically inspired distributed information processing, it also introduces a significant challenge for densely integrated OECT arrays: parasitic ionic crosstalk. Unlike conventional electronic crossbars, where interference primarily arises from leakage currents and sneak‐path conduction, OECT arrays may experience unintended ion diffusion through shared electrolytes. As device pitch decreases and array density increases, such ionic coupling may perturb neighboring conductance states, reducing programming accuracy, conductance resolution, and long‐term state stability.

Several strategies have been proposed to mitigate this challenge. Patterning the electrolyte into device‐isolated regions can eliminate long‐range ionic pathways and spatially confine electrochemical modulation to individual cells; photo‐patternable solid‐state electrolytes have demonstrated lithographic isolation of individual device electrolytes at micrometer‐scale precision, directly suppressing inter‐device ion diffusion while maintaining high ionic conductivity within each gating volume [187]. In addition, ion‐blocking trenches, insulating barriers, and encapsulated sidewalls can increase the effective lateral diffusion distance and physically isolate neighboring electrochemical environments. At the array architecture level, inkjet‐printed electrolytes with programmable 3D geometries allow the thickness and footprint of each electrolyte element to be independently tuned, enabling spatially controlled ion dynamics across the array without shared ionic reservoirs [161]. Material‐level approaches, including crosslinked ion gels and low‐diffusivity solid electrolytes, further suppress parasitic ion migration, although often at the expense of switching speed, a trade‐off that parallels the volumetric scaling constraints discussed in Section 2.1.2. Collectively, these strategies suggest that high‐density OECT neuromorphic arrays will require co‐optimization of electrolyte geometry, ionic transport properties, and device layout to preserve local volumetric gating while minimizing unintended inter‐device coupling.

### 4.4 Closed‐Loop Bioelectronic Systems

The integration of sensing, synaptic plasticity, and nonlinear ionic–electronic computation in OECT‐based platforms provides a natural foundation for closed‐loop bioelectronic systems. In these architectures, physiological signals are detected, locally processed through device‐level neuromorphic dynamics, and translated into adaptive stimulation or actuation in real time. Unlike open‐loop paradigms in which stimulation parameters are predefined and static, closed‐loop systems continuously adjust their output in response to ongoing biological states, thereby enhancing energy efficiency, selectivity, and therapeutic precision. The intrinsic coupling between ionic transport and electronic conduction uniquely enables OECTs to co‐localize detection, computation, memory, and control within a single material framework.

**FIGURE 30:**
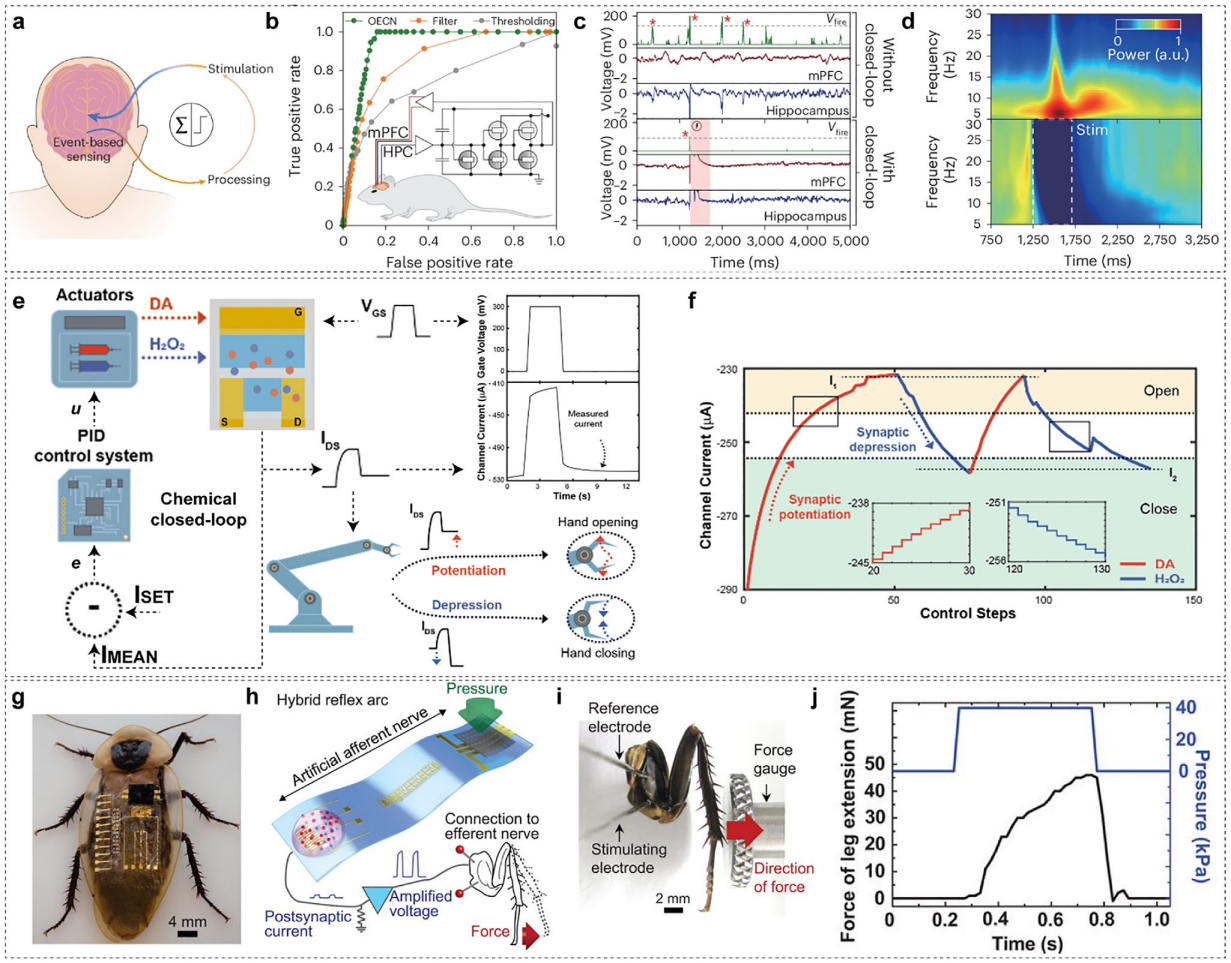
Closed‐loop OECT‐based neuromorphic bioelectronic systems. (a) Schematic of OECN‐based closed‐loop architecture implementing device‐level summation and thresholding. (b) ROC comparison demonstrating accurate IED detection. (c) In vivo traces showing IED‐triggered stimulation under closed‐loop operation. (d) Time–frequency spectrogram confirming suppression of pathological spindle activity. Reproduced under the terms of the CC‐BY license [188]. Copyright 2026, The Authors, published by Springer Nature. (e) Block diagram of PID‐regulated chemical feedback controlling ENODe conductance via DA/H_2_O_2_ release. (f) Closed‐loop convergence of channel current to programmed setpoints enabling robotic hand actuation. Reproduced under the terms of the CC‐BY license [170]. Copyright 2024, The Authors, published by Royal Society of Chemistry (RSC). (g) Artificial afferent nerve integrated on a cockroach. (h) Schematic of hybrid reflex arc linking artificial sensory encoding to biological motor nerves. (i) Experimental setup for nerve stimulation and force measurement. (j) Muscle contraction force induced by pressure‐triggered neuromorphic stimulation. Reproduced with permission [189]. Copyright 2018, AAAS.

A clear demonstration of electrically mediated closed‐loop operation is provided by high‐frequency OECN sensors for responsive neurostimulation [188]. As shown in Figure 30a, hippocampal electrophysiological signals are directly processed by an OECN implementing nonlinear summation and thresholding at the device level. ROC analysis (Figure 30b) confirms superior detection accuracy for interictal epileptiform discharges compared with filter‐based digital approaches, while consuming substantially lower power. Once the firing voltage crosses the threshold, stimulation is automatically delivered to the mPFC, establishing a fully hardware‐embedded feedback loop. Real‐time suppression of pathological IED–spindle coupling (Figure 30c) and reduced spindle‐band power in time–frequency analysis (Figure 30d) demonstrate functional efficacy. Here, closed‐loop behavior arises directly from intrinsic device nonlinearities, minimizing reliance on centralized digital processing.

Chemical feedback introduces an additional layer of adaptive control. In a hybrid silicon–organic platform integrating an ENODe [170], the channel current is continuously compared with a setpoint under PID regulation (Figure 30e). Dopamine‐ and H_2_O_2_‐mediated electrochemical modulation dynamically tunes channel conductance, enabling closed‐loop convergence to programmed current states (Figure 30f). Importantly, conductance serves simultaneously as a memory variable and a control signal, linking synaptic plasticity to robotic actuation. This framework further supports reinforcement learning implemented directly in hardware, extending closed‐loop bioelectronics from reactive stimulation toward chemically mediated adaptive learning.

Closed‐loop functionality can also emerge through hybrid sensory–motor integration. A flexible organic artificial afferent nerve converts mechanical pressure into frequency‐encoded spike trains via organic ring oscillators and integrates them through an ion‐gel–gated synaptic transistor (Figure 30g,h) [189]. Amplified postsynaptic currents stimulate biological efferent nerves (Figure 30i), generating measurable muscle contraction forces (Figure 30j). In this hybrid reflex arc, sensory encoding, synaptic integration, and motor actuation form a distributed bioelectronic control loop bridging artificial and living systems.

Together, these examples demonstrate that OECT‐based neuromorphic systems enable multimodal closed‐loop operation spanning electrical, chemical, and biohybrid feedback. Across all implementations, sensing, memory, nonlinear processing, and actuation are embedded within soft ionic–electronic materials rather than separated into discrete modules. This integration shifts closed‐loop bioelectronics from externally programmed stimulation devices toward intelligent, adaptive systems capable of learning and autonomous regulation under physiological and energy constraints.

While OECT‐based neuromorphic devices offer unique capabilities for sensing, memory, and adaptive computation, practical closed‐loop systems will likely require integration with conventional silicon electronics. OECTs excel at low‐voltage analog processing through coupled ionic–electronic dynamics, whereas CMOS platforms remain unmatched in digital logic, communication, and large‐scale computation. Thus, future neuromorphic hardware is expected to adopt hybrid architectures in which OECT arrays perform local sensing and adaptive signal processing, while silicon integrated circuits provide system control, data management, and decision‐making functions.

Bridging these two hardware platforms presents challenges in layout, packaging, and system architecture. Hydrated OMIEC materials and electrolytes must be integrated with rigid semiconductor components while maintaining long‐term mechanical and electrochemical stability, and efficient information exchange between analog ionic devices and digital electronic circuits requires robust mixed‐signal interfaces. Recent advances have begun to address these challenges at multiple levels. At the device level, fully organic complementary front‐end amplifiers, pairing depletion‐ and enhancement‐mode p‐ and n‐type OECTs at single‐neuron dimensions (∼20 µm) on parylene‐encapsulated flexible shanks, have achieved on‐site voltage‐to‐voltage amplification exceeding 30 dB with common‐mode rejection above 60 dB, enabling direct analog signal conditioning at the recording site prior to digital handoff [190]. The same parylene encapsulation strategy simultaneously provides electrical isolation and mechanical encapsulation of the aqueous electrochemical environment from underlying interconnects, establishing a packaging route compatible with implantable flexible biointerfaces. At the circuit level, fully organic p–n diode–transistor cross‐point arrays, fabricated using complementary p‐ and n‐type conducting polymer films in a 15‐µm‐diameter vertical stack, have demonstrated crosstalk‐free multiplexed addressing at 26‐µm pixel pitch without external silicon multiplexers, directly reducing the communication bottleneck between ionic and electronic subsystems [191]. These developments highlight promising pathways toward hybrid OECT–CMOS systems, although further progress in heterogeneous integration and hardware co‐design will be essential for translating OECT‐based neuromorphic devices into scalable closed‐loop bioelectronic platforms.

### 4.5 Neuromorphic Perception and Biohybrid Intelligence

Neuromorphic perception systems seek to transcend conventional sensing paradigms by embedding stimulus transduction, adaptive memory, nonlinear encoding, and actuation within unified material platforms. Rather than separating sensors, processors, and actuators into discrete hardware layers, emerging OECT‐based architectures co‐localize these functions through volumetric ionic–electronic coupling. This intrinsic electrochemical interaction enables compliant, low‐voltage, and biologically compatible devices in which perception is not merely detected but physically encoded into adaptive material states. Within this framework, OECT platforms provide a compelling foundation for tactile and somatosensory intelligence, where mechanical stimuli are translated into temporally structured neural representations and ultimately into adaptive system‐level responses.

#### 4.5.1 Tactile and Somatosensory Systems

**FIGURE 31:**
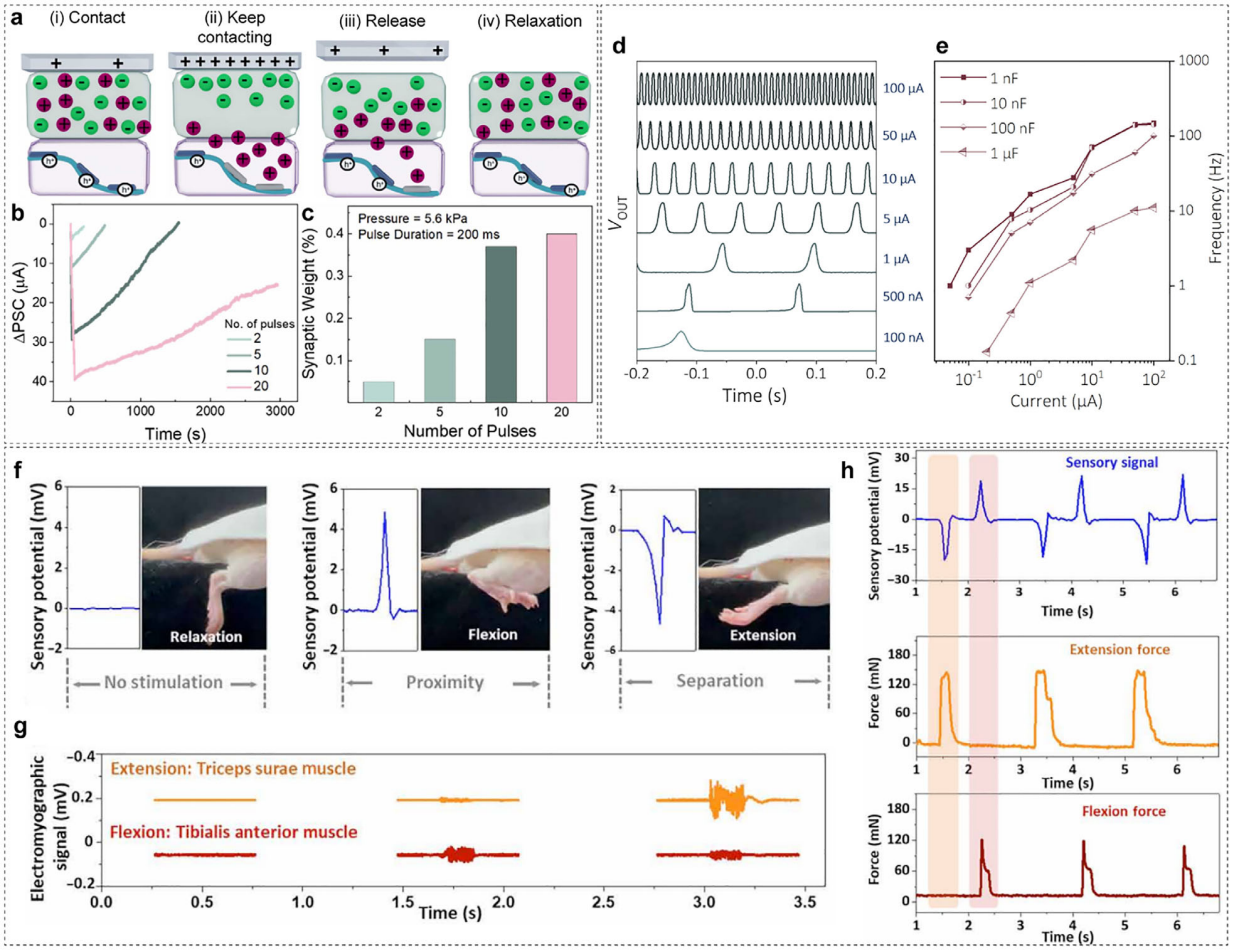
Evolution of OECT‐based tactile and somatosensory intelligence. (a) Sequential ionic polarization and channel dedoping in a triboelectric‐driven synaptic OECT under tactile stimulation. (b,c) Spike‐number‐dependent plasticity (SNDP) showing cumulative PSC and synaptic weight modulation with increasing mechanical pulses. Reproduced with permission [192]. Copyright 2024, Elsevier Inc. (d) Leaky integrate‐and‐fire spiking patterns of an OECN under varying input currents. (e) Frequency–current (*f–I*) characteristics demonstrating biologically relevant spiking range tunable by capacitance. Reproduced under the terms of the CC‐BY‐NC‐ND license [85]. Copyright 2025, The Authors, published by National Academy of Sciences (NAS). (f) Polarity‐dependent ankle flexion and extension induced by remote tactile sensing. (g) Corresponding EMG activation of antagonistic muscle groups. (h) Quantitative correlation between sensory potential and contractile force, establishing a biohybrid perception–action loop. Reproduced under terms of the CC‐BY‐NC license [193]. Copyright 2022, The Authors, published by AAAS.

The development of OECT‐based tactile systems reveals a fundamental conceptual transition from passive sensing elements toward material intrinsic somatosensory intelligence. Rather than treating sensing, memory, and actuation as sequential processing blocks, recent architectures co‐localize these functions within shared ionic–electronic dynamics. As summarized in Figure 31, this progression unfolds across three qualitatively distinct levels consisting of mechanotransductive synapses, neurons that encode firing frequency, and biohybrid interfaces that couple perception and action.

At the most elementary scale, monolithic triboelectric–OECT devices establish tactile perception as a form of electrochemical state encoding [192]. As illustrated in Figure 31a(i–iv), mechanical contact generates triboelectric charges at the ionogel interface, inducing ionic polarization that drives cation migration toward the PEDOT:PSS channel (shown as the lower navy‐colored bulk beneath the green‐colored ionogel). The resulting hole depletion modulates postsynaptic current (PSC), embedding tactile input directly into channel conductance. Crucially, this process does not append memory onto sensing. Rather, plasticity emerges from the same ionic redistribution that enables transduction.

The plastic nature of this encoding becomes evident as increasing pulse number produces cumulative Δ*PSC* and synaptic weight modulation. This spike‐number‐dependent plasticity (SNDP) arises from repeated ionic penetration and dedoping processes, mirroring the adaptive behavior of slow‐adapting mechanoreceptors (Figure 31b,c). In this architecture, mechanical parameters including force, duration, and repetition are directly mapped onto persistent conductance states. Sensing and memory are therefore co‐emergent, transforming the device from a sensor followed by memory into a unified mechanoreceptor–synapse analogue. This represents a shift from stimulus detection to material‐intrinsic tactile computation.

Yet state encoding alone does not constitute higher‐order somatosensory processing. Biological systems transform stimulus intensity into temporally structured spike trains, enabling hierarchical information transfer. This transition from state‐based encoding to temporal coding is realized in OECN circuits built from complementary vertical OECT architectures [85].

As shown in Figure 31d, increasing input current produces threshold‐triggered spiking characteristics of leaky integrate‐and‐fire dynamics. The corresponding frequency–current (*f–I*) relationship (Figure 31e) demonstrates a tunable firing range spanning 0.13 to over 140 Hz, comparable to diverse biological neurons.

Here, tactile‐derived analogue signals are no longer stored as static conductance changes but are converted into frequency‐modulated spike trains. The OECN thus performs a conceptual migration from ionic state encoding to temporal information encoding. Importantly, this frequency window can be tuned through capacitance engineering, enabling biologically plausible dynamics within soft, low‐voltage circuits. When integrated with synaptic OECTs, such neurons establish a hierarchical sensor–neuron–synapse pathway in which stimulus amplitude is translated into spike rate before synaptic filtering, paralleling natural somatosensory circuits. This architecture moves OECT systems beyond device‐level mimicry toward structured neural processing.

The most profound shift emerges when tactile interfaces are directly coupled to biological motor systems. Stretchable remote tactile devices based on conductive–dielectric heterostructures extend perception into three‐dimensional, noncontact depth‐of‐field sensing [193]. Electrostatic polarization of a high‐permittivity dielectric layer generates bipolar sensory potentials encoding proximity and separation. As demonstrated in Figure 31f, positive and negative sensory signals produce ankle flexion and extension, respectively, establishing polarity‐specific motor outputs.

Electromyographic validation confirms selective activation of antagonistic muscle groups (Figure 31g), while continuous signal profiles reveal quantitative correspondence between sensory potential amplitude and contractile force (Figure 31h). In this configuration, tactile information is not merely detected or encoded but is directly integrated into neuromuscular circuitry, thereby forming a closed loop between perception and action. The device thereby transcends emulation and becomes an augmentative interface capable of modulating biological motor behavior.

Taken together, Figure 31 delineates a coherent trajectory in OECT‐based somatosensory intelligence. Triboelectric synaptic devices establish self‐powered mechanotransduction with co‐emergent plasticity. OECN architectures introduce temporally structured spike encoding with biologically relevant firing dynamics. Remote tactile systems complete the progression by coupling artificial sensory signals directly to biological actuation. Across these scales, the unifying mechanism is the intrinsic coupling between ionic transport and electronic conduction in soft mixed conductors.

Collectively, these developments suggest the emergence of embodied electrochemical systems in which perception, memory, and actuation arise from shared ionic–electronic physics, moving beyond bioinspired component emulation.

#### 4.5.2 Visual and Optoelectronic Neuromorphic Systems

Artificial visual perception provides a stringent benchmark for neuromorphic bionic electronics because biological vision inherently integrates multi‐timescale synaptic plasticity, spike‐frequency encoding, and neuromodulator‐mediated adaptation within a hierarchically organized retinal network. OMIECs, through their intrinsic coupling between ionic flux and electronic conduction, offer a uniquely suitable materials platform for reconstructing retina‐level computations in soft and conformable architectures.

**FIGURE 32:**
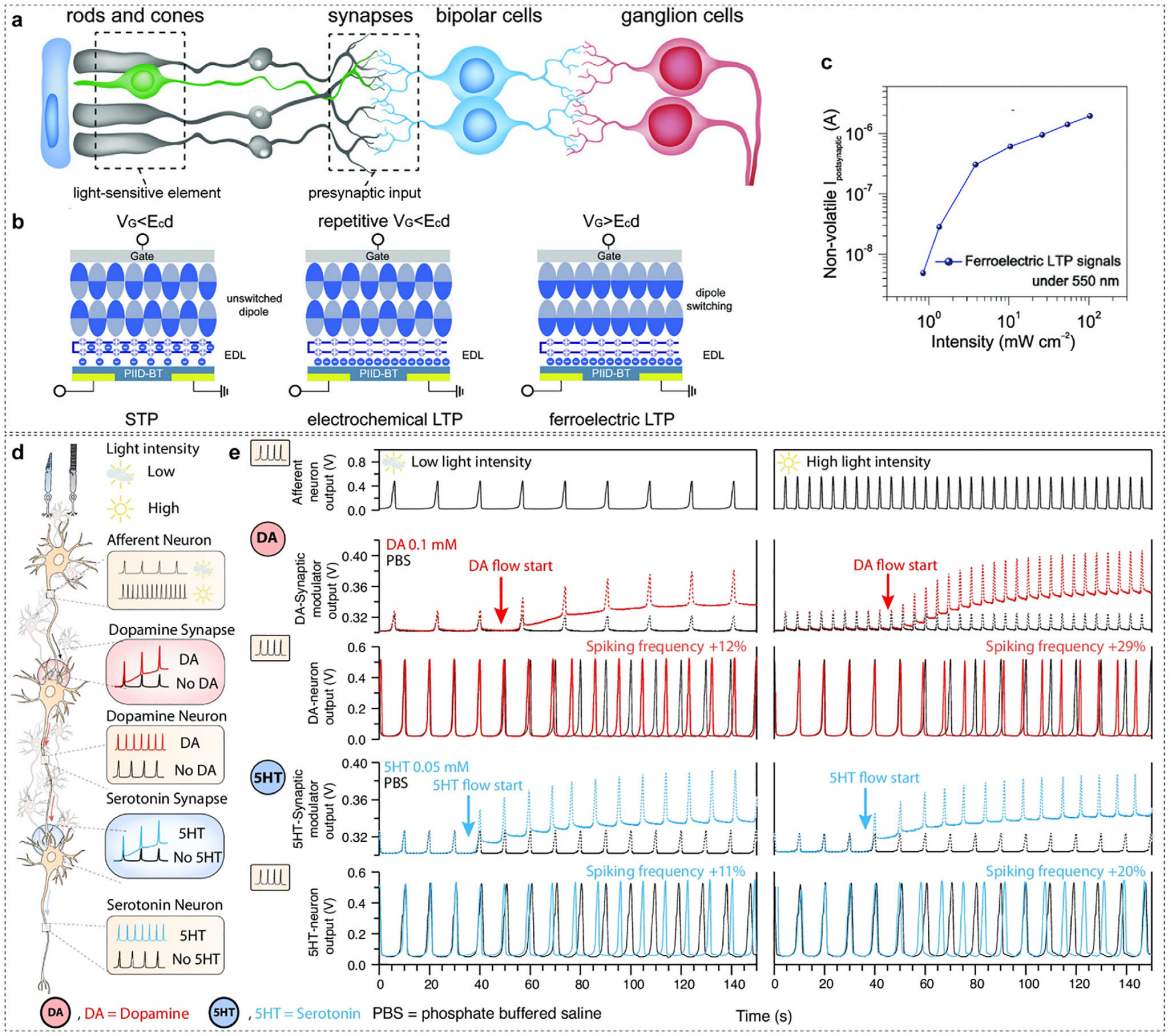
Visual and optoelectronic neuromorphic systems based on OECT platforms. (a) Schematic of the biological retina illustrating hierarchical visual signal processing from photoreceptors to neural pathways. (b) Multi‐timescale synaptic plasticity in the organic synaptic transistor, showing STP, electrochemical LTP, and ferroelectric LTP mechanisms. (c) Light‐intensity‐dependent synaptic response enabling optical stimulus–to–postsynaptic current conversion for artificial visual perception. Reproduced with permission [194]. Copyright 2018, Wiley‐VCH. (d) Retina‐inspired neuromorphic cascade architecture. (e) Light‐ and neurotransmitter‐modulated spike‐frequency response. Reproduced under terms of the CC‐BY license [171]. Copyright 2024, The Authors, published by Springer Nature.

A representative example of device‐level artificial visual memory was demonstrated using a hybrid ferroelectric/electrochemical synapse based on a bilayer P(VDF‐TrFE)/P(VP‐EDMAEMAES) gate stack [194]. Biological vision processes optical stimuli through a hierarchical retinal network in which photoreceptors convert incident photons into neural signals that are subsequently transmitted and modulated through synaptic connections among bipolar and ganglion cells (Figure 32a). Inspired by this architecture, organic neuromorphic devices have been developed to directly couple optical sensing with synaptic signal processing. A representative example is the ferroelectric/electrochemical organic synapse integrated within a light‐triggered organic neuromorphic device (LOND), which combines optoelectronic sensing with synaptic computation in a unified platform. In this system, electrochemical doping and dedoping of the channel produce volatile STP and electrochemical LTP, while ferroelectric dipole switching introduces an additional non‐volatile LTP mode with extended retention time (Figure 32b). This multi‐timescale plasticity enables the device to encode optical stimuli into adaptive synaptic responses. When integrated with a photosensitive element, the LOND converts incident light into postsynaptic currents whose magnitude depends on the intensity of illumination, enabling grayscale visual perception through tunable synaptic weight modulation (Figure 32c). Such optoelectronic neuromorphic platforms illustrate how OMIEC‐based devices can emulate key aspects of retinal signal processing by co‐localizing light sensing, memory formation, and synaptic computation within soft and conformable electronic systems.

Complementary to ferroelectric‐assisted approaches that integrate discrete photodetector–synapse stacks, a material‐intrinsic route toward retina‐inspired opto‐ionic computation was recently demonstrated through an organic iono‐optoelectronic synapse based on a p‐type OMIEC photoelectrochemical transistor architecture. In this system, the donor–acceptor polymer gDPP‐MeOT2 simultaneously serves as a broadband light absorber and ion reservoir within an ionic liquid gel electrolyte, enabling monolithic coupling of optical excitation, ionic redistribution, and electronic transport in a single mixed‐conducting film. Under visible‐to‐near‐infrared pulsed illumination, the device exhibits canonical synaptic functions, including paired‐pulse facilitation and STP‐to‐LTP transitions, at ultralow programming voltages (∼0.4 V), where photogenerated carriers modulate electrochemical potentials and volumetric doping states to produce persistent conductance changes. Beyond single‐synapse behavior, the platform further demonstrated optically reconfigurable inverter operation and multispectral image denoising via wavelength‐weighted optical convolution, illustrating how photoelectrochemical OMIEC‐based OECTs can unify photodetection, adaptive synaptic plasticity, and edge‐level analogue processing within a single material framework [195].

A higher level of functional abstraction is achieved in the modular retina‐inspired neuromorphic pathway reported in [171], where OECT‐based spiking neurons built from ambipolar inverter pairs (p(C4‐T2‐C0‐EG)) and resetting electrochemical transistors (P‐3O) implement sensory coding by translating light‐induced receptor potentials into spike trains with intensity‐dependent firing frequency, thereby reproducing the frequency‐based encoding mechanism of biological afferent retinal neurons. As illustrated in Figure 32d,e, light‐induced receptor potentials are translated into spike trains whose frequency varies with illumination intensity, achieving spike‐rate modulation up to approximately 29% under high‐intensity conditions. This sensory coding is further coupled with neurotransmitter‐mediated synaptic modulation. PEDOT:PSS‐based biohybrid synaptic modulators exhibit non‐volatile conductance changes upon oxidation of electroactive neurotransmitters such as DA and 5‐HT at gate potentials exceeding their redox threshold (*V*_ox_ ≈ 0.3 V). In the presence of DA, synaptic baseline shifts of approximately 0.04–0.08 mV induce corresponding increases in dopaminergic neuron firing frequency (12%–29%, depending on illumination intensity), while subsequent 5‐HT modulation further adjusts downstream spike rates by 11%–20% (Figure 32e). Although transient spikes are superimposed on synaptic outputs, it is the baseline conductance modulation that governs downstream neuronal excitability, thereby reproducing spike‐rate coding through neuromodulation. The resulting cascade, consisting of a light sensor, afferent spiking neuron, DA‐regulated synapse, DA neuron, 5‐HT synapse, and a 5‐HT neuron (Figure 32d), reconstructs a primitive retinal preprocessing unit in which physical (optical) and biochemical (neurotransmitter) signals jointly regulate information propagation. Unlike isolated optoelectronic synapses, this modular architecture replicates interneuron‐level signal modulation, enabling pathway‐level neuromorphic computation rather than single‐device sensory response.

The former establishes intra‐device fusion of photodetection and multi‐timescale synaptic memory, including ferroelectric‐hybrid stacks and photoelectrochemical OMIEC synapses enabling grayscale perception and volatility‐selective color encoding. The latter advances toward pathway‐level neuromorphic architectures, integrating spike‐frequency sensory coding with neurotransmitter‐mediated modulation within cascaded retinal‐like circuits.

#### 4.5.3 Multimodal Learning and Robotic Intelligence

Beyond single‐modality perception, organic neuromorphic bionic electronics increasingly demonstrate embodied intelligence, in which learning emerges from real‐time interactions among multimodal sensing, adaptive ionic–electronic state evolution, and actuator feedback. Rather than executing static classification, these systems encode internal analog state variables that dynamically reweight perception–action policies directly at the hardware level.

**FIGURE 33:**
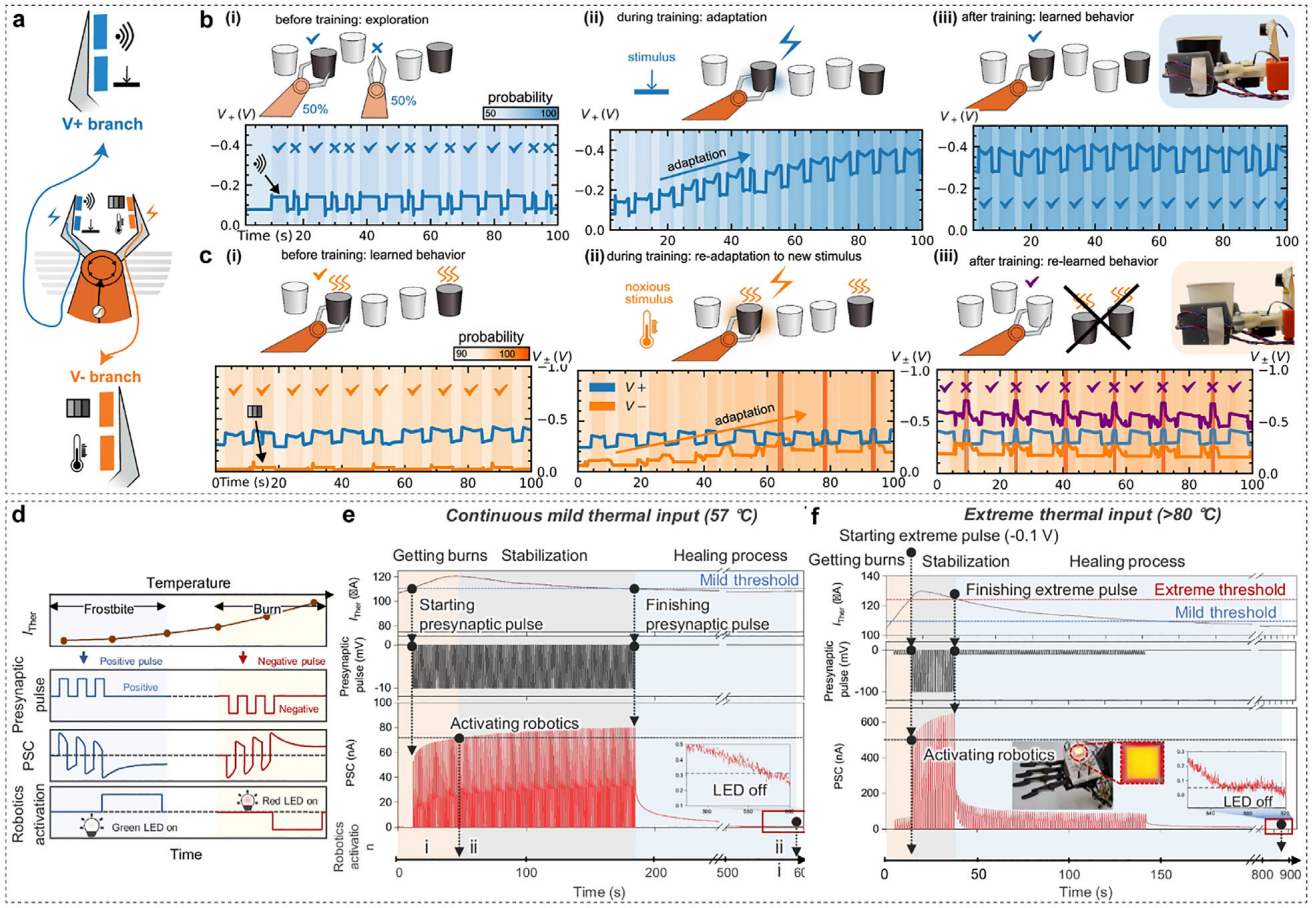
Multimodal learning and reflexive robotic intelligence enabled by organic neuromorphic bionic electronics. (a) Multimodal robotic platform integrating heterogeneous sensors with an organic neuromorphic circuit. (b) Progressive associative learning in the (+) branch: exploration (i), reinforcement‐driven adaptation (ii), and stable conditioned grasping (iii). (c) Multimodal chaining with the (−) branch: maintained prior learning (i), adaptation to thermal/color cues (ii), and context‐dependent discrimination of cold versus hot objects (iii). Reproduced under the terms of the CC‐BY license [196]. Copyright 2024, The Authors, published by Springer Nature. (d) Signal transduction pathway from temperature sensing to synaptic processing and robotic actuation within a biodegradable reflex system. (e) Gradual synaptic potentiation under mild thermal stimulation, leading to threshold‐dependent activation and a persistent memory‐like response. (f) Rapid, high‐amplitude synaptic response under extreme thermal stimulation, enabling immediate protective actuation followed by prolonged recovery dynamics. Reproduced under the terms of the CC‐BY‐NC‐ND license [197]. Copyright 2025, The Authors, published by Springer Nature.

A representative multimodal conditioning architecture integrates heterogeneous sensory inputs such as proximity and color as contact‐free cues, as well as pressure and temperature as consequence‐based signals, into a compact organic neuromorphic circuit composed of volatile OECT elements and nonvolatile electrochemical random‐access memory synapses (Figure 33a–c) [196]. Branch‐specific pathways analogous to excitatory (+) and inhibitory (−) channels enable associative coupling between stimuli and behavioral outcomes.

In the initial exploratory regime (Figure 33b(i))), grasping actions occur probabilistically and remain uncoupled from environmental cues. During reinforcement (Figure 33b(ii), coincident proximity detection and pressure feedback induce conductance modulation in the nonvolatile synaptic element, progressively shifting the branch voltage and modifying the activation probability. After sufficient training (Figure 33b(iii)), proximity alone reliably triggers grasping behavior, reflecting hardware‐level associative conditioning.

The learning process subsequently expands through behavioral chaining (Figure 33c(i–iii)). When thermal and color stimuli are introduced, previously acquired proximity‐driven behavior is preserved (Figure 33c(i)), demonstrating memory stability. Adaptation of the complementary branch (Figure 33c(ii)) establishes an associative link between temperature and color. In the fully trained state (Figure 33c(iii)), the summed branch outputs generate context‐dependent discrimination in which cold (white) objects are grasped whereas hot (dark) objects are avoided. This progression from exploratory behavior to single modality conditioning and finally to multimodal operant learning illustrates how ionic and electronic conductance states function as persistent internal variables that reshape robotic decision‐making without centralized digital control.

A complementary strategy emphasizes sustainable and energy‐efficient reflexive intelligence through a biodegradable multilayer artificial synapse (Figure 33d–f) [197]. In this system, temperature stimuli are converted into presynaptic voltage pulses that drive PSC generation in a multilayer artificial synaptic element (Figure 33d). The ion‐active/ion‐binding/ion‐active trilayer architecture exploits ion–dipole coupling to enable partial ion retention and progressive PSC evolution under sub‐millivolt operation.

Stimulus severity is encoded through graded synaptic potentiation. Under mild thermal input (57°C), prolonged low‐amplitude stimulation gradually elevates PSC beyond an activation threshold, triggering protective actuation only after sufficient accumulation (Figure 33e). In contrast, extreme thermal exposure (>80°C) produces rapid PSC escalation and immediate full‐actuator activation (Figure 33f). Notably, the persistence of PSC following stimulus removal maps long‐term potentiation dynamics onto an effective healing timescale, thereby embedding memory directly into reflex duration. Thus, adaptive response amplitude and recovery time are both governed by ionic retention dynamics.

Collectively, these demonstrations indicate that organic neuromorphic platforms implement robotic intelligence across hierarchical levels, spanning ionic transport physics and control of volatile and nonvolatile conductance states, circuit‐level summation and thresholding, and ultimately closed‐loop behavioral shaping in physical environments. Multimodal learning in these systems is not a simple fusion of sensor outputs. It is the formation and continuous reconfiguration of internal analog state variables stored in ionic and electronic conductance and interfacial polarization. Advancing this paradigm will require scalable branch architectures, device‐level training rules embedded in material dynamics, and sustainable materials platforms compatible with long‐term biohybrid deployment.

## 5 Conclusions and Outlook

Collectively, the OMIEC and OECT literature converges on a central principle in which neuromorphic behavior in organic electrochemical systems does not arise from complex peripheral circuitry but instead emerges directly from the coupled transport of ionic and electronic charges within hydrated, chemically active materials. Organic mixed ionic–electronic conductors are specifically engineered to enable this interaction, allowing ionic motion from an electrolyte to modulate the electronic carrier density throughout the channel volume. As a result, OECT conductance becomes a history‐dependent state variable governed by electrochemical doping and dedoping dynamics rather than a binary electronic switch. Across diverse materials systems and device architectures, a consistent set of parameters defines the operational envelope of neuromorphic functionality, including the accessible conductance window and its analog linearity, the balance between retention and volatility that determines memory persistence, and the characteristic time constants dictated by ion transport, volumetric capacitance, and electronic conductivity. These properties collectively establish the dynamical behavior necessary for synaptic plasticity, temporal filtering, and adaptive signal processing. Importantly, volumetric electrochemical charging provides high capacitance and intrinsic amplification but simultaneously couples device bandwidth and response time to the transport rates of ions and electronic carriers within the channel, creating an inherent thickness–gain–speed trade space that necessitates deliberate engineering. Recent transport analyses further clarify that OMIEC channels can be described by transmission‐line models in which conductivity and volumetric capacitance govern signal attenuation and phase delay, directly linking mixed‐conduction material properties to the fidelity of physiological signal processing. Within this framework, attributes traditionally described as biocompatibility, including electrolyte stability, controlled swelling, and reversible ion uptake, emerge not as secondary packaging considerations but as primary determinants of stable neuromorphic dynamics.

Finally, manufacturing is increasingly inseparable from device physics in neuromorphic bioelectronics. Additive and hybrid fabrication techniques offer unique opportunities to directly program channel volume, electrolyte geometry, and mechanical boundary conditions, effectively transforming fabrication into a physical design layer that defines ionic dynamics and computational behavior. While challenges remain in printing resolution, ink rheological requirements, and limited scalability, recent demonstrations of high‐resolution printed OECTs, fully 3D‐printed device stacks, and printed circuit‐level architectures indicate that manufacturable neuromorphic biointerfaces are transitioning from conceptual demonstrations toward experimentally viable technologies. Future progress will therefore depend on integrating materials design, device physics, and manufacturing strategies into a unified framework in which mixed ionic–electronic transport is deliberately engineered to match the temporal and adaptive requirements of neuromorphic computation in biological environments.

To accomplish continued progress in OECT‐based neuromorphic bioelectronics, several of the most important challenges facing the field must be overcome. Among these, long‐term operational stability remains a vital requirement for the practical implementation of OECT‐based neuromorphic devices. Although OMIECs exhibit excellent performance in aqueous environments, prolonged operation can lead to material degradation associated with swelling, repeated doping/dedoping processes, limited chemical stability in biological fluids, and dehydration of hydrogel‐based materials. Addressing these challenges will require materials designs that combine low swelling, high chemical stability, and long‐term structural robustness. Closely related to this issue is biointerface reliability. Not only the intrinsic stability of the active materials but also stable interactions between devices and biological tissues must be maintained over extended periods. Future bioelectronic interfaces should minimize mechanical mismatch with soft tissues, provide reliable adhesion, suppress adverse inflammatory responses, and maintain functionality under chronic implantation conditions. Furthermore, beyond material and biointerface considerations, complementary OECT integration represents another important challenge from device and circuit perspectives. Most reported OECT circuits are currently dominated by p‐type OMIECs, whereas the realization of low‐power and highly integrated neuromorphic circuits requires substantial improvements in n‐type materials. In particular, improving environmental stability under ambient and aqueous conditions and establishing processing compatibility between p‐type and n‐type materials for integrated circuit fabrication will be essential for future progress.

Ultimately, these advances should converge toward the integration of sensing, memory, and computing within a single neuromorphic bioelectronic platform. Conventional electronic systems typically separate sensing, memory storage, and information processing into distinct functional units. In contrast, OECTs uniquely combine ionic transduction, electrochemical memory effects, signal amplification, and neuromorphic functionalities within the same device architecture. This distinct characteristic provides opportunities for the development of adaptive and intelligent neuromorphic bioelectronic systems capable of sensing, learning from, and processing biological signals in real time. Taken together, future progress in OECT‐based neuromorphic bioelectronics is expected to proceed from achieving long‐term operational stability, to establishing reliable biointerfaces, to realizing complementary circuit architectures, and ultimately to the development of intelligent neuromorphic bioelectronic systems that integrate sensing, memory, and computing within a unified platform.

## Author Contributions

H.L. and J.P. contributed equally to this work. H.L., J.P., and T.Z. conceptualized the review and wrote the manuscript. All authors, H.L., J.P., X.W., Y.D., J.R., and T.Z., contributed to the discussion of the content and the revision of the manuscript.

## Conflicts of Interest

The authors declare no conflicts of interest.

## Data Availability

This is a review article, and no new data or materials were generated.
